# Tactical vs. strategic: An adaptable framework for horizon-dependent dengue forecasting using data-driven approaches with serotype and climate covariates in Bangladesh

**DOI:** 10.1371/journal.pone.0353069

**Published:** 2026-08-21

**Authors:** Md. Muqtadir Fuad, Md. Sajib Milki, Ridwan Al Aziz

**Affiliations:** Department of Industrial and Production Engineering, Bangladesh University of Engineering and Technology, Dhaka, Bangladesh; University of Dhaka, BANGLADESH

## Abstract

Dengue remains a major public health problem in endemic regions, including Bangladesh. Forecasting algorithms relying on climatic variables may not capture epidemiological information such as dengue serotype patterns. This study proposes a horizon-dependent dengue forecasting framework and applies it to Bangladesh. This pipeline included temporal, meteorological, demographic, and epidemiological covariates in a district-panel pipeline and compares classical, machine-learning, and deep-learning algorithms using aggregate, horizon-wise, regime-wise, outbreak-detection, and uncertainty metrics. Coefficient-based interpretation, SHAP, permutation importance, and nonlinear causal-dependence analysis were used to examine predictor impacts across horizons. SARIMAX was used as the baseline model. TFT produced quantile forecasts but showed poor interval calibration during outbreak periods. Results show that no single model performed best across all forecasting horizons: SARIMAX ranked highest for one-step outbreak alerting (precision = 0.886, recall = 0.824, F1 score = 0.854, and ROC-AUC = 0.950), whereas Prophet performed best for pooled magnitude forecasting at horizons 2–6. MLR showed competitive performance in regime-wise evaluation. These findings show that dengue forecasting algorithms should be selected according to the intended decision objective and evaluated using task-relevant protocols.

## Introduction

In tropical and subtropical regions globally, dengue fever has emerged as an intimidating public health concern. In the humid climate and dense tropical terrain of Bangladesh, this threat is acute. According to a World Health Organization report, approximately 100–400 million people are infected by this virus worldwide every year [1]. The number of dengue infections is increasing compared to the dawn of this millennium in this territory (e.g., the number reported in 2023 was 1.3 times higher than the cumulative number of patients in the previous 23 years) [2,3]. Researchers are working on developing effective dengue vaccines [4,5] but they have certain limitations to their effectiveness [6,7]. One method to improve this situation involves harnessing predictive and mathematical models [8–12] for proper resource allocation. However, a critical gap remains in examining the relationship between specific viral strains (serotypes) and weather anomalies using interpretable deep learning. To bridge this gap, this paper proposes a multi-horizon forecasting framework and explores the use of predictive modelling [13]. Although forecast performance is not guaranteed, such models will help public health authorities to better target vector control. [14]. The present study is guided by the following research questions:

**RQ1.** How do statistical, machine learning, and deep learning models compare in forecasting dengue incidence across multiple horizons (1–6 months ahead)? Is there a single model that consistently outperforms others in all scenarios?
**RQ2.** Beyond overall accuracy, how effectively can these models predict the magnitude of dengue outbreaks during high-transmission periods?
**RQ3.** Which epidemiological and environmental variables are most important for the best-performing models, and does their relative importance remain stable or change over time?

The focus of modelling has shifted from seasonality-based time series forecasting to machine learning and deep learning algorithms [15,16]. In our case study, early works used statistical and univariate time series models for monthly dengue data in Dhaka and established a baseline with simplified assumptions and limited inputs [17–24]. Recent studies have used machine learning and deep learning for dengue prediction [25,26]. Several studies have developed machine learning and deep learning models using sociological, economic, climatic, and demographic variables [27–35]. In Bangladesh, forecasting mostly relied on univariate time-series or climate-based regression, with limited epidemiological covariates [27,28]. We may close this gap further by combining serotype and epidemiological data as additional variables. Rainfall, temperature, humidity, demographic change, immune landscape, and viral evolution affect epidemic magnitude [36–41], along with temporal association [27,42–46].

Although progress in ML-based dengue forecasting is on the way, the incorporation of viral information like serotype (DENV-I-IV) or serotype dominance and genetic evolution into predictive models is yet to be explored. Serotype shifts can affect epidemiological patterns by influencing immunity and disease severity [47,48]. The introduction of new viral strains from exchanges between Southeast Asia and India raised the risk of outbreaks [49]. The outbreaks in 2019, 2022, and 2023 in Bangladesh illustrate this gap. The 2019 epidemic was amplified by a recent genotype shift [27], while a shift toward DENV-2 dominance in 2022 was linked to enhanced transmissibility [33,50]. Machine learning approaches including Prophet, as well as deep learning architectures such as Temporal Fusion Transformer (TFT) or attention-based LSTMs, have been studied elsewhere, their capabilities for long forecasting horizons have not been explored in Bangladesh [34,51]. TFT, a Transformer-based model, is suitable for multi-horizon forecasting in endemic regions due to its attention mechanism [48,52]. Very limited research in this region has used TFT, ATT-LSTM, or Prophet for predicting dengue patient counts over various time frames with serotype and epidemiological data as covariates.

## Methodological framework and case study setup

### The adaptable forecasting framework

**Fig 1:**
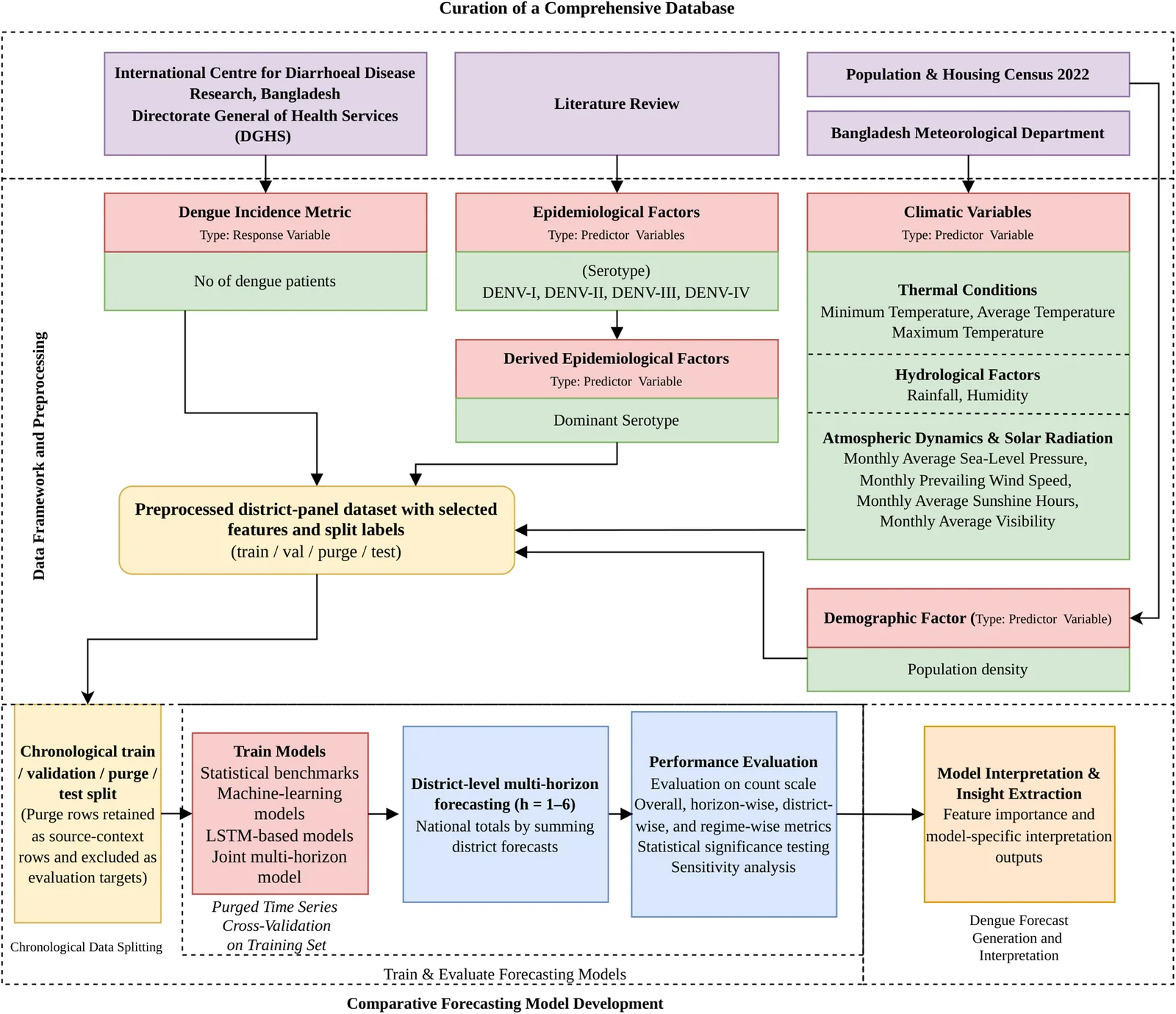
Adaptable forecasting framework for district-panel dengue prediction in Bangladesh. The framework integrates epidemiological, climatic, demographic, and census data into a district-panel dataset with selected features and chronological split labels.

We propose an adaptable framework (shown in Fig 1) for multi-horizon dengue forecasting. The framework combines: (1) a common district-panel forecasting pipeline; (2) serotype, climatic, demographic, and epidemiological variables; (3) sensitivity analysis under controlled model perturbations; and (4) causal inference using Granger causality and PCMCI to examine predictive drivers.

### Case study setting: Bangladesh

**Fig 2:**
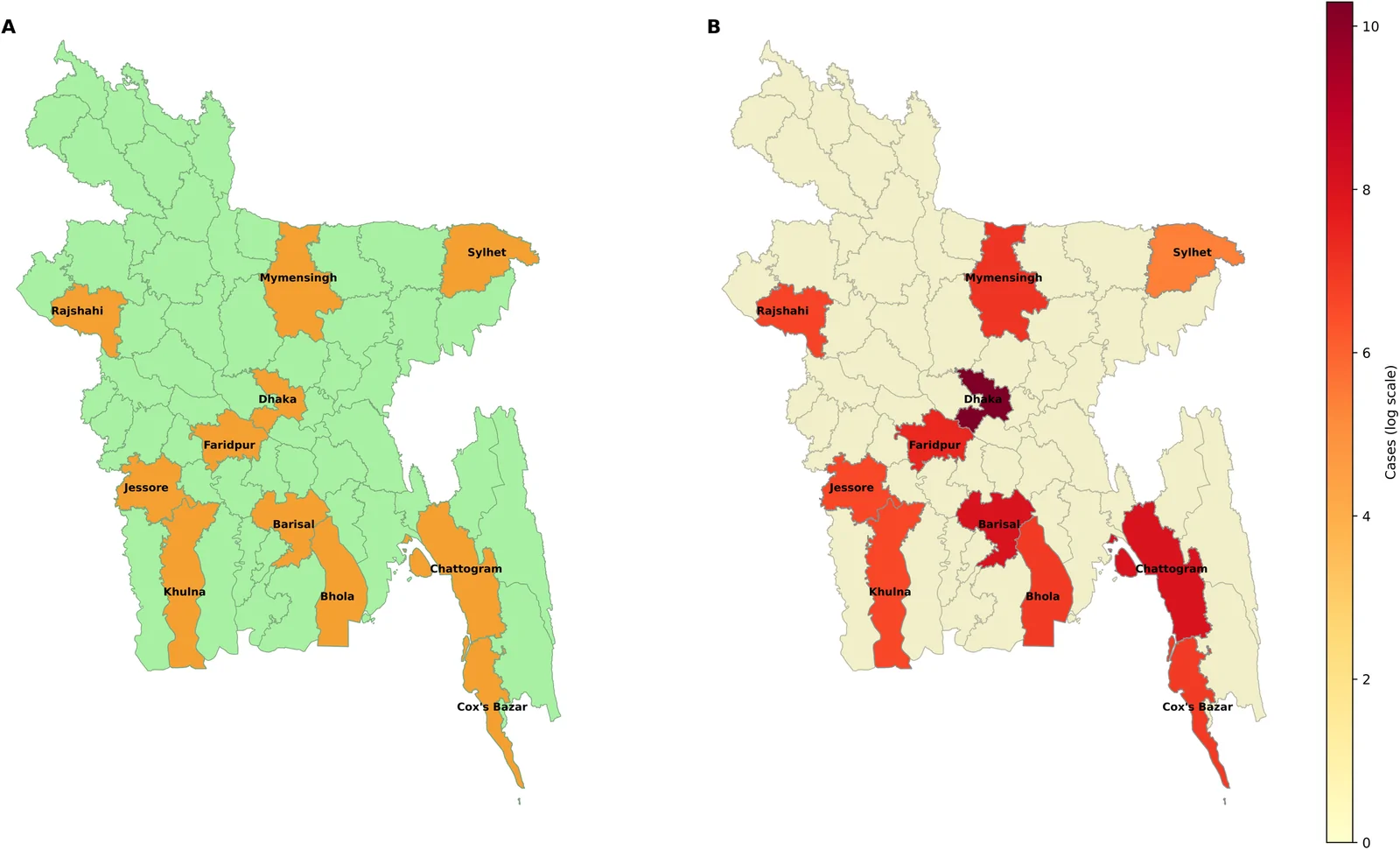
Geographical data used for dengue analysis and visualization in Bangladesh. **(A) Selected study districts used for ML forecasting. (B) Log-scaled dengue cases in Bangladesh in September 2023.** Administrative boundaries are based on open data from the Humanitarian Data Exchange (HDX) [53].

This framework was implemented for monthly dengue forecasting in Bangladesh (selected districts shown in Fig 2). The following sections describe the data sources, preprocessing steps, feature engineering, feature selection, and forecasting models. The modeling pipeline used a chronological train-validation-purge-test split and generate forecasts for horizons of 1–6 months ahead. SARIMAX was used as the baseline model.

### Dataset development for comprehensive analysis

The data for this analysis was curated from Bangladesh Meteorological Department [54], annual census publications [55], and findings reported in peer-reviewed literature. The Directorate General of Health Services (DGHS) digital repository was used as the main source of dengue incidence data [56]. We assembled a monthly panel of 924 observations covering 11 districts of Bangladesh from January 2017 to December 2023. These districts are considered as a representative of entire Bangladesh due to their spatial distribution [31] Temperature (including lagged effect) shows a nonlinear and delayed influence on the dissemination of dengue [57–59]. Higher relative humidity is correlated with elevated dengue case [60,61]. Short-term studies in South and Southeast Asia show that moderate rain creates breeding sites [62]. Reduced sunshine before the monsoon has also been associated with larger annual epidemics in Bangladesh [46]. Population density was included because it increases human–vector contact opportunities [3,55,63]. Additionally, dengue serotype dynamics may affect epidemic behavior in Bangladesh and neighboring South Asian settings [64–66]. Hence, we included the four dengue serotypes as epidemiological covariates and derived a categorical *dominant serotype* feature. Serotype information was available at annual resolution; therefore, annual DENV-I–DENV-IV values (%) were assigned to all monthly observations within the corresponding year.The categorical dominant serotype variable was constructed by identifying the serotype with the highest annual percentage value for that year.

### Feature engineering

**Fig 3:**
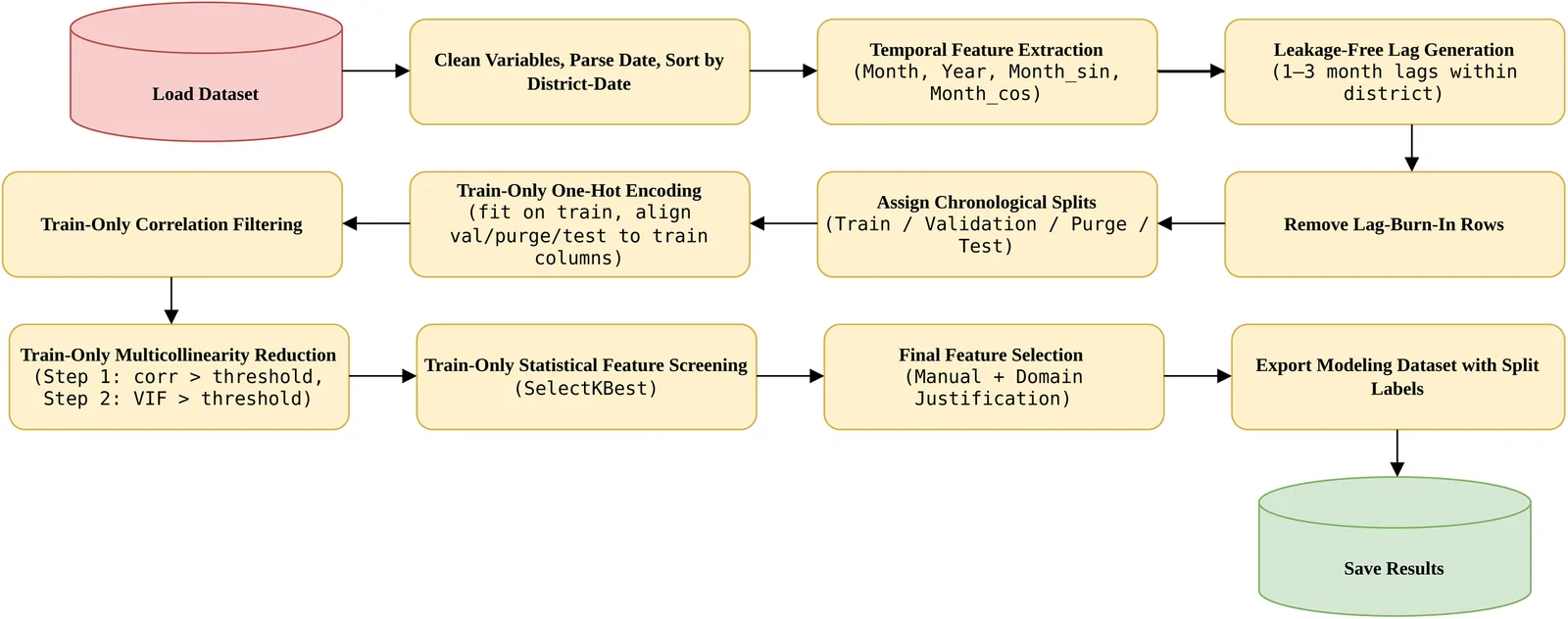
Feature-engineering and preprocessing workflow.

Feature engineering (shown in Fig 3) began with strict date parsing and district-wise chronological ordering. From the parsed date, month and year were extracted, and seasonality was represented by sine and cosine encodings of month. The target variable was the log-transformed monthly dengue count, defined as $\log(1+c_{t})$. To preserve temporal validity, 1-, 2-, and 3-month lag features were generated within each district for numeric predictors, including serotype shares, meteorological variables, and dengue count, and for categorical predictors (dominant serotype and prevailing wind direction). For serotype variables, lag features were generated after annual-to-monthly alignment. Lag-induced missing values were not imputed; instead, the initial lag burn-in rows were removed. The resulting dataset was split chronologically into train, validation, purge, and test periods. Categorical variables were then one-hot encoded using the training set, and the remaining splits were reindexed to the same encoded feature space.

### Feature selection

**Fig 4:**
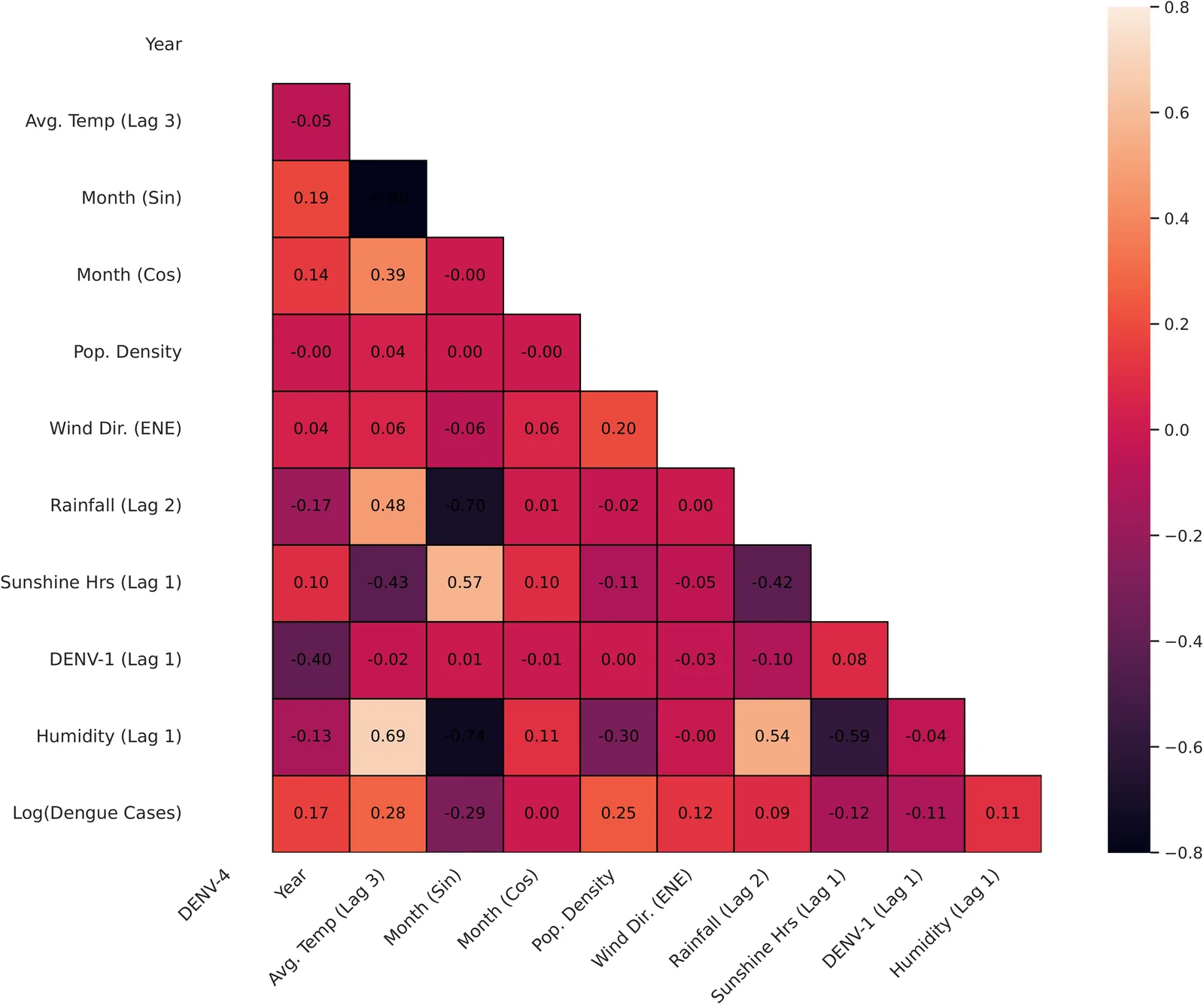
Correlation matrix of the selected predictors and the log-transformed dengue target. The lower triangular matrix shows pairwise Pearson correlation coefficients.

Feature selection was performed after preprocessing and categorical encoding. First, numeric predictors were screened by pairwise correlation (shown in Fig 4), using a threshold of 0.90. When two variables were highly correlated, protected variables were retained; otherwise, the variable with the weaker absolute correlation with the target was removed. The remaining predictors were then filtered by variance inflation factor (VIF), with iterative removal of non-protected variables above the threshold of 10.0. The reduced feature set was next ranked using SelectKBest with the F-test for regression, and the top 35 variables were retained as a screening set. Final predictors were then chosen from this screened set to retain a non-redundant and temporally available group of variables covering epidemiological, climatic, temporal, and demographic domains. The final feature set comprised *DENV-4*, year, average temperature (lag 3), month sine, month cosine, population density, prevailing wind direction (ENE), rainfall (lag 2), sunshine hours (lag 1), *DENV-1* (lag 1), and humidity (lag 1).

### Standard hierarchical approach

A common district-panel forecasting framework was used across the selected models (used mathematical notations shown in Table 1). The response variable was the monthly dengue count, transformed as $y_{t}=\log(1+c_{t})$. Model outputs were converted back to the count scale as ${\hat{c}}_{t}=\max\{0,\exp({\hat{y}}_{t})-1\}$ for evaluation. All modeling inputs were sorted chronologically by district and date. The prepared dataset already contained four ordered split labels: train, val, purge, and test. Purge rows were retained in the modeling file because they serve as source-context rows for higher-horizon forecasting, but they were not used as evaluation targets.

**Table 1:** Notations used in this study.

Symbol	Description
*t*	Source month index.
*h*	Forecast horizon, h∈{1,...,6}.
$c_{t}$	Observed dengue count at month *t*.
$y_{t}$	Log-transformed dengue count, $y_{t}=\log(1+c_{t})$.
${\hat{y}}_{t+h}$	Predicted log-transformed count at horizon *h*.
${\hat{c}}_{t+h}$	Predicted count after back-transformation.
${𝐱}_{t}$	Predictor vector at month *t*.
${𝐱}_{t-k+1:t}$	Predictor sequence from t−k+1 to *t*.
${𝐳}_{t+h}$	Target-month covariates for LSTM-based models.
$\chi_{t+1:t+6}$	Known future covariates for TFT.
*k*	Look-back or encoder length.
*q*	Quantile level.
$\theta_{0.90}$	Outbreak threshold from the 90th training percentile.
*N*	Number of evaluated observations.
*D*	Number of districts.
$T_{d}$	Number of training months for district *d*.
$\ell_{d,t,h}^{\left(r\right)}$	Reference-model loss for district *d*, date *t*, horizon *h*.
$\ell_{d,t,h}^{\left(c\right)}$	Competitor-model loss for district *d*, date *t*, horizon *h*.
${\bar{d}}_{t,h}$	Mean loss differential at date *t*, horizon *h*.
$DM_{h}^{*}$	HLN-corrected Diebold–Mariano statistic at horizon *h*.

Abbreviation: TFT, Temporal Fusion Transformer.

Forecasts were generated for horizons h∈{1,...,6}. For direct multi-horizon models, each source row at time *t* was paired with a target a*t t* + *h*. This setup was used for MLR, SVR, RF, XGBoost, CatBoost, stacked LSTM, and attention-based LSTM. In these models, horizon-specific seasonal information was represented by TargetMonth_sin and TargetMonth_cos, derived from the forecast target month. The source-row temporal variables Year, Month_sin, and Month_cos were excluded where target-month seasonality replaced them. TFT followed a joint multi-horizon design, where a single model predicted all horizons up to six months ahead using a 12-month encoder and known future temporal covariates. Prophet and SARIMAX were implemented as grouped district-level benchmarks, with one model fitted per district and forecasts later aggregated to national totals. All models were evaluated on aligned test targets using the same split structure. District-level forecasts were retained, and national-level forecasts were obtained by summing district predictions by target month. In the comparative analysis, SARIMAX was used as the baseline model.

#### SARIMAX.

SARIMAX is fitted district-wise, with one model per district, differencing in the specified order and seasonal order, no exogenous regressors, and ADF-based stationarity checks reported separately. The district-level SARIMAX model can be written as:


$$\begin{array}{c} \Phi_{p}(B)\Phi_{P}(B^{12})(1-B{)}^{d}(1-B^{12}{)}^{D}y_{t}=\Theta_{q}(B)\Theta_{Q}(B^{12})\varepsilon_{t}, \end{array}$$ (1)

where $y_{t}$ is the log-transformed dengue count at month *t*, *B* is the backshift operator, $\varepsilon_{t}$ is *t*he error term, and Φ(·) and Θ(·) denote the autoregressive and moving-average polynomials. In the baseline specification, *d* = 1, *D* = 1, and the seasonal period was fixed at 12 months.

#### Prophet.

Prophet is also fitted district-wise in a rolling-origin setup. It uses monthly forecasts, disables built-in yearly seasonality, and uses month-dummy regressors instead. The Prophet model can be written as:


$$\begin{array}{c} y_{t}=g(t)+\sum_{m=1}^{11}\beta_{m}M_{m,t}+\varepsilon_{t}, \end{array}$$ (2)

where $y_{t}$ is the log-transformed dengue count at month *t*, *g*(*t*) is the trend componen*t*, $M_{m,t}$ is *t*he month-indicator regressor for month *m*, $\beta_{m}$ is its coefficient, and $\varepsilon_{t}$ is the error term.

#### Machine-learning models.

MLR, SVR, RF, XGBoost, and CatBoost follow the same district-panel direct multi-horizon setup. They use the train/val/purge/test split already assigned in preprocessing, keep purge rows as source-context rows, and construct horizon-specific targets by shifting each source row to its future target date. RF, XGBoost, and CatBoost also add target-month sine and cosine features for direct forecasting. For the machine-learning models, the horizon-specific forecast is written as:


$$\begin{array}{c} {\hat{y}}_{t+h}^{\left(m\right)}=f_{h}^{\left(m\right)}({𝐱}_{t}), \end{array}$$ (3)

where m∈{MLR,SVR,RF,XGBoost,CatBoost}, *t* denotes the source month, *h* deno*t*es the forecast horizon, ${𝐱}_{t}$ denotes the predictor set available at month *t*, and ${\hat{y}}_{t+h}^{\left(m\right)}$ denotes the horizon-*h* prediction from model *m*.

#### LSTM-based models.

Stacked LSTM and attention-based LSTM also use a direct multi-horizon district-panel design. Both build source sequences ending at the source date and add target-month sine and cosine as a separate static branch. For the LSTM-based models, the horizon-specific forecast is written as:


$$\begin{array}{c} {\hat{y}}_{t+h}=f_{h}\;\left({𝐱}_{t-k+1:t},{𝐳}_{t+h}\right), \end{array}$$ (4)

where *k* denotes the look-back length, ${𝐱}_{t-k+1:t}$ denotes the input sequence from month t−k+1 to month *t*, and ${𝐳}_{t+h}$ denotes the horizon-specific target-month covariates.

#### Temporal fusion transformer (TFT).

TFT is the only joint multi-horizon model. It predicts all horizons up to six months ahead in one model, uses a 12-month encoder, treats target-month sine and cosine as known future covariates, excludes source-row Year, Month_sin, and Month_cos, and produces quantile forecasts. For TFT, the joint multi-horizon forecast is written as:


$$\begin{array}{c} {\hat{y}}_{t+1:t+6}=f\;\left({𝐱}_{t-k+1:t},\chi_{t+1:t+6}\right), \end{array}$$ (5)

where $\chi_{t+1:t+6}$ denotes the known future covariates over the prediction window.

### Sensitivity analysis

Sensitivity analysis was conducted to test whether the comparative results changed under controlled perturbations of the final model specifications. For the machine-learning, LSTM-based, and TFT models, sensitivity was implemented as one-factor-at-a-time ablation using the same preprocessed panel, the same train/val/purge/test split, and the same horizon definitions as the main experiments. Fixed main-model configurations were reused when available, and tuning was disabled during sensitivity runs. The baseline experiment retained the full feature set. Additional experiments removed one feature group at a time: climate variables, serotype variables, temporal variables, or population density. For MLR and SVR, we removed source-time temporal variables. For the stacked LSTM and attention-based LSTM models, we removed the target-time static temporal branch. For TFT, we removed the known future temporal covariates from the model schema.

Model-specific sensitivity settings were used for Prophet and SARIMAX. Prophet sensitivity kept the same grouped district-level rolling-origin design and compared the full model against a version without month-dummy regressors. SARIMAX was examined with specification changes through four experiments: the baseline configuration, the same orders without order search, a non-seasonal variant, and a simpler non-seasonal ARIMA specification.

### Evaluation protocol

All models were evaluated on the original count scale. Performance was summarized in three ways: (1) **Overall**, by pooling all aligned test predictions across districts and horizons; (2) **Per-horizon**, where metrics were computed separately for each forecast horizon h∈{1,...,6}; and (3) **Regime-wise**, where the test set was partitioned into normal and outbreak periods. District-level forecasts were retained for district-wise analysis, and national-level forecasts were obtained by summing district predictions by target month.

We report Root Mean Squared Error (RMSE), Mean Absolute Error (MAE), Mean Bias Error (MBE), normalized RMSE (NRMSE), and Mean Absolute Scaled Error (MASE). For a set of observed counts $c_{i}$ and predictions ${\hat{c}}_{i}$, MAE and RMSE are defined as


$$\begin{array}{cc} MAE & =\frac{1}{N}\sum_{i=1}^{N}|c_{i}-{\hat{c}}_{i}|, \end{array}$$ (6)

$$\begin{array}{cc} RMSE & =\sqrt{\frac{1}{N}\sum_{i=1}^{N}(c_{i}-{\hat{c}}_{i}{)}^{2}}, \end{array}$$ (7)

and MBE is defined as


$$\begin{array}{c} MBE=\frac{1}{N}\sum_{i=1}^{N}({\hat{c}}_{i}-c_{i}). \end{array}$$ (8)

The MASE denominator was computed from the pooled district-wise training series as the mean absolute first difference of dengue counts:


$$\begin{array}{c} MASE=\frac{\frac{1}{N}\sum_{i=1}^{N}|c_{i}-{\hat{c}}_{i}|}{\frac{1}{\sum_{d=1}^{D}(T_{d}-1)}\sum_{d=1}^{D}\sum_{t=2}^{T_{d}}|c_{d,t}-c_{d,t-1}|}, \end{array}$$ (9)

where $c_{d,t}$ denotes the dengue count for district *d* at month *t*, *D* is the number of districts, and $T_{d}$ is *t*he number of training months for district *d*.

For each model and forecast horizon, the outbreak threshold was defined as the 90th percentile of the training-set dengue counts on the count scale:


$$\begin{array}{c} \theta_{0.90}=Q_{0.90}\;\left(\{c_{d,t}:(d,t)\in {𝒯}_{train}\}\right). \end{array}$$ (10)

Threshold-dependent alert metrics such as precision, recall, and F1 should be interpreted as within-model operational alert performance rather than a common-threshold comparison as they were estimated within each model-specific evaluation. A test observation was classified as an outbreak when $c_{i}\geq \theta_{0.90}$. Precision, recall, F1-score, specificity, ROC-AUC, and PR-AUC were then computed from the count-scale forecasts.

### Statistical significance testing

Statistical significance of forecast differences was assessed using the Diebold–Mariano (DM) test with the Harvey-Leybourne-Newbold (HLN) small-sample correction. Prophet was used as the reference model, and pairwise comparisons were performed against Attention-LSTM, Stacked LSTM, XGBoost, and TFT. Two loss families were considered: squared error for RMSE-oriented comparisons and absolute error for MAE-oriented comparisons.

For a given horizon *h*, let $\ell_{d,t,h}^{\left(r\right)}$ and $\ell_{d,t,h}^{\left(c\right)}$ denote the district-level losses of the reference and competitor models, respectively, for district *d* and TargetDate *t*. The TargetDate-level mean loss differential was defined as


$$\begin{array}{c} {\bar{d}}_{t,h}=\frac{1}{D_{t}}\sum_{d=1}^{D_{t}}\left(\ell_{d,t,h}^{\left(c\right)}-\ell_{d,t,h}^{\left(r\right)}\right), \end{array}$$ (11)

where $D_{t}$ is the number of districts available at TargetDate *t*. Positive values of ${\bar{d}}_{t,h}$ indicate lower loss for the reference model. The HLN-correc*t*ed DM statistic was then computed from the series $\{{\bar{d}}_{t,h}{\}}_{t=1}^{T_{h}}$, with Newey-West lag set to h−1


$$\begin{array}{c} DM_{h}^{*}=\frac{{\bar{d}}_{h}}{\sqrt{{\hat{LRV}}_{h}/T_{h}}}\sqrt{\frac{T_{h}+1-2h+h(h-1)/T_{h}}{T_{h}}}, \end{array}$$ (12)

where ${\bar{d}}_{h}$ is the sample mean of the loss differential series, $T_{h}$ is the number of TargetDate-level observations for horizon *h*, and ${\hat{LRV}}_{h}$ is the Newey–West estimate of its long-run variance.

## Results and analysis

The ADF results show that the log-transformed dengue series remained non-stationary in 7 of the 11 districts while all first-differenced series were stationary at α=0.05 (Table 2). Stationarity at the level was observed only in Faridpur, Jessore, Khulna, and Mymensingh. These results rationalize the differencing used in the SARIMAX baseline.

**Table 2:** Augmented Dickey–Fuller (ADF) test results for the log-transformed and first-differenced dengue series by district.

District	Log-transformed $y_{t}$		First-differenced $\Delta y_{t}$	
	ADF statistic	*p*-value	ADF statistic	*p*-value
Barishal	−1.641	0.462	−3.231	0.018
Bhola	−2.668	0.080	−6.115	<0.001
Chattogram	−1.679	0.442	−7.783	<0.001
Cox’s Bazar	−2.297	0.173	−9.276	<0.001
Dhaka	−2.411	0.139	−7.739	<0.001
Faridpur	−3.049	0.031	−6.887	<0.001
Jessore	−3.485	0.008	−4.171	<0.001
Khulna	−3.209	0.019	−7.679	<0.001
Mymensingh	−2.997	0.035	−7.532	<0.001
Rajshahi	−2.860	0.050	−5.786	<0.001
Sylhet	−2.781	0.061	−8.636	<0.001

A series was considered stationary at α=0.05 when *p* < 0.05. All first-differenced series were stationary. The log-transformed series remained non-stationary in Barishal, Bhola, Chattogram, Cox’s Bazar, Dhaka, Rajshahi, and Sylhet. Rajshahi was classified from the original unrounded output; its displayed *p*-value rounds to 0.050.

Across the full test set, Prophet achieved the best overall count-scale performance (Table 3), with the lowest RMSE (3974.869), MAE (1241.106), MASE (26.450), and NRMSE (0.134). MLR and Attention-LSTM formed the next performance tier, with nearly identical RMSE values of 4083.392 and 4083.548, respectively. Stacked LSTM was close, with an RMSE of 4099.284. SVR, RF, XGBoost, CatBoost, and TFT showed similar performance, with RMSE values around 4110 and MAE values around 1286–1287. SARIMAX performed worst overall, with an RMSE of 6501.069 and an MAE of 1621.359.

**Table 3:** Overall test-set performance of the forecasting models on the count scale.

Model	RMSE	MAE	MBE	MASE	NRMSE
SARIMAX	6501.069	1621.359	49.514	34.554	0.220
Prophet	3974.869	1241.106	−1240.216	26.450	0.134
MLR	4083.392	1267.447	−1265.133	27.011	0.138
SVR	4112.006	1287.430	−1287.425	27.437	0.139
RF	4110.775	1286.540	−1286.454	27.418	0.139
XGBoost	4110.034	1286.183	−1286.119	27.411	0.139
CatBoost	4110.128	1286.246	−1286.186	27.412	0.139
Stacked LSTM	4099.284	1270.471	−1269.314	27.076	0.139
Attention-LSTM	4083.548	1275.100	−1274.898	27.174	0.138
TFT	4111.035	1286.682	−1286.631	27.421	0.139

Bias patterns further separated the models. Prophet and all machine-learning and deep-learning models showed large negative MBE values, indicating underprediction. SARIMAX was the only model with near-zero mean bias (MBE = 49.514) but it didn’t sustain at boarder predictive accuracy. Prophet balanced overall error reduction. Remaining non-classical models delivered similar performance.

## Performance across forecast horizons

The forecasting panel contained 891 district-month observations from April 2017 to December 2023 after lag correction and removal of imputed records. The test window spanned September 2022 to December 2023 and included the late-2022 outbreak and the 2023 outbreak. National monthly dengue counts increased from 8926 in September 2022–18314 in October 2022, and from 980 in May 2023–42960 in August 2023. The months from July to October 2023 accounted for 65.2% of all test-period cases. Dhaka contributed 65.1% of all test-set cases, including 75.5% in October 2022 and 60.4% in September 2023.

**Fig 5:**
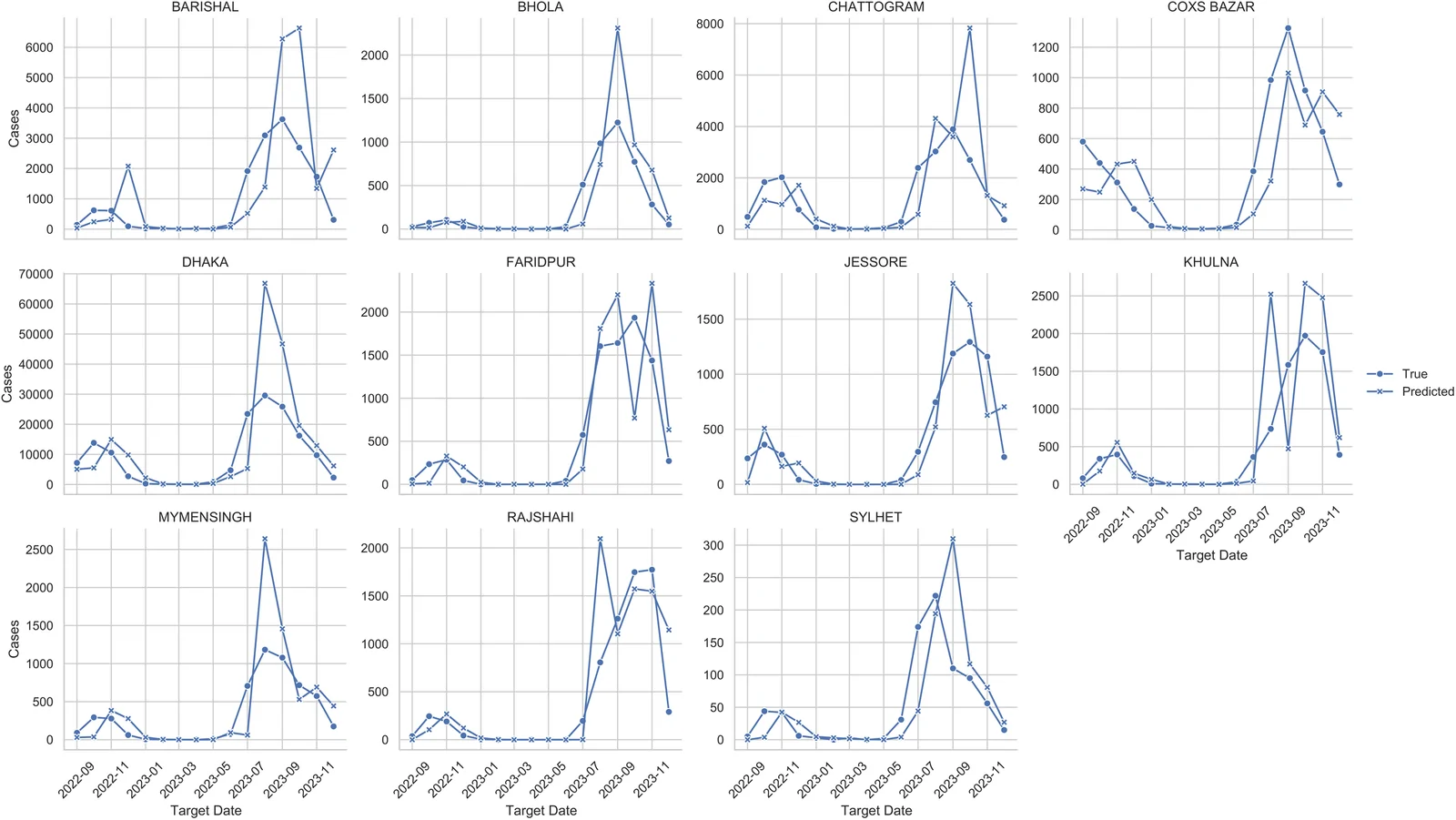
One-step-ahead district-level SARIMAX forecasts during the test window. The figure shows observed and predicted dengue counts for horizon 1 across study districts.

The horizon-wise results show a clear separation between short-range and medium-range forecasting. At *h* = 1, SARIMAX achieved the lowest error, with an RMSE of 3716.948 and an MAE of 952.653. This indicates that district-specific autoregressive structure remained useful for immediate one-step prediction. Prophet was the best model from *h* = 2 to *h* = 6. Its RMSE remained within 3952.613–4004.231 whereas all other non-SARIMAX models remained above this range at the corresponding horizons. MLR, attention-LSTM, and stacked LSTM formed the next performance tier. Among them, MLR was stable across horizons. Attention-LSTM was competitive at shorter lead times. Stacked LSTM showed a local improvement at *h* = 4 though this was not maintained at *h* = 5 and *h* = 6. XGBoost, CatBoost, RF, TFT, and SVR formed a compressed group with quite flat RMSE and MAE curves across all horizons. Table 4 strengthens this evidence. When the test set was partitioned into normal and outbreak regimes and errors were averaged within those subsets, MLR achieved the lowest RMSE and MAE in both regimes with Prophet ranking second. The regime-wise table averages errors within subsets and then across horizons. The pooled overall metrics are computed over all test targets. The district-wise RMSE results in Table 5 illustrates the domination of large districts and high-incidence months. Prophet was the best model in Dhaka and Chattogram, the two districts that together contributed 73.6% of all test-period cases. Stacked LSTM was best in Barishal and Mymensingh; MLR was best in Bhola, Rajshahi, and Sylhet; SARIMAX was best in Cox’s Bazar, Faridpur, Jessore, and Khulna. Although SARIMAX achieved the lowest RMSE in more districts than Prophet, its wins basically at smaller districts (shown in Fig 5). But its error remained large in Dhaka and Barishal.

**Table 4:** Regime-wise test-set errors averaged across forecast horizons.

Model	Normal		Outbreak	
	RMSE	MAE	RMSE	MAE
SARIMAX	1059.746	274.524	8990.886	3092.647
Prophet	39.465	24.531	5528.155	2377.998
MLR	24.526	16.210	5449.251	2244.579
SVR	31.291	20.234	5487.397	2277.002
RF	30.597	19.811	5485.755	2275.743
XGBoost	30.427	19.741	5484.770	2275.164
CatBoost	30.515	19.780	5484.894	2275.247
Stacked LSTM	33.907	22.057	5619.942	2368.805
Attention-LSTM	37.237	24.039	5598.570	2376.138
TFT	42.988	27.459	5717.441	2463.239

Values are averaged across test-set horizons *h* = 1–6. Normal and outbreak regimes were defined using the model-specific outbreak threshold.

**Table 5:** District-wise test RMSE of the forecasting models.

District	SARIMAX	Prophet	MLR	SVR	RF	XGB	CatB	S-LSTM	A-LSTM	TFT
Barishal	2062.655	1515.349	1516.227	1531.379	1530.973	1530.781	1530.570	1495.465	1517.552	1531.266
Bhola	503.295	458.075	451.796	462.924	462.679	462.691	462.553	457.990	462.249	462.910
Chattogram	2346.390	1692.249	1707.932	1725.821	1725.379	1725.246	1725.002	1717.311	1714.062	1725.750
Cox’s Bazar	477.722	532.823	528.459	551.729	551.401	551.459	551.289	531.972	547.698	551.657
Dhaka	21274.501	12871.376	13240.265	13327.455	13323.496	13321.060	13321.464	13295.844	13236.008	13324.192
Faridpur	671.664	844.484	833.956	852.166	851.711	851.514	851.505	838.775	849.551	852.081
Jessore	455.124	569.261	566.654	580.752	580.455	580.223	580.145	570.047	570.960	580.748
Khulna	722.235	803.382	796.960	813.775	813.410	813.225	813.120	780.078	807.123	813.765
Mymensingh	566.954	495.105	492.624	507.403	507.022	506.824	506.781	476.924	493.956	507.370
Rajshahi	739.485	731.509	715.128	735.674	735.465	735.445	735.344	719.228	729.962	735.715
Sylhet	93.645	80.704	72.220	82.444	82.246	82.317	82.297	77.061	80.557	82.469

Lower RMSE indicates better predictive accuracy.

Fig 10, Tables 4, and 5 show that model ranking depended on the evaluation perspective. SARIMAX performed well in one-step forecasting. MLR was strong candidate when errors were averaged within normal and outbreak subsets. Prophet outperforms others in aggregate comparison.

### Sensitivity and uncertainty analysis

**Fig 6:**
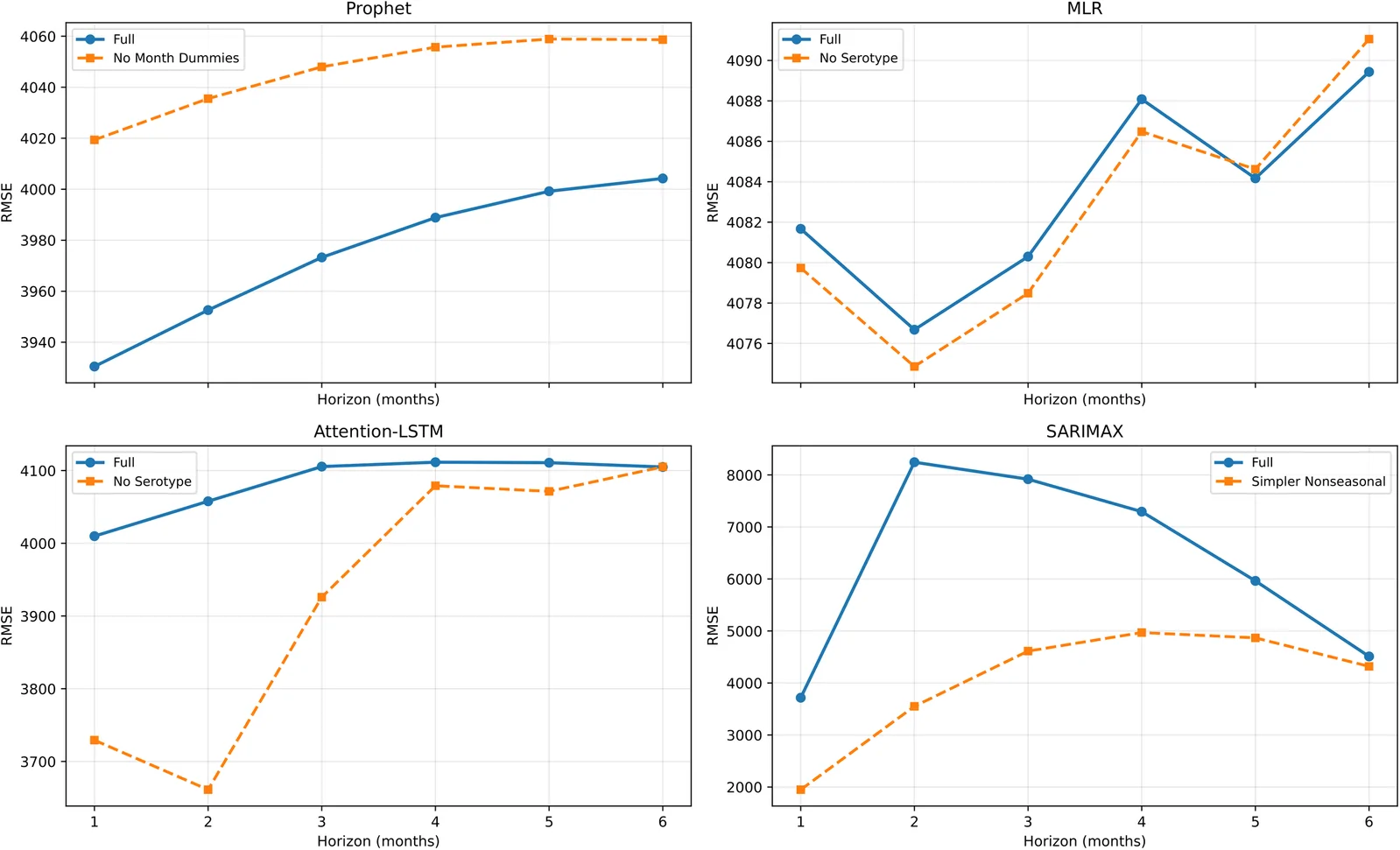
Forecast-horizon RMSE sensitivity analysis for the selected models. Each panel compares the full specification with one alternative. Prophet: no month dummies. MLR: no serotype. Attention-LSTM: no serotype. SARIMAX: simpler non-seasonal. Top-left: Prophet, full vs. no month dummies. Top-right: MLR, full vs. no serotype. Bottom-left: Attention-LSTM, full vs. no serotype. Bottom-right: SARIMAX, full vs. simpler non-seasonal. No month dummies removes the month-indicator regressors. No serotype removes the serotype predictors. Simpler non-seasonal removes the seasonal ARIMA terms.

Sensitivity analysis yielded different patterns across the four selected models (Tables 6 and 7, Fig 6). For Prophet, the full specification remained the best-performing variant. We examined with the removal of month-dummy regressors that increased RMSE from 3974.869 to 4046.053 and MAE from 1241.106 to 1260.170. The same pattern was observed across forecast horizons.

**Table 6:** Overall sensitivity comparison for the selected models.

Model	Alternative	$RMSE_{full}$	$RMSE_{alt}$	ΔRMSE	MAE	Alt. MAE	ΔMAE
Prophet	No month dummies	3974.869	4046.053	71.184	1241.106	1260.170	19.064
MLR	No serotype	4083.392	4082.544	−0.849	1267.447	1266.926	−0.521
Attention-LSTM	No serotype	4083.548	3932.587	−150.961	1275.100	1189.481	−85.619
SARIMAX	Simpler non-seasonal	6501.069	4178.289	−2322.781	1621.359	1424.630	−196.729

Full denotes the retained main specification. Alt. denotes alternative specification. Δ is calculated as alternative minus full. Positive values indicate worse performance and negative values indicate improved performance.

**Table 7:** Best-performing overall sensitivity specification by model.

Model	Best RMSE spec.	RMSE	Best MAE spec.	MAE
Prophet	Full	3974.869	Full	1241.106
MLR	No serotype	4082.544	No serotype	1266.926
Attention-LSTM	No serotype	3932.587	No serotype	1189.481
SARIMAX	Simpler non-seasonal	4178.289	Simpler non-seasonal	1424.630

No month dummies removes the month-indicator regressors. No serotype removes the serotype predictors. Simpler non-seasonal removes the seasonal ARIMA terms.

Sensitivity effects were limited for MLR. Removing serotype predictors gives the best variant though the reduction was negligible. In contrast, removal of temporal predictors produced higher RMSE(+23.670) and MAE(+16.008) in MLR performance. These results point out the larger effect of temporal predictors than the serotype feature group in MLR. Sensitivity effects were larger for attention-LSTM. When we removed serotype predictors, it reduced RMSE from 4083.548 to 3932.587 and MAE from 1275.100 to 1189.481. This improvement was also reflected across horizons. Removing population density also improved performance, although less than serotype removal. On the other hand, removal of climate or temporal inputs did not improve error metrics, referring to the sensitivity to predictor composition in case of this architecture.

For SARIMAX, removal of seasonal ARIMA terms outperformed the baseline seasonal SARIMAX model, reducing overall RMSE from 6501.069 to 4178.289 and MAE from 1621.359 to 1424.630. This simpler variant achieved the lowest RMSE at every forecast horizon and the lowest MAE at horizons 1–5.

These sensitivity findings suggest that predictor contribution was architecture-dependent. Serotype removal improved MLR and attention-LSTM, referring serotype covariates did not provide uniform forecasting gains across models. Prophet retained full specification. MLR showed minor sensitivity to specification changes. In contrast, attention-LSTM and SARIMAX achieved lower errors under simplified alternatives. The better performance of the simpler non-seasonal SARIMAX specification also suggests that fixed seasonal ARIMA terms may be restrictive when outbreak timing shifts across years.

We also evaluated uncertainty for TFT using 80% interval coverage, mean interval width, and quantile pinball loss because of its probabilistic nature. We reported it for both Normal and Outbreak regimes across forecast horizons (Table 8). Coverage was considerably below nominal levels at all horizons and dropped to zero for Outbreak periods. It refers to poor interval calibration during epidemic surges despite wider prediction intervals.

**Table 8:** TFT regime-wise 80% interval coverage, mean interval width, and pinball loss by forecast horizon.

Hor.	Normal					Outbreak				
	Cov80	Width80	PB_0.10_	PB_0.50_	PB_0.90_	Cov80	Width80	PB_0.10_	PB_0.50_	PB_0.90_
1	0.238	1.268	2.673	13.275	23.147	0.000	1.779	243.837	1218.994	2192.929
2	0.226	1.542	2.666	13.177	22.866	0.000	1.957	243.825	1218.861	2192.661
3	0.179	1.989	2.661	13.045	22.438	0.000	2.143	243.816	1218.772	2192.418
4	0.188	2.188	2.791	13.645	23.430	0.000	2.238	246.340	1231.370	2215.043
5	0.174	2.290	2.924	14.286	24.520	0.000	2.214	248.919	1244.273	2238.279
6	0.172	2.303	3.056	14.948	25.695	0.000	2.203	251.554	1257.447	2262.005

Cov80 denotes the proportion of observations falling within the predicted 80% interval. Width80 denotes the mean width of the 80% prediction interval on the count scale. PB_q_ denotes the pinball loss at quantile *q*.

The zero outbreak-period coverage indicates that TFT produced 80% prediction intervals, but none of the observed outbreak counts fell within those intervals. This suggests that TFT learned a low count predictive distribution from normal-period training. But during 2022−23 outbreak, it’s predicted upper quantile remained below the observed counts at every horizon which resulted in not falling inside the 10th–90th percentile interval, producing zero 80% interval coverage.

### Practical implications: From magnitude failure to actionable alerts

The outbreak-classification results showed that at one-step-ahead (*h* = 1), SARIMAX achieved the best outbreak-detection performance, with the precision (0.886), recall (0.824), F1 score (0.854), and ROC-AUC (0.950) among all models (Table 9). This contrasts with the aggregate count-scale results where SARIMAX performed worst. Prophet, MLR, and attention-LSTM showed a different profile. All three achieved high precision but low recall at *h* = 1. Prophet achieved precision of 0.923 with recall of 0.130, MLR achieved precision of 1.000 with recall of 0.100, and attention-LSTM achieved precision of 1.000 with recall of 0.124. These results indicate conservative alerting: the alerts were usually correct, but many outbreak months were missed. The high ROC-AUC values suggest useful outbreak ranking, but the classification threshold led to low sensitivity. This model-specific thresholding improves within-model calibration for outbreak alerting. But it also means that cross-model comparisons of precision, recall, and F1 are less direct than threshold-independent metrics.

**Table 9:** One-step-ahead outbreak-classification performance on the test set.

Model	Threshold	Precision	Recall	F1	ROC-AUC
SARIMAX	196.6	0.886	0.824	0.854	0.950
Prophet	124.2	0.923	0.130	0.229	0.871
MLR	80.0	1.000	0.100	0.182	0.905
SVR	80.0	0.000	0.000	0.000	0.232
RF	80.0	0.000	0.000	0.000	0.558
XGBoost	80.0	0.000	0.000	0.000	0.695
CatBoost	80.0	0.000	0.000	0.000	0.727
Stacked LSTM	94.0	1.000	0.041	0.079	0.836
Attention-LSTM	94.0	1.000	0.124	0.220	0.842
TFT	124.2	0.000	0.000	0.000	0.598

Metrics are reported for one-step-ahead test forecasts (*h* = 1). The outbreak threshold is model-specific and was defined as the 90th percentile of the training-set dengue counts on the count scale.

SVR, RF, XGBoost, CatBoost, and TFT all produced zero recall and zero F1 at *h* = 1. Hence, they failed as thresholded outbreak detectors under the decision rule. Some still had moderate ROC-AUC and PR-AUC. This indicates they ranked outbreak risk partially but failed to produce alerts. Across horizons, SARIMAX kept the highest recall and F1, though both declined with longer lead times. Prophet and MLR had high ROC-AUC and PR-AUC but low recall while the two LSTM variants showed unstable alerting performance. These results show the operational trade-off. Prophet was the strongest on pooled count-scale forecasting, whereas SARIMAX was the strongest on outbreak alerting.

### Pairwise significance testing

The result of pairwise significance testing (Table 10) of shortlisted candidates shows limited statistical separation. Under squared-error loss, none of the pairwise comparisons were significant at any horizon after Holm adjustment. Under absolute-error loss, significance was observed only at *h* = 1, where Prophet differed from attention-LSTM, stacked LSTM, XGBoost, and TFT (*p* = 0.018 in all four comparisons). No significant differences were detected at horizons 2–6 which indicates that Prophet’s advantage was modest, with statistically significant separation for one-step absolute-error comparisons.

**Table 10:** Pairwise Diebold–Mariano significance testing comparing Prophet with shortlisted competing models across horizons.

Panel A. Squared-error loss					Panel B. Absolute-error loss			
Horizon	vs ATT-LSTM	vs S-LSTM	vs XGB	vs TFT	vs ATT-LSTM	vs S-LSTM	vs XGB	vs TFT
H1	0.123	0.123	0.123	0.123	**0.018**	**0.018**	**0.018**	**0.018**
H2	0.694	0.694	0.694	0.694	0.275	0.327	0.275	0.275
H3	1.000	1.000	1.000	1.000	0.619	0.619	0.619	0.619
H4	1.000	1.000	1.000	1.000	0.879	0.957	0.879	0.879
H5	1.000	1.000	1.000	1.000	0.999	0.999	0.999	0.999
H6	1.000	1.000	1.000	1.000	1.000	1.000	1.000	1.000

Entries are Holm-adjusted *p*-values; bold indicates *p* < 0.05. Tests were performed on aligned common test rows using count-scale forecasts. Losses were averaged across districts within each TargetDate. Reported values are Holm-adjusted *p*-values from the Harvey–Leybourne–Newbold corrected Diebold–Mariano test.

These results support a cautious interpretation of Prophet’s lead. Prophet had the lowest pooled RMSE and MAE at most horizons, but its advantage was modest and usually not statistically significant. Only the (*h* = 1) absolute-error comparison remained significant after correction. Thus, Prophet was the strongest aggregate model. But it was separable from the shortlisted competitors for one-step-ahead MAE.

### Model interpretability and feature importance

Interpretability analysis showed both agreement and separation across the linear, tree-based, and sequence-model families. These interpretability results describe model reliance and predictive contribution, not causal effects of the covariates on dengue incidence. Across models, three predictor groups recurred: serotype history, seasonal timing, and delayed climatic conditions. However, the relative importance of these groups differed by model class and by forecast horizon.

For MLR, the aggregate coefficient summary indicates that the dominant linear contributors across horizons were population density, seasonal phase, sunshine hours, and calendar year. Table 11 shows that population density had the largest mean absolute standardized coefficient, followed by Month (Cos), Sunshine Hrs (Lag 1), Year, and Month (Sin). At horizon 1, the strongest statistically supported effects were Year, Month (Sin), Month (Cos), population density, rainfall, sunshine, and average temperature, whereas DENV-1 (Lag 1) was not significant. By horizon 6, the linear structure shifted: population density, sunshine, Year, wind direction, and DENV-1 (Lag 1) remained significant. But seasonal sine and cosine terms were no longer significant.

**Table 11:** MLR coefficient summary across horizons.

Predictor	Mean $\left|\beta_{std}\right|$	H1 coeff.	H1 *p*-value	H6 coeff.	H6 *p*-value
Pop. Density	0.226	0.000159	<0.001	0.000160	<0.001
Month (Cos)	0.215	−0.626096	<0.001	0.135295	0.272
Sunshine Hrs (Lag 1)	0.199	0.157607	0.010	0.214010	0.002
Year	0.192	0.332039	<0.001	0.341465	<0.001
Month (Sin)	0.177	−0.943888	<0.001	0.220888	0.359
Avg. Temp (Lag 3)	0.105	0.083761	0.025	0.005674	0.915
Humidity (Lag 1)	0.105	−0.013750	0.504	−0.042919	0.110
Rainfall (Lag 2)	0.099	−0.001390	0.004	0.000136	0.622
Wind Dir. (ENE)	0.052	1.459103	0.662	−1.214692	0.038
DENV-1 (Lag 1)	0.048	−0.006395	0.160	0.020045	0.002

Mean $\left|\beta_{std}\right|$ denotes the mean absolute standardized coefficient aggregated across forecast horizons. H1 and H6 report robust coefficient estimates from the horizon-specific MLR models for the shortest and longest forecast horizons, respectively.

**Fig 7:**
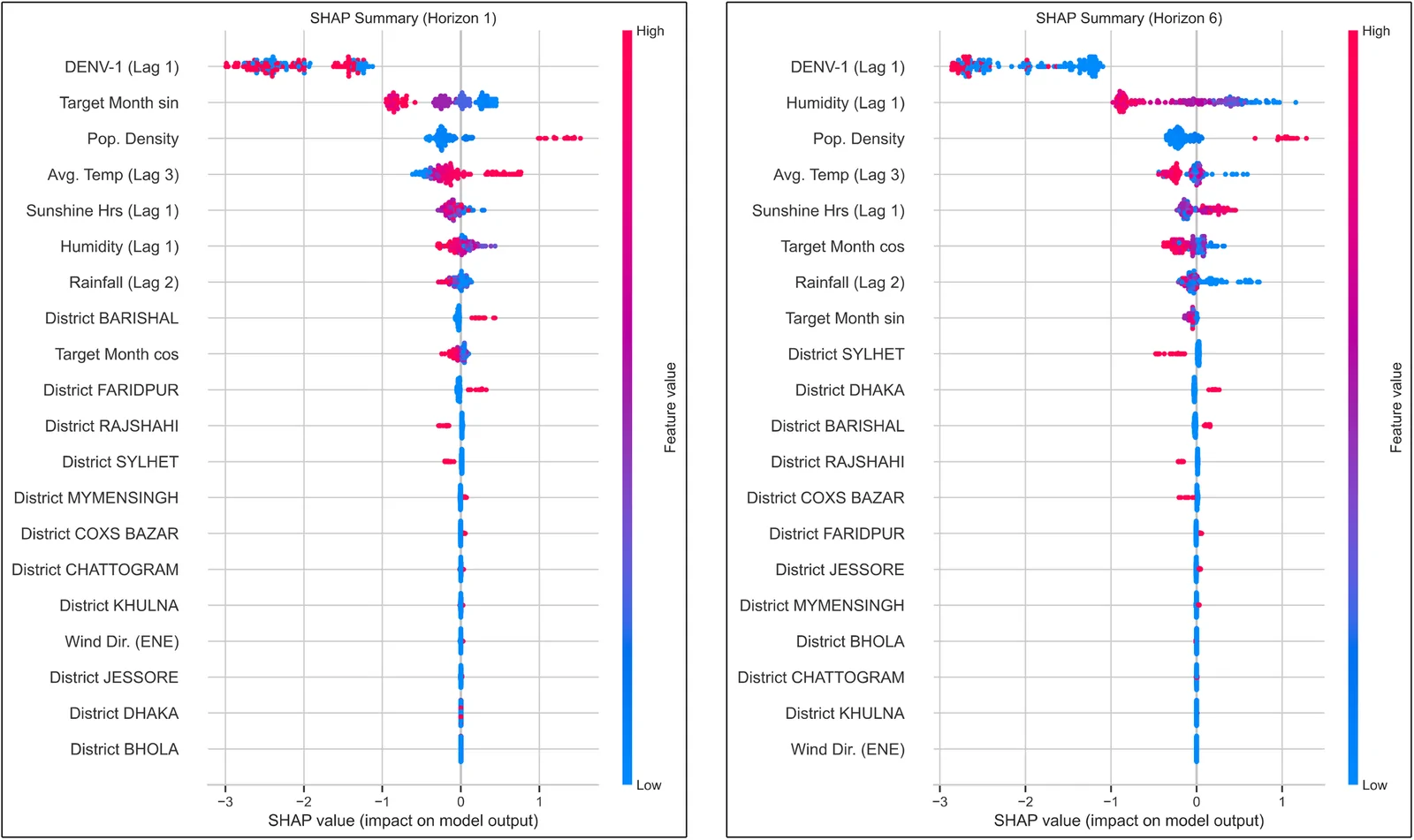
XGBoost SHAP summaries for short- and long-horizon forecasts. SHAP beeswarm plots for horizon 1 (left) and horizon 6 (right). Features are ordered by mean absolute SHAP value within each horizon. Color indicates feature value, and horizontal position indicates the direction and magnitude of contribution to the prediction on the log scale. Left: horizon 1. Right: horizon 6.

**Fig 8:**
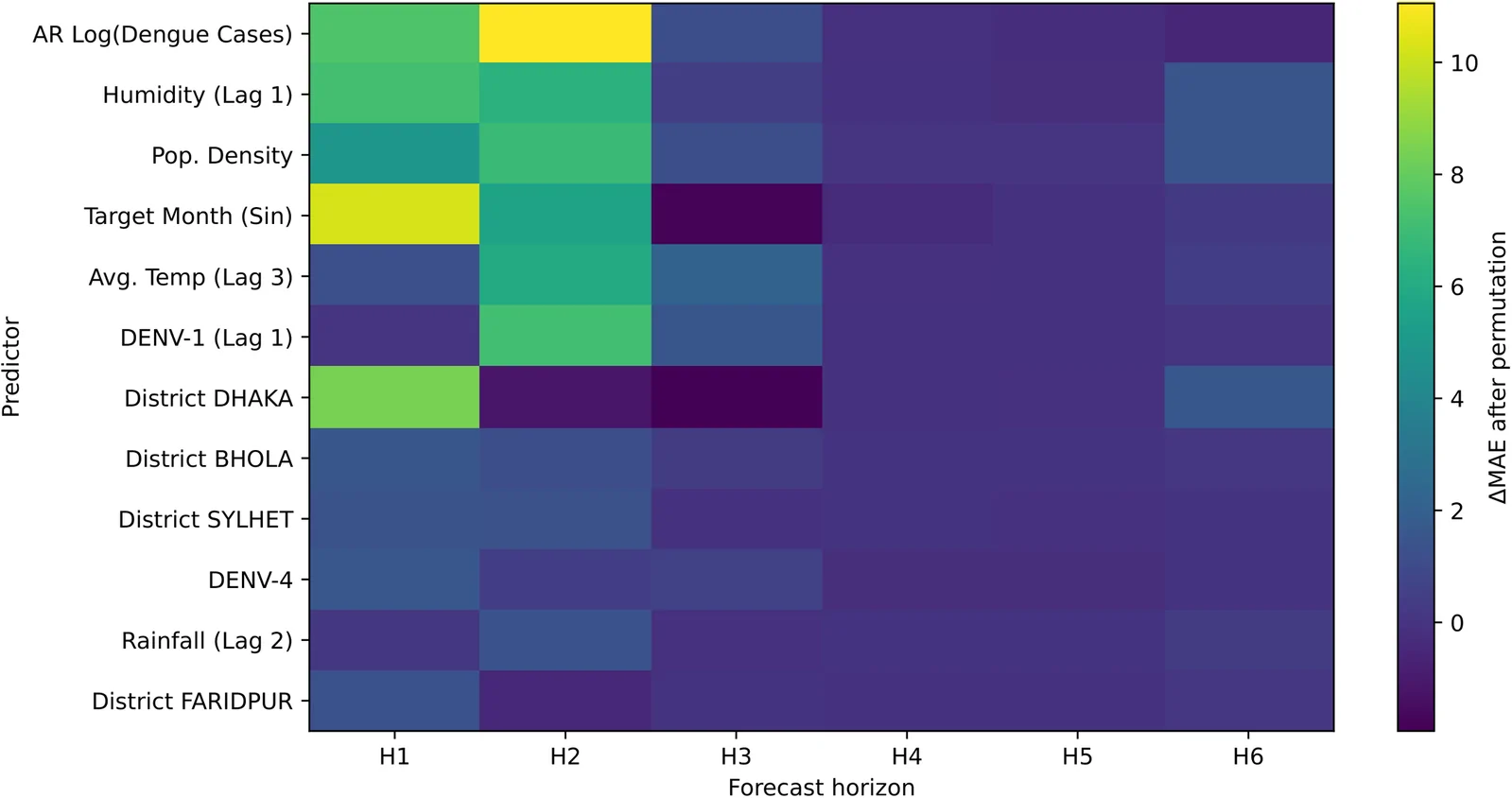
Attention-LSTM permutation importance by forecast horizon. Heatmap of the increase in test MAE after predictor permutation. Larger positive values indicate greater reliance of the model on the corresponding predictor at that horizon.

For XGBoost, SHAP (Fig 7) showed a different feature order. DENV-1 (Lag 1) was the top predictor at both horizons. At *h* = 1, Target Month (Sin), population density, and Avg. Temp (Lag 3) followed. At *h* = 6, humidity was second in order, with population density, temperature, sunshine, rainfall, and Target Month (Cos) also contributed. Thus, XGBoost depended on recent serotype information at both horizons. Though it later shifted from seasonal timing at *h* = 1 to climatic and environmental factor at *h* = 6. For attention-LSTM, permutation importance (Fig 8) was highest for AR Log(Dengue Cases), Humidity (Lag 1), population density, Target Month (Sin), Avg. Temp (Lag 3), and DENV-1 (Lag 1). The maximum impacts surfaced at *h* = 1 and *h* = 2 for AR Log(Dengue Cases) and Target Month (Sin). From *h* = 3 and beyond these impacts approached near zero, or negative which concludes that attention-LSTM relied on short-term autoregressive memory and contextual covariates with weaker feature dependence at longer horizons.

The interpretability analysis exhibits a common predictor group but the classical and non-classical algorithms behave in different manner while interacting with them. It can be noted that DENV-1 (Lag 1) was often stable and non-linear predictor, especially in XGBoost, and also appeared in MLR at longer horizons. Population density was important across all analyses. MLR and short-horizon XGBoost emphasized seasonal timing, while humidity and average temperature became important at longer horizons. Attention-LSTM differed by relying on autoregressive history than on external covariates.

### Causal inference analysis

The causal analysis showed different results for pooled linear dependence and district-specific conditional dependence. In the pooled Granger analysis, Avg. Temp (Lag 3) was a climatic precursor with a strong lag of 2 months beyond the original 3-month offset, giving a total lag of 5 months. The Fisher summary on Table 12 showed that this temperature signal remained significant across total lags of 5–9 months. Rainfall showed weaker pooled linear evidence. District-level Granger results showed moderate spatial consistency for temperature. Avg. Temp (Lag 3) was significant in Barishal, Bhola, Cox’s Bazar, Faridpur, and Mymensingh, while Dhaka and Khulna were near the threshold. Its main total lag was 5 months, with some districts shifting to 6 months and one to 8 months. Rainfall was more variable. No district was significant for Rainfall (Lag 2), and its strongest total lags ranged from 3 to 8 months. Thus, temperature gave a more reproducible pooled linear signal than rainfall.

**Table 12:** Combined summary of pooled Granger causality and PCMCI results for selected climatic predictors of dengue incidence.

Granger causality				PCMCI			
Predictor	Lag	Total lag	*p*-value	Predictor	Lag	Total lag	*p*-value
Avg. Temp (Lag 3)	2 Mo.	5 Mo.	<0.001	Avg. Temp (Lag 3)	5 Mo.	8 Mo.	0.151
Rainfall (Lag 2)	6 Mo.	8 Mo.	0.160	Rainfall (Lag 2)	5 Mo.	7 Mo.	0.018

Lag denotes the strongest method-specific lag. Total lag denotes the original predictor offset plus the detected lag. Pooled Granger results were obtained from Fisher-combined district-level tests by lag.

**Fig 9:**
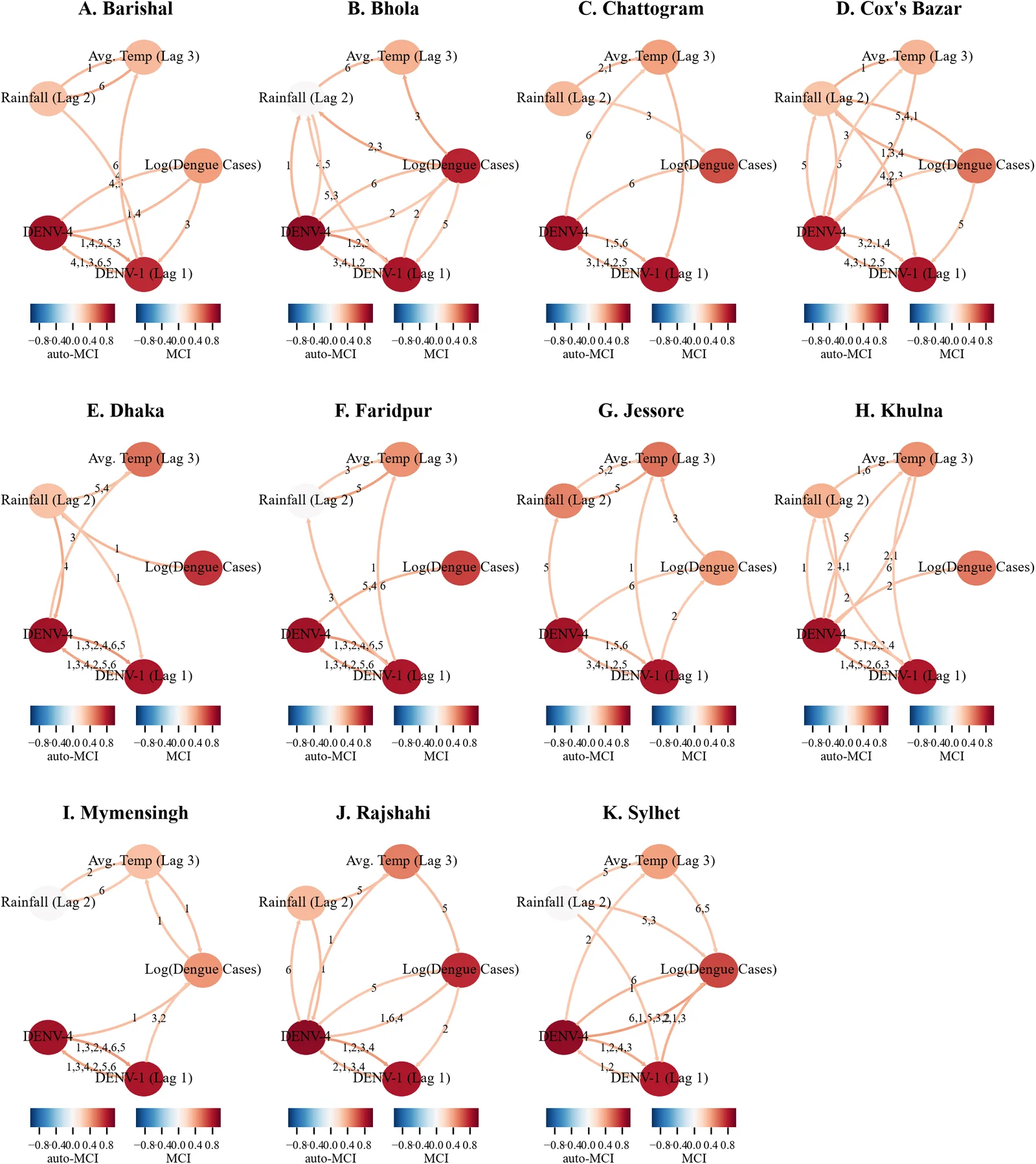
PCMCI analysis. District-wise causal graphs generated by PCMCI using the GPDC test, showing significant non-linear links among dengue incidence, climatic covariates, and serotype variables. Panels A–K correspond to the 11 study districts. Edge labels indicate the strongest detected lag, while color intensity reflects the effect value. A. Barishal B. Bhola C. Chattogram D. Cox’s Bazar E. Dhaka F. Faridpur G. Jessore H. Khulna I. Mymensingh J. Rajshahi K. Sylhet. Lag denotes the strongest method-specific lag. Total lag denotes the original predictor offset plus the detected lag.

The PCMCI results shifted the interpretation toward a nonlinear network. The district graphs (shown in Fig 9) were influenced by links among DENV-4, DENV-1, rainfall, and temperature, while direct climatic links to dengue incidence were less stable. The main repeated pattern was coupling between DENV-4 and DENV-1 across lags. Climate variables were also linked with serotype nodes and with each other, especially rainfall and temperature. Direct links involving Log(Dengue Cases) varied by district. Thus, the climatic signal did not follow one common causal pathway.

The two methods propose a layered interpretation. Granger identified Avg. Temp (Lag 3) as stable pooled linear precursor, with a total delay of about 5 months. PCMCI showed an intricate pattern at district-level analysis. It concentrated on a coupled serotype-climate system rather than a straight climate-to-dengue link. Climate acts earlier, while serotypes are deeply associated with dengue cases. This difference explains the discrepancy of causal relevance and predictive usefulness. Serotype nodes occupied central positions in the PCMCI graphs but sensitivity analysis (Tables 6 and 7; Fig 6) showed that serotype predictors did not improve the RMSE and MAE of every model. Thus, dependency-network centrality indicates statistical association within the fitted causal-discovery framework, not guaranteed forecasting improvement.

## Discussion

This study shows that dengue forecasting in Bangladesh depends on forecast horizon and objective, not on a single best model. The pooled district-panel analysis did not identify a universal optimal model. Model ranking changed with horizon, epidemic regime, district contribution and whether the task was magnitude forecasting or alerting. Therefore, no model was optimal across 1–6 month horizons or across all evaluations.

**Fig 10:**
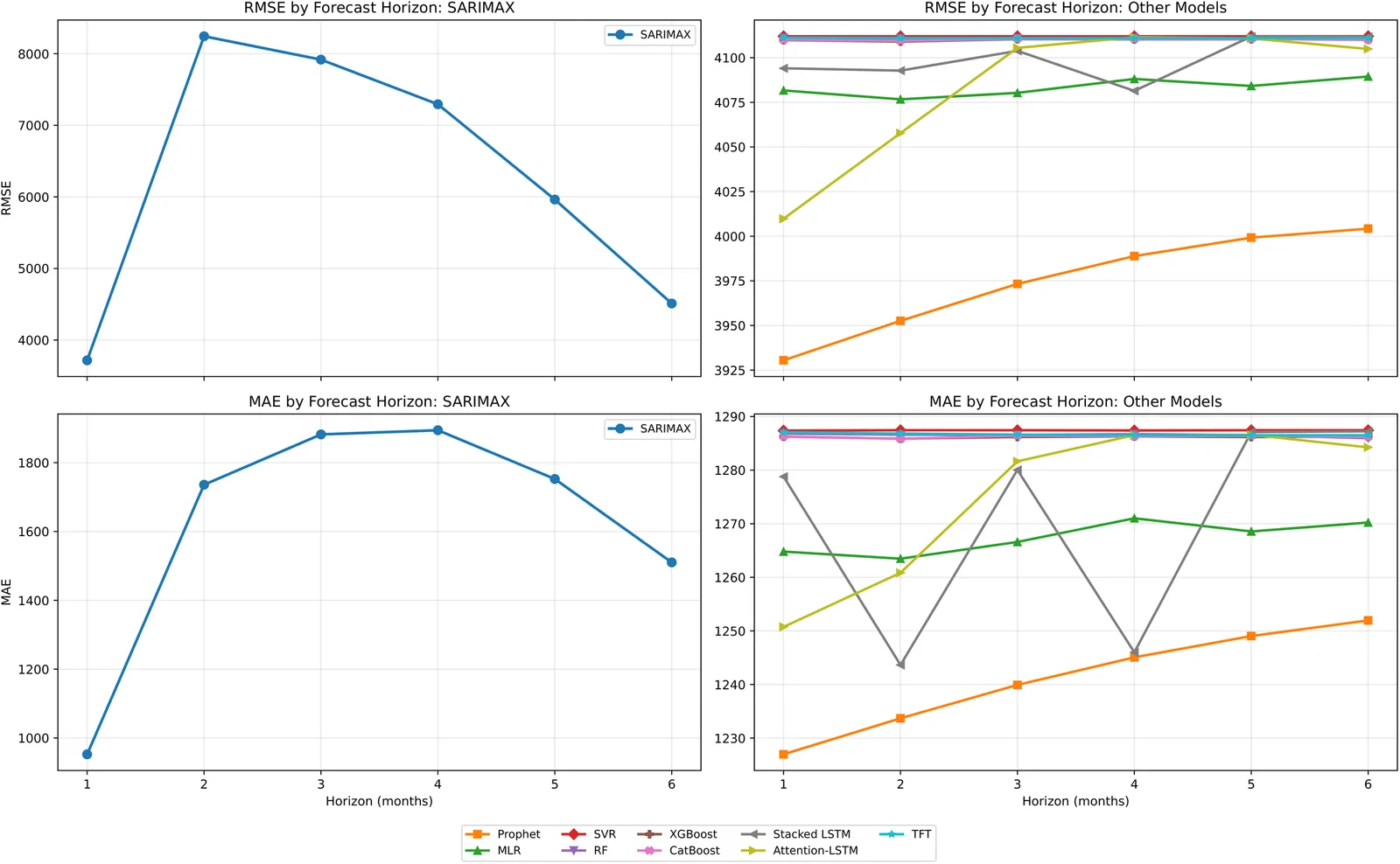
Forecast-horizon performance of the forecasting models on the count scale. The split-panel layout separates SARIMAX from the remaining models to preserve readability. Top-left: RMSE for SARIMAX. Top-right: RMSE for the remaining models. Bottom-left: MAE for SARIMAX. Bottom-right: MAE for the remaining models.

The results showed a tactical-versus-strategic behavior (Fig 10) of the forecasting algorithms after experimenting with the combination of classical and several state-of-the-art machine learning and deep learning algorithms. SARIMAX performed well in short term forecasting (specifically at horizon 1), district-level timing and one-step outbreak detection but its performance deteriorated with the increasing forecast horizon. Prophet performed best at horizons 2–6 from pooled aggregate performance perspective. This reflects model structure: SARIMAX captures short-term dynamics whereas Prophet provides more stable medium-range forecasts. Prophet provides stable medium-range forecasts (Table 10), although its advantage over competing models was statistically significant mainly for one-step absolute-error loss [22]. Regime-wise and district-wise analyses (Tables 4 and 5) help explain why Prophet was strong in the pooled national comparison. Pooled count-scale errors were controlled by high-burden districts and high-incidence months. Prophet performed best in high-burden districts and high-incidence months, while SARIMAX won more districts, mainly in lower-burden settings. However, this advantage should not be overinterpreted, as pairwise testing showed limited statistical separation from the shortlisted competitors, restricted to one-step absolute-error loss. Thus, pooled national metrics identify the best burden-weighted model, regime-wise metrics summarize error within epidemiological states, and district-wise metrics show local competitiveness.

The results separate three tasks: outbreak magnitude forecasting, outbreak warning, and probabilistic calibration. Prophet was best for aggregate count-scale forecasting. SARIMAX was best for one-step outbreak alerting, with the highest precision, recall, F1, and ROC-AUC. Prophet, MLR, and attention-LSTM showed high precision but low recall. TFT provided quantile forecasts, but its uncertainty performance was weak: 80% intervals were under-calibrated, and outbreak-period pinball losses were much higher than normal-period losses. Thus, warning capability, magnitude accuracy, and calibration did not align across models. We used shared predictors but different model-specific mechanisms based on their architectures. Across MLR coefficients, XGBoost SHAP, and attention-LSTM permutation importance, the recurring predictors were recent serotype information, seasonal timing and delayed climatic variables [36]. However, XGBoost relied mostly on DENV-1 (Lag 1) at both short and long horizons. It shows that recent serotype history was the dominant non-linear signal [38,40]. MLR emphasized seasonal phase and calendar structure at short horizons. Attention-LSTM depended on short-term autoregressive memory, with predictor effects weakening after horizon 2. Sensitivity results exhibits epidemiological relevance may not always obtain predictive gain. Serotype removal improved some models and the simpler non-seasonal SARIMAX outperformed the seasonal baseline which suggests that feature groups and assumptions should be evaluated by forecast objective.

Granger analysis pointed to delayed temperature as pooled climatic precursor. PCMCI gave a more district-specific picture, with links among serotype and climate variables. Serotype variables appeared important but not invariably predictive across models. This suggests a layered pattern: climate provides background context, serotype history may add short-term epidemiological information, seasonality structures timing, and population density may represent accumulated district risk. Their importance varies by model and horizon. Model choice should follow the decision objective which is supported by several studies [25,50]. Prophet is suited to magnitude forecasting, SARIMAX to short-range alerting, and MLR to baseline analysis. Evaluation should therefore include regime-wise error, alert performance, and calibration, not only pooled RMSE and MAE. Related dengue decision-support studies address control and cost-effectiveness [67,68]; this study focuses on horizon-dependent forecasting and early-warning performance.

There are several limitations of this study. The pooled district-panel structure improved comparability but may have limited district-specific flexibility. The test period was dominated by the 2022–2023 epidemic. The model-specific outbreak thresholds helped within-model interpretation but reduced direct comparability of alert metrics. Although the proposed framework is adaptable, the present validation is limited to one country-specific district panel and one epidemic context; therefore, external validation in other geographical and epidemiological settings is needed.

## Conclusion

This study proposes an adaptable, horizon-dependent framework for dengue forecasting and applies it to Bangladesh as a case study. No single model performed best across all tasks. SARIMAX was most useful for short-term forecasting and one-step outbreak alerting. However, Prophet performed best for pooled magnitude forecasting at horizons 2–6. MLR was conservative in regime-wise evaluation. Aggregate accuracy did not fully reflect outbreak performance or uncertainty quality. Regime-wise errors, outbreak-classification results, and TFT calibration showed that models could perform differently during outbreak periods than suggested by pooled metrics. Model evaluation should therefore include outbreak-focused and uncertainty-based measures in addition to RMSE and MAE. Interpretability and causal analyses showed that serotype information, seasonal timing, lagged climatic variables, and district risk all contributed to forecasting. Dengue forecasting should be treated as a tactical-versus-strategic decision problem rather than a globally optimal algorithm. Future work should extend uncertainty estimation, add local relevant covariates, and externally validate the adaptability in other geographical and epidemiological settings.

## Supporting information

S1 Data Author generated code and data. BMD meteorological data were obtained through paid access and cannot be redistributed; a synthetic dataset, code, and metadata are provided at https://doi.org/10.5281/zenodo.19589534 to support reproducibility.
(ZIP)

S1 Fig Attention-LSTM interpretability results. Aggregate permutation-importance plot and attention-weight profile by forecast horizon.
(ZIP)

S1 Table Detailed MLR coefficient results across forecast horizons. Includes aggregate standardized coefficients and horizon-specific coefficient tables for horizons 1 and 6.
(ZIP)

S2 Table Full sensitivity-analysis results across forecast horizons. Per-horizon sensitivity summaries for the selected models and additional models not shown in the main text.
(ZIP)

S3 Table Full pairwise Diebold–Mariano testing results. Complete squared-error and absolute-error comparisons across shortlisted models and horizons.
(ZIP)

S4 Table District-wise PCMCI and pooled Granger-causality summary files. Detailed district-level PCMCI summaries and Granger lag summaries used to support the main causal-analysis figures and tables.
(ZIP)

S5 Table Feature descriptions and district-wise statistics. This supplementary file contains the feature descriptions along with district-wise summary statistics.
(ZIP)
