# Supplementary material for: Tactical vs. strategic: An adaptable framework for horizon-dependent dengue forecasting using data-driven approaches with serotype and climate covariates in Bangladesh
Source: PLoS One. 2026 Aug 21;21(8):e0353069. doi: 10.1371/journal.pone.0353069 (PMC13496456; doi:10.1371/journal.pone.0353069)
Supplement: Supplementary file 1 [file pone.0353069.s008.pdf]

Manuscript Evaluation Report

Manuscript Title

Tactical vs. Strategic: A Generalizable Framework for Horizon-Dependent Dengue Forecasting with a Case Study in Bangladesh

Summary of the Manuscript

This study proposes a generalizable multi-horizon dengue forecasting framework that integrates circulating serotype and meteorological data, using Bangladesh as a case study. By combining temporal cross-validation, horizon- and regime-specific evaluation, and interpretability analyses (SHAP and non-linear causal inference), the results show that no single model dominates across all lead times—SARIMAX performs best for short-term forecasts, while the Temporal Fusion Transformer (TFT) is more effective for longer horizons.

Building on these findings, the following comments aim to improve the clarity and consistency of the framework and results presentation. Addressing them will strengthen how the contributions and conclusions are communicated.

Minor Comments

1. Please clarify how the "dominant" label is determined for each year by defining what "type" refers to and stating the exact rule used to select the dominant category (e.g., the category with the highest value or frequency), with a brief example if possible.

2. There are inconsistencies in formatting, quotation usage, and punctuation around terms and names. Please correct this type of typographical inconsistency consistently throughout the manuscript.

3. In Table 2, please add adequate spacing between the Unit and Domain-Based Class columns to improve readability and prevent the entries from appearing merged.

4. Please revise punctuation for figure citations and ensure consistent use of parentheses (e.g., write "...with excess zeros (Fig. 3b)" rather than leaving the figure reference outside the sentence structure).

5. Please include the missing figure numbers and cite them consistently in parentheses throughout the manuscript.

6. Please add proper spacing for consistency, e.g., $AvgTemp_lag_3(r = 0.372)$.
7. Use a semicolon when listing definitions so each item is clearly separated (e.g.,τ: forecast horizon (steps ahead), with $\tau \in \{1, \ldots, 6\}$.).
8. Quotation marks are used inconsistently; please replace them with proper LaTeX quotes (e.g., “Normal” instead of ”Normal”) throughout the manuscript.

To further strengthen the empirical evidence and align the evaluation with public-health decision needs, the analysis section could be expanded in a few targeted ways. These additions would better quantify performance during rare, high-impact outbreak periods and improve the interpretability of horizon-dependent results.

Major Comments

1. In the figures, key text elements (e.g., axis tick labels, legend entries, and titles) are too small and difficult to read. Please increase the font sizes across all plots to ensure clear visibility and consistent readability.
2. Figures 5 and 6 are currently presented in grayscale; using distinct colors would make the plotted elements easier to distinguish and improve overall readability.
3. Add uncertainty and calibration results for the quantile-based TFT using coverage and pinball loss, ideally reported separately for Normal and Outbreak periods.
4. Using a time-series aware significance test, such as a block bootstrap or rolling-window paired tests, would better account for autocorrelation in forecast errors.
5. Presenting feature-group ablation experiments (climate-only, serotype-only, climate plus serotype, then adding demography and case-lags) would clarify which inputs drive performance at each horizon.
6. Treating outbreak prediction as a separate early-warning task and summarizing it with precision, recall, F1, or ROC-AUC would strengthen the “actionable alerts” discussion.

   After these revisions are addressed, the manuscript would be suitable for acceptance.

Recommendation

I recommend acceptance of the manuscript after the authors comprehensively address both the minor and major issues outlined above.
