# Supplementary material for: Tactical vs. strategic: An adaptable framework for horizon-dependent dengue forecasting using data-driven approaches with serotype and climate covariates in Bangladesh
Source: PLoS One. 2026 Aug 21;21(8):e0353069. doi: 10.1371/journal.pone.0353069 (PMC13496456; doi:10.1371/journal.pone.0353069)
Supplement: Supplementary file 2 [file pone.0353069.s009.pdf]

# Reviewer Report

Tactical vs. Strategic: A Generalizable Framework for Horizon-Dependent Dengue Forecasting with a Case Study in Bangladesh


## Summary

This manuscript proposes a horizon-dependent dengue forecasting framework for Bangladesh, comparing statistical, machine learning, and deep learning models across 1–6 month forecasting horizons. The main contribution is the claim that no single model performs best across all forecast lengths: SARIMAX appears stronger for short-term forecasts, while TFT performs better for longer horizons. The manuscript also incorporates serotype-related covariates and includes SHAP/PCMCI-based interpretability analyses. The practical conclusion is that, although the models do not estimate outbreak magnitude accurately, they may still provide useful early-warning signals.


## Major Concerns

### 1. Data leakage risk in preprocessing

A major methodological concern is the statement that missing values created by lag construction were filled using "the value of the same month of the next year." This introduces future information into the forecasting pipeline and creates a clear leakage risk. Even if this affects only a limited number of observations, it compromises the causal integrity of the forecasting setup. This preprocessing step should be removed and the experiments rerun with a strictly leakage-free procedure.

### 2. Unclear treatment of panel data versus single-series modeling

The manuscript describes a district-level monthly panel with 924 observations across 11 districts from 2017–2023. However, the TFT section refers to a "single-series dataset with a chronological split." This is inconsistent or at least insufficiently explained. It is not clear whether the models were trained on district-level panel data, on a national aggregate series, or on some pooled multi-series formulation. This should be clarified precisely for each model family, especially TFT, LSTM, and Attn-LSTM.

### 3. Serotype covariates are not sufficiently defined

The manuscript emphasizes serotype variables as a key novelty, but their construction and temporal alignment are not described clearly enough. In one place the data are framed as monthly, but elsewhere the dominant serotype appears to be derived for a given year. If the serotype information is annual or sparsely observed while the forecasting problem is monthly, then the feature construction process must be described carefully. As written, this important contribution is not sufficiently reproducible.

**4. Questionable categorical encoding**

The manuscript states that dominant serotype and its lagged versions were encoded using a label encoder that "preserved their ordinal relationships." However, serotype categories do not have a natural ordinal structure. This encoding can impose an artificial ordering and potentially bias the models. The authors should justify this choice or use an alternative encoding method that does not inject false ordinality.

**5. Incomplete manuscript preparation**

The current version still contains unresolved placeholders such as "Fig ??", "Fig (??)", and "Table ??." Page 15, 16 and in a few more places of your submission. These missing references are not merely cosmetic; they prevent proper evaluation of important methodological components such as feature screening and hyperparameter tuning details. The manuscript should be fully cleaned and completed before resubmission.

**6. Incomplete reproducibility**

The data availability statement indicates that code and processed datasets will be made available upon acceptance, while some meteorological data are restricted. For a benchmarking-focused forecasting paper, reproducibility details should be stronger. The manuscript should specify exactly what processed data, code, hyperparameter configurations, random seeds, and train/validation/test split definitions will be shared.

**7. Practical early-warning claims need stronger support**

The discussion argues that even when the models underestimate outbreak magnitude, they may still be useful as early-warning tools. This is plausible, but the manuscript does not directly evaluate alert performance. If the authors wish to make operational or policy-facing claims, they should supplement regression metrics with threshold-based outbreak detection metrics such as sensitivity, specificity, precision, recall, or event-based warning performance.

**8. Recommended References**

The authors may consider citing the following recent and relevant works:

- https://doi.org/10.1016/j.cnsns.2025.109246
- https://doi.org/10.3329/ganit.v43i1.67858
- https://doi.org/10.1016/j.aej.2025.12.039
- https://doi.org/10.1016/j.actatropica.2025.107587

## Minor Concerns

- The abstract and keywords contain wording issues, including the phrase "Temporal Fusion Transfer," which should be corrected to "Temporal Fusion Transformer."

- The grammar and phrasing need language polishing throughout the manuscript.
- The exact calendar ranges for training, validation, purge gap, and testing should be explicitly listed.
- The rationale for reducing the feature set to a final selected subset is not fully transparent and should be described more rigorously.
- It would help to include uncertainty summaries around per-horizon metrics and possibly a compact summary table showing the best-performing model by horizon.

## Specific Revision Requests

1. Remove leakage-prone imputation and rerun the experiments using a strictly causal preprocessing pipeline.
2. Clarify exactly how the district-level panel was represented for each model, especially the deep learning models.
3. Describe the serotype data source and temporal alignment in detail, including whether the data are monthly, yearly, sparse, or imputed.
4. Reconsider the encoding of categorical serotype variables and justify the final choice.
5. Replace or supplement the current significance analysis with a time-series-aware comparison method.
6. Resolve all placeholder table and figure references.
7. Strengthen reproducibility by specifying exact data splits, hyperparameter search spaces, random seeds, and sharing plans.

## Recommendation

### Major Revision

The central idea is worthwhile and the horizon-dependent framing is potentially valuable for dengue forecasting research. However, the current version has important methodological ambiguities, at least one serious data leakage concern, incomplete reporting, and practical claims that are not yet fully supported by the evaluation. These issues are potentially fixable, but substantial revision is needed before the manuscript can be considered for publication.
