# Supplementary material for: Tactical vs. strategic: An adaptable framework for horizon-dependent dengue forecasting using data-driven approaches with serotype and climate covariates in Bangladesh
Source: PLoS One. 2026 Aug 21;21(8):e0353069. doi: 10.1371/journal.pone.0353069 (PMC13496456; doi:10.1371/journal.pone.0353069)
Supplement: Supplementary file 3 [file pone.0353069.s011.pdf]

Reviewer Report

Tactical vs. strategic: A generalizable framework for horizon-dependent dengue forecasting with a case study in Bangladesh

- The manuscript is now generally well structured, but the claim of a "generalizable framework" in the title and discussion should still be stated a little more cautiously. The framework is promising and well motivated, but the present study demonstrates it on one country case study with one specific panel structure and one epidemic context. It may be better to describe it as a transferable or adaptable framework unless broader validation is provided. This point is most relevant in the *Title*, *Methodological Framework & Case Study Setup*, *Discussion*, and *Conclusion*.
- The treatment of serotype information is interesting and potentially novel, but the manuscript would benefit from slightly clearer explanation of how the annual "dominant serotype" variable was constructed and how this annual information aligns with monthly forecasting targets. Since the final selected feature set includes lagged serotype variables and the discussion emphasizes serotype importance, a short clarification in *Dataset Development for Comprehensive Analysis* and *Feature Engineering* would improve reproducibility.
- The feature-selection procedure is reasonable, but the final manual selection step should be described a little more transparently. At present, the manuscript explains the correlation filter, VIF filtering, and SelectKBest screening, then states that the final predictors were chosen manually based on relevance and domain knowledge. A brief statement clarifying how that final decision was made would improve confidence in the modeling pipeline. This is most relevant in the *Feature Selection*.
- The evaluation design is one of the strong parts of the paper, especially the separation of overall, per-horizon, regime-wise, and outbreak-detection performance. However, because the outbreak thresholds are model-specific in Table 9, the comparison of alert metrics across models becomes a little harder to interpret directly. I suggest adding one sentence in the *Evaluation Protocol* and *Practical Implications: From Magnitude Failure to Actionable Alerts* sections to explain this trade-off more explicitly.
- The statistical-significance analysis is useful and much clearer now. Still, the manuscript should make sure the reader understands that the differences between Prophet and the shortlisted competitors are generally modest, with significance appearing mainly for one-step absolute-error comparisons. This is already partly stated, but a slightly more explicit caution in the *Pairwise significance testing* section and *Discussion* would help avoid overinterpretation.
- The sensitivity-analysis results are interesting, especially the finding that removal of serotype variables improved some models and that a simpler non-seasonal SARIMAX outperformed the main seasonal specification. These findings are important and deserve a bit more interpretation in the *Sensitivity and Uncertainty Analysis* section and *Discussion*, because they suggest that some feature groups or baseline assumptions may not contribute uniformly across architectures.
- The interpretability and causal-analysis sections add value to the manuscript, but the distinction

between predictive usefulness and causal importance should be stated very carefully. The current text is already moving in the right direction, especially when discussing serotype centrality versus forecasting gain, and I encourage the authors to preserve this cautious wording. A small amount of polishing in *Model Interpretability and Feature Importance* and *Causal Inference Analysis* would further strengthen the paper.

- The discussion is thoughtful and one of the stronger parts of the manuscript. The tactical-versus-strategic framing is useful and helps explain why different models perform best for different objectives. I would only suggest some light language polishing in the *Discussion* and *Conclusion*, especially in places where the phrasing is slightly awkward or grammatically rough.

- The authors may consider citing a few recent related works to further strengthen the positioning of the manuscript within the current forecasting and mathematical-modeling literature, for example:
  https://doi.org/10.1016/j.cnsns.2025.109246
  https://doi.org/10.3329/ganit.v43i1.67858
  https://doi.org/10.48550/arXiv.2604.18642
  https://doi.org/10.1016/j.aej.2025.12.039
  where relevant to the revised discussion and methodological context.

- Overall, this is a solid and improved manuscript with a relevant case study, a well-motivated framework, and a practically useful evaluation perspective. The remaining issues are mostly about wording, clarification, and slight moderation of some claims.

Minor Revision
