# Supplementary material for: Tactical vs. strategic: An adaptable framework for horizon-dependent dengue forecasting using data-driven approaches with serotype and climate covariates in Bangladesh
Source: PLoS One. 2026 Aug 21;21(8):e0353069. doi: 10.1371/journal.pone.0353069 (PMC13496456; doi:10.1371/journal.pone.0353069)
Supplement: Supplementary file 4 [file pone.0353069.s012.pdf]

# Manuscript Evaluation Report

## Manuscript Title

Tactical vs. Strategic: A Generalizable Framework for Horizon-Dependent Dengue Forecasting with a Case Study in Bangladesh

## Comments

1. The interval coverage during the outbreak period appears to be consistently zero across all forecast horizons (Table 8). This requires an explanation.

2. It is recommended to cite the following studies :

   (a) `https://doi.org/10.1371/journal.pone.0342764`

   (b) `https://doi.org/10.26855/abr.2022.12.001`

   (c) `https://doi.org/10.1038/s41598-025-28135-x`

   (d) `https://doi.org/10.4236/am.2022.1310053`

## Recommendation

I recommend acceptance of the manuscript after the authors comprehensively address the issues outlined above.
