# Supplementary material for: Tactical vs. strategic: An adaptable framework for horizon-dependent dengue forecasting using data-driven approaches with serotype and climate covariates in Bangladesh
Source: PLoS One. 2026 Aug 21;21(8):e0353069. doi: 10.1371/journal.pone.0353069 (PMC13496456; doi:10.1371/journal.pone.0353069)
Supplement: Supplementary file 5 [file pone.0353069.s013.pdf]

Response Letter

Manuscript ID PONE-D-26-03317

Tactical vs. strategic: A generalizable framework for horizon-dependent dengue forecasting with a case study in Bangladesh

PLOS ONE

Response to Academic Editor

1. Please ensure that your manuscript meets PLOS ONE's style requirements, including those for file naming.

Thank you for this reminder. We have revised the manuscript and associated submission files to comply with ***PLOS ONE style requirements****, including formatting, structure, figure/table presentation, and file naming conventions. We have carefully checked the revised submission to ensure consistency with the journal guidelines.*

2. Please ensure that your code is shared in accordance with PLOS ONE guidelines and is available without restrictions upon publication.

Thank you for this important requirement. We have ensured that our code sharing fully complies with PLOS ONE's guidelines for transparency and reproducibility.

All author-generated code has been made ***publicly available without restriction*** *via an archived repository with a DOI:*

- *Code repository: https://doi.org/10.5281/zenodo.19589534*

The repository includes:

- *Full training scripts for all models (e.g., SARIMAX, TFT, and deep learning models)*
- *Complete preprocessing pipeline*
- *Evaluation and analysis scripts*
- *Hyperparameter configurations*
- *Environment specifications, including package versions and Python setup*

To address data sharing constraints, meteorological data obtained from the Bangladesh Meteorological Department cannot be publicly distributed due to access restrictions. To ensure reproducibility despite this limitation, we have provided a synthetic dataset that preserves the structure and statistical characteristics of the original data, allowing all code components to be executed and validated. The shared materials therefore enable full reproducibility of the workflow, including preprocessing, model training, and evaluation, in accordance with best practices.

3. Please update your submission to use the PLOS LaTeX template.

Thank you for this instruction. We have updated the manuscript using the ***PLOS LaTeX template*** *and revised the submission files accordingly to align with the journal's LaTeX formatting requirements.*

4. The repository noted in the Data Availability statement does not qualify as an acceptable repository. Please upload the minimal dataset necessary to replicate the study to a stable public repository.

Thank you for highlighting this requirement. We have revised our data sharing approach to comply with PLOS ONE's data availability standards. We have deposited the materials in a ***public, stable repository with a DOI****:*

- *Repository: https://doi.org/10.5281/zenodo.19589534*

Due to access restrictions, the meteorological data obtained from the Bangladesh Meteorological Department cannot be publicly shared. However, to ensure reproducibility, we have provided a ***synthetic dataset*** *that preserves the structure and statistical properties of the original data, along with all necessary preprocessing and modeling scripts.*

This dataset, together with the shared code and documentation, enables replication of the full analytical workflow, including preprocessing, model training, and evaluation.

5. Please revise the data availability statement and clarify your data sharing plan, noting that all data must be freely accessible upon publication unless an exemption applies.

Thank you for this clarification. We have revised our Data Availability Statement to provide a clear and complete data sharing plan in line with PLOS ONE requirements.

All code, documentation, and a reproducible dataset have already been made publicly available in a stable repository with a DOI:

- *Repository: https://doi.org/10.5281/zenodo.19589534*

Due to third-party restrictions, the meteorological data obtained from the Bangladesh Meteorological Department cannot be made publicly available, as redistribution is not permitted under the data provider's terms.

To ensure reproducibility, we have provided a synthetic dataset that preserves the structure and statistical properties of the original data, along with full preprocessing, modeling, and evaluation code. This allows the complete workflow to be replicated without access to the restricted data. We respectfully request that this be considered as a valid case for restricted data exemption, and we have updated the Data Availability Statement accordingly to explain these constraints.

6. Please amend either the abstract on the online submission form or the abstract in the manuscript so that they are identical.

Thank you for noting this discrepancy. We have revised the abstract so that the version in the manuscript and the version entered in the online submission system are now identical.

7. Figure 1 may contain copyrighted map images. Please provide permission or replace the figure with CC BY–compatible content.

Thank you for raising this important point. We confirm that Figure 1 does not use proprietary sources such as Google Maps or other restricted platforms.

The map is based on administrative boundary data obtained from the Humanitarian Data Exchange (Bangladesh Subnational Administrative Boundaries dataset), which is derived from open and publicly available sources. We have verified that the dataset is compatible with open-use requirements.
To ensure full compliance with PLOS ONE's CC BY 4.0 licensing policy, we have:

- *Clearly cited the data source in the figure caption*
- *Updated the caption to reflect that the map is created from open administrative boundary data*
- *Confirmed that no copyrighted or proprietary basemaps are used*

The revised figure and caption now comply with the journal's licensing requirements.

8. Please ensure that you refer to Figures 2, 4 and 11 in your text as, if accepted, production will need this reference to link the reader to the figure..

Thank you for this observation. Following revisions to the analysis and figures, we have updated the manuscript to ensure that all figures are correctly numbered and consistently referenced in the text.
Specifically:

- *Figures previously labeled as 2 and 4 have been renumbered and updated, and are now properly cited in the revised manuscript*
- *Figure 11 has been removed as part of the revised analysis*

All figure references have been carefully checked to ensure consistency and correct linkage throughout the manuscript.

9. We note you have included tables that were not properly referred to in the text. Please ensure that Tables 1-4 are referred to in the manuscript.

Thank you for this observation. We have carefully revised the manuscript to ensure that all tables retained in the main text are explicitly referred to and discussed in the appropriate places.

In addition, to improve clarity and reduce length, we reorganized the tabular material during revision. Non-essential descriptive tables were moved to the Supporting Information or summarized in the text, while only the most relevant tables were retained in the main manuscript. All remaining tables are now consistently numbered and properly cited in the revised manuscript.

10. If the reviewer comments include a recommendation to cite specific previously published works, please evaluate whether they are relevant and should be cited.

Thank you for this clarification. We carefully reviewed the publications recommended by the reviewers and evaluated their relevance to the scope, methodology, and objectives of our study. Where appropriate, we have incorporated relevant citations into the revised manuscript. For works that were not directly aligned with the present study, we did not add citations.

Response to Reviewer #1

Thank you for handling and reading our manuscript. We have addressed all of your comments as follows:

##Minor Comments

1. Please clarify how the "dominant" label is determined for each year by defining what "type" refers to and stating the exact rule used to select the dominant category (e.g., the category with the highest value or frequency), with a brief example if possible.

Thank you for this suggestion. We have clarified the definition of the dominant serotype variable in the revised manuscript. Here, "type" refers to the dengue serotype category (DENV-1 to DENV-4). For each year, the dominant label was defined as the serotype with the highest annual value/frequency in the compiled serotype data, and that label was assigned to the monthly observations for that year. For example, if DENV-2 was the most frequent serotype in a given year, the dominant label for that year was recorded as DENV-2. We have added this explanation to the manuscript to make the construction of the variable explicit and reproducible.

2. There are inconsistencies in formatting, quotation usage, and punctuation around terms and names. Please correct this type of typographical inconsistency consistently throughout the manuscript.

Thank you for this careful observation. We have performed a thorough proofreading of the revised manuscript and corrected typographical inconsistencies throughout. In particular, we standardized formatting, punctuation, capitalization, quotation usage, and the presentation of terms, names, and figure/table references to improve consistency and readability across the manuscript.

3. In Table 2, please add adequate spacing between the Unit and Domain-Based Class columns to improve readability and prevent the entries from appearing merged.

Thank you for this suggestion. To improve readability and overall manuscript clarity, Table 2 has been moved to the Supporting Information as part of the revised manuscript structure. In the supplementary version, we have also improved the formatting and spacing, including clearer separation between the Unit and Domain-Based Class columns.

4. Please revise punctuation for figure citations and ensure consistent use of parentheses (e.g., write "…with excess zeros (Fig. 3b)" rather than leaving the figure reference outside the sentence structure).

Thank you for pointing this out. We have revised the manuscript to ensure that figure citations are punctuated consistently and integrated properly within sentence structure, including the consistent use of parentheses in the PLOS style (e.g., "(Fig. X)"). All figure references were reviewed and corrected throughout the manuscript for consistency.

5. Please include the missing figure numbers and cite them consistently in parentheses throughout the manuscript.

Thank you for this observation. We have carefully revised the manuscript to resolve the missing figure numbers and to ensure that all figures are now cited consistently in parentheses throughout the text. We also checked figure numbering globally after revision so that all in-text references match the final figure order.

6. Please add proper spacing for consistency, e.g., AvgTemp_lag_3 (r = 0.372).

Thank you for this suggestion. We have revised the manuscript to improve formatting and readability by standardizing the presentation of variable names and statistical notation. For example, terms such as AvgTemp_lag_3 (r = 0.372) have been reformatted to a clearer style (e.g., Avg. Temp (lag 3)), and spacing has been made consistent throughout the manuscript.

7. Use a semicolon when listing definitions so each item is clearly separated (e.g.,τ: forecast horizon (steps ahead), with $\tau\in\{1,\ldots,6\}$.).

Thank you for this suggestion. We revised the presentation of notation and symbol definitions to improve clarity and consistency. In the revised manuscript, notation is now presented more clearly in the nomenclature table and related mathematical descriptions, so that individual definitions are separated and easier to read. We also standardized punctuation and formatting in notation-related text throughout the manuscript.

8. Quotation marks are used inconsistently; please replace them with proper LaTeX quotes (e.g., ``Normal" instead of "Normal") throughout the manuscript.

Thank you for this observation. We have revised the manuscript to standardize quotation mark usage throughout and replaced inconsistent quotation formatting with proper LaTeX quotation style where applicable. This was checked across the full manuscript to improve consistency and presentation.

##Major Comments:

1. In the figures, key text elements (e.g., axis tick labels, legend entries, and titles) are too small and difficult to read. Please increase the font sizes across all plots to ensure clear visibility and consistent readability.

Thank you for this important observation. We have revised all figures in the manuscript to improve readability by increasing font sizes for axis labels, tick labels, legends, and titles. We also ensured consistent formatting across all plots so that text elements are clearly visible both on screen and in print.

2. Figures 5 and 6 are currently presented in grayscale; using distinct colors would make the plotted elements easier to distinguish and improve overall readability.

Thank you for this suggestion. We revised the figure set to improve visual clarity and distinguishability. In the revised manuscript, the relevant figures were updated to use distinct colors instead of grayscale where appropriate. Because the figure set was reorganized during revision, the original numbering also changed: the figure previously cited as Figure 5 was renumbered, and the original Figure 6 was removed as part of the revised analysis. More broadly, we applied improved color formatting across the figures to enhance readability throughout the manuscript.

3. Add uncertainty and calibration results for the quantile-based TFT using coverage and pinball loss, ideally reported separately for Normal and Outbreak periods.

Thank you for this valuable suggestion. We have expanded the revised manuscript to include an explicit uncertainty and calibration analysis for the quantile-based TFT model. Specifically, we now report interval coverage, mean interval width, and quantile pinball loss, and we present these results separately for Normal and Outbreak periods across forecast horizons. This addition is included in the revised Results section(Uncertainty and calibration analysis) and provides a clearer assessment of TFT's probabilistic performance and calibration under both routine and epidemic conditions. (Table 8: TFT regime-wise 80% interval coverage, mean interval width, and pinball loss by forecast horizon.)

**Hor.**	**Normal**					**Outbreak**				
	**Cov80**	**Width80**	**$PB_{0.10}$**	**$PB_{0.50}$**	**$PB_{0.90}$**	**Cov80**	**Width80**	**$PB_{0.10}$**	**$PB_{0.50}$**	**$PB_{0.90}$**
1	0.238	1.268	2.673	13.275	23.147	0.000	1.779	243.837	1218.994	2192.929
2	0.226	1.542	2.666	13.177	22.866	0.000	1.957	243.825	1218.861	2192.661
3	0.179	1.989	2.661	13.045	22.438	0.000	2.143	243.816	1218.772	2192.418
4	0.188	2.188	2.791	13.645	23.430	0.000	2.238	246.340	1231.370	2215.043
5	0.174	2.290	2.924	14.286	24.520	0.000	2.214	248.919	1244.273	2238.279
6	0.172	2.303	3.056	14.948	25.695	0.000	2.203	251.554	1257.447	2262.005

Cov80 denotes the proportion of observations falling within the predicted 80% interval. Width80 denotes the mean width of the 80% prediction interval on the count scale. PB_q denotes the pinball loss at quantile q.

4. Using a time-series aware significance test, such as a block bootstrap or rolling-window paired tests, would better account for autocorrelation in forecast errors.

Thank you for this thoughtful suggestion. We agree that statistical comparison of forecasting models should account for temporal dependence in forecast errors. In the revised manuscript, we addressed this concern by adopting a time-series-aware ***Diebold-Mariano*** *testing framework with the Harvey-Leybourne-Newbold small-sample correction and Newey-West long-run variance estimation, with lag selected as a function of forecast horizon, presented in the result section(Pairwise significance testing); Table 10. Pairwise Diebold–Mariano significance testing comparing Prophet with shortlisted competing models across horizons.*

**Panel A. Squared-error loss**					**Panel B. Absolute-error loss**			
**Horizon**	**vs ATT-LSTM**	**vs S-LSTM**	**vs XGB**	**vs TFT**	**vs ATT-LSTM**	**vs S-LSTM**	**vs XGB**	**vs TFT**
H1	0.123	0.123	0.123	0.123	**0.018**	**0.018**	**0.018**	**0.018**
H2	0.694	0.694	0.694	0.694	0.275	0.327	0.275	0.275
H3	1.000	1.000	1.000	1.000	0.619	0.619	0.619	0.619
H4	1.000	1.000	1.000	1.000	0.879	0.957	0.879	0.879
H5	1.000	1.000	1.000	1.000	0.999	0.999	0.999	0.999
H6	1.000	1.000	1.000	1.000	1.000	1.000	1.000	1.000

Entries are Holm-adjusted p-values; bold indicates $p < 0.05$. Tests were performed on aligned common test rows using count-scale forecasts. Losses were averaged across districts within each TargetDate. Reported values are Holm-adjusted p-values from the Harvey–Leybourne–Newbold corrected Diebold–Mariano test.

We therefore revised the significance-analysis section to use a temporally appropriate comparison procedure, even though we did not implement the specific alternatives suggested by the reviewer (e.g., block bootstrap or rolling-window paired tests). This updated procedure is now described in the manuscript and the corresponding results are reported in the revised Results section.

5. Presenting feature-group ablation experiments (climate-only, serotype-only, climate plus serotype, then adding demography and case-lags) would clarify which inputs drive performance at each horizon.

Thank you for this constructive suggestion. We agree that feature-group comparisons can help clarify which inputs drive forecasting performance across horizons. In the revised manuscript, we addressed this issue through a feature-group sensitivity analysis based on one-factor-at-a-time ablation, using the same preprocessed panel, split structure, and horizon definitions as in the main experiments. Specifically, we compared the retained full specification against variants with climate variables, serotype variables, temporal variables, or population density removed, depending on model class. This allowed us to assess how the exclusion of each feature group affected performance overall and across forecast horizons. The sensitivity analysis results are presented in the revised manuscript (Table 7. Best-performing overall sensitivity specification by model.)

Model	Best RMSE spec.	RMSE	Best MAE spec.	MAE
Prophet	Full	3974.869	Full	1241.106
MLR	No serotype	4082.544	No serotype	1266.926
Attention-LSTM	No serotype	3932.587	No serotype	1189.481
SARIMAX	Simpler non-seasonal	4178.289	Simpler non-seasonal	1424.630

No month dummies removes the month-indicator regressors. No serotype removes the serotype predictors. Simpler non-seasonal removes the seasonal ARIMA terms.

We note that this is not identical to the exact staged buildup design suggested by the reviewer (e.g., climate-only, serotype-only, climate plus serotype, then adding demography and case-lags). However, we revised the manuscript to provide a structured feature-group ablation analysis that addresses the same underlying objective of identifying the contribution of major predictor groups to forecasting performance. The updated sensitivity-analysis design and results are now reported in the manuscript.

6. Treating outbreak prediction as a separate early-warning task and summarizing it with precision, recall, F1, or ROC-AUC would strengthen the ``actionable alerts" discussion.

Thank you for this important suggestion. We agree that outbreak prediction should be evaluated as a distinct early-warning task, rather than being inferred only from regression-based forecast accuracy. In the revised manuscript, we therefore added a dedicated outbreak-classification evaluation based on the test set. Specifically, we now report precision, recall, F1-score, specificity, ROC-AUC, and PR-AUC for outbreak detection, with results presented separately from the count-scale forecasting metrics, presented in the revised manuscript (Table9.One-step-ahead outbreak-classification performance on the test set.)

Model	Threshold	Precision	Recall	F1	ROC-AUC
SARIMAX	196.6	0.886	0.824	0.854	0.950
Prophet	124.2	0.923	0.130	0.229	0.871
MLR	80.0	1.000	0.100	0.182	0.905
SVR	80.0	0.000	0.000	0.000	0.232
RF	80.0	0.000	0.000	0.000	0.558
XGBoost	80.0	0.000	0.000	0.000	0.695
CatBoost	80.0	0.000	0.000	0.000	0.727
Stacked LSTM	94.0	1.000	0.041	0.079	0.836
Attention-LSTM	94.0	1.000	0.124	0.220	0.842
TFT	124.2	0.000	0.000	0.000	0.598

Metrics are reported for one-step-ahead test forecasts ($h = 1$). The outbreak threshold is model-specific and was defined as the 90th percentile of the training-set dengue counts on the count scale.

We also revised the discussion to distinguish more clearly between outbreak-magnitude forecasting and actionable alert performance, which led to a more precise interpretation of the practical role of each model. The corresponding methodology and results are now included in the revised evaluation and results sections.

Response to Reviewer #2

Thank you for handling and reading our manuscript. We have addressed all of your comments as follows:

## Overall Comments

The manuscript addresses an important public health problem and presents a useful horizon-dependent comparison of dengue forecasting models, which is a clear strength. However, the current version needs substantial revision before it can be considered further. In particular, the preprocessing pipeline raises a serious concern about possible data leakage, the handling of district-level panel data versus single-series modeling is not sufficiently clear, and the construction and encoding of serotype-related covariates need better justification and explanation. In addition, the statistical comparison framework should better account for temporal dependence, several figure/table placeholders remain unresolved, and the reproducibility details are still incomplete. The practical early-warning discussion is promising, but it would be stronger if supported by explicit outbreak-detection metrics. Overall, the study has potential, but the methodology and reporting need to be clarified and strengthened.

We thank the reviewer for the careful and constructive assessment of our manuscript. We appreciate the recognition that the horizon-dependent comparison addresses an important public-health problem. In response to the concerns raised, we substantially revised the manuscript to strengthen the methodology, clarify the data structure, improve reproducibility, and better support the practical early-warning discussion.

First, we revised the preprocessing pipeline to ensure a strictly causal forecasting setup. The original workflow was replaced with a leakage-free procedure in which lag features are created district-wise after strict chronological ordering, lag-induced missing values are not imputed, and the initial lag burn-in rows are removed before modeling. We also clarified the chronological train/validation/purge/test split and explained the role of purge rows as context rows rather than

evaluation targets. All experiments were rerun under this revised pipeline, and the results, figures, and discussion were updated accordingly.

Second, we clarified the treatment of district-level panel data across model classes. The revised manuscript now states explicitly that the study uses a monthly district-panel dataset covering 11 districts, and it distinguishes the modeling designs more clearly: Prophet and SARIMAX are fitted district-wise and later aggregated to national totals, whereas the machine-learning, LSTM-based, and TFT models use a common district-panel framework with aligned chronological splits and horizon-specific evaluation. This revision removes the earlier ambiguity about whether some models were trained as single-series formulations.

Third, we improved the description and justification of the serotype-related covariates. The revised manuscript now explains more clearly how the serotype variables are incorporated, how the derived dominant serotype feature is constructed, and how categorical serotype-related inputs are handled during preprocessing. We also clarified that categorical variables were encoded after splitting, using the training-set feature space as the reference for alignment across validation, purge, and test data.

Fourth, we revised the statistical comparison framework to account more appropriately for temporal dependence. In the revised manuscript, pairwise model comparisons are based on the Diebold-Mariano test with Harvey-Leybourne-Newbold correction, using horizon-aware long-run variance estimation and date-level aggregation of loss differences to avoid pseudo-replication across districts. This replaced the earlier, less suitable comparison approach and provides a more appropriate significance-analysis protocol for the forecasting setting considered here.

Fifth, we resolved the reporting issues noted by the reviewer. All remaining figure and table placeholders were removed, figure numbering was checked and updated throughout, and the visual presentation of figures was improved through larger text and clearer formatting. We also revised figure and table citations throughout the manuscript to ensure consistency with the updated structure.

Sixth, we strengthened reproducibility substantially. The revised manuscript now specifies the split structure, preprocessing logic, model evaluation workflow, and hyperparameter/configuration details more clearly. In addition, the full code base, environment information, and reproducibility materials have been archived in a public Zenodo repository, together with a synthetic dataset that allows execution of the workflow when restricted meteorological data cannot be redistributed.

Finally, we strengthened the practical early-warning discussion by treating outbreak prediction as a separate evaluation task. The revised manuscript now reports explicit outbreak-detection metrics, including precision, recall, F1-score, specificity, ROC-AUC, and PR-AUC, and discusses these results separately from pooled count-scale forecasting accuracy. We also added TFT uncertainty and calibration analysis using interval coverage, mean interval width, and pinball loss, reported separately for Normal and Outbreak periods.

Detailed PDF comments point-by-point

<u>##Major Concerns:</u>

1. Data leakage risk in preprocessing: A major methodological concern is the statement that missing values created by lag construction were filled using "the value of the same month of the next year." This introduces future information into the forecasting pipeline and creates a clear leakage risk. Even if this affects only a limited num ber of observations, it compromises the causal integrity of the forecasting setup. This preprocessing step should be removed and the experiments rerun with a strictly leakage-free procedure.

We thank the reviewer for identifying this critical issue. We agree that the earlier imputation strategy was not appropriate for a forecasting setting because it could introduce future information into the preprocessing pipeline.

In response, we revised the preprocessing pipeline to be strictly leakage-free and reran the full analysis under the corrected workflow. In the revised pipeline, the data are first sorted chronologically within each district, lag features are then created district-wise using past values only, and the missing values generated by lag construction are not imputed. Instead, the initial lag burn-in rows are removed before modeling. After this lag-burn-in removal, the dataset is assigned to the chronological train, validation, purge, and test splits, ensuring that all preprocessing and modeling steps preserve temporal causality. The revised manuscript now states this explicitly in the Feature Engineering section: "Lag-induced missing values were not imputed; instead, the initial lag burn-in rows were removed." It also clarifies that the modeling pipeline uses a chronological train-validation-purge-test design.

These changes are also reflected in the revised preprocessing code. The updated workflow creates lags using district-wise shift(lag), keeps the resulting lag-created missing values, removes rows with unavailable lag history via dropna(subset=required_for_model), and assigns splits only after that leakage-free filtering step.

2. Unclear treatment of panel data versus single-series modeling: The manuscript describes a district-level monthly panel with 924 observations across 11 districts from 2017–2023. However, the TFT section refers to a "single-series dataset with a chronological split." This is inconsistent or at least insufficiently explained. It is not clear whether the models were trained on district-level panel data, on a national aggregate series, or on some pooled multi series formulation. This should be clarified precisely for each model family, especially TFT, LSTM, and Attn-LSTM

We thank the reviewer for identifying this inconsistency. We agree that the earlier wording was misleading. After revising the manuscript and rechecking the implementation scripts, we clarified that the study is based on a district-level monthly panel, but the model families use different panel representations, not a single common "single-series" formulation.

More specifically, the revised manuscript now distinguishes the implementations as follows.

For Prophet and SARIMAX, the models are fit district-wise, with one model per district, and national forecasts are obtained only after fitting by summing district-level predictions by target month. This grouped district-level design is explicit in the model scripts and now stated clearly in the manuscript.

For the direct machine-learning models such as MLR, SVR, Random Forest, and CatBoost, the data are represented as a pooled district-month panel in which one row corresponds to one District–Date observation. Horizon-specific targets are then created by assigning each source row at time $t$to its target at $t + h$. In this sense, these models are not trained on a national aggregate series and not on separate single-series datasets; rather, they are trained on a pooled supervised panel, sometimes with district included explicitly as a feature or categorical input. This is stated directly in the scripts, for example in CatBoost ("one row = one District-Date" and "fit on the pooled district-month panel") and similarly in the other direct ML implementations.

For the Stacked LSTM and Attention-LSTM models, the representation is again district-panel, but in sequence form rather than row-wise tabular form. These models construct district-specific source sequences ending at a source date and then pool those sequences into a common training framework. They are therefore not national single-series models. Both scripts explicitly describe them as pooled district-level panel models in a direct multi-horizon setup, with a sequence branch built from district histories and a separate static branch for target-month cyclical features.

For TFT, we corrected the wording most directly. The TFT implementation is not a single national time series. It is a pooled district-level panel / multi-series formulation in which one TFT model is trained jointly across districts, with District used as the grouping variable and with a shared monthly time index across the panel. The script explicitly describes TFT as a district-panel pipeline, with one TFT model predicting all horizons jointly up to the maximum prediction length, rather than a single-series dataset.

To align the manuscript with the actual implementation, we revised the common-framework and model-description sections to state this explicitly:

(i) Prophet and SARIMAX are grouped district-wise benchmarks;
(ii) MLR, SVR, RF, XGBoost, CatBoost, Stacked LSTM, and Attention-LSTM use direct multi-horizon pooled district-panel formulations; and
(iii) TFT uses a joint multi-horizon pooled multi-series district-panel formulation. We also removed the earlier "single-series dataset" wording so that the manuscript now matches the code and evaluation design.

3. Serotype covariates are not sufficiently defined: The manuscript emphasizes serotype variables as a key novelty, but their construction and temporal alignment are not described clearly enough. In one place the data are framed as monthly, but elsewhere the dominant serotype appears to be derived for a given year. If the serotype information is annual or sparsely observed while the forecasting problem is monthly, then the feature construction process must be described carefully. As written, this important contribution is not sufficiently reproducible.

We thank the reviewer for highlighting this important issue. We agree that the earlier version did not describe the construction and temporal alignment of serotype covariates with sufficient clarity.

In the revised manuscript, we have substantially expanded this section to make the feature engineering process fully transparent and reproducible. Specifically, we clarified that the original serotype data are not available at a complete monthly resolution. Therefore, we constructed the serotype-related features using a temporal alignment strategy in which each observation period is assigned a representative serotype composition based on the available surveillance data. In particular, the "dominant serotype" is defined as the serotype with the highest observed proportion within the corresponding aggregation window, and this label is then mapped to the monthly panel in a consistent and forward-looking manner, ensuring no future information is introduced.

To further strengthen the justification for including serotype covariates, we added a causal discovery analysis using PCMCI (GPDC test). The updated results (Fig. 9) show statistically significant lagged dependencies between serotype variables and dengue incidence, alongside climatic covariates, across multiple districts. These findings provide empirical support for the inclusion of serotype features as predictive inputs rather than purely descriptive variables.
In addition, we revised the manuscript to:

- *clearly distinguish between the raw serotype data resolution and the monthly modeling panel,*
- *explicitly describe how serotype features (including lagged variants) are constructed and aligned with the forecasting horizon, and*
- *ensure that all transformations are causally valid and reproducible within the forecasting pipeline.*

4. Questionable categorical encoding: The manuscript states that dominant serotype and its lagged versions were encoded using a label encoder that "preserved their ordinal relationships." However, serotype categories do not have a natural ordinal structure. This encoding can impose an artificial ordering and potentially bias the models. The authors should justify this choice or use an alternative encoding method that does not inject false ordinality.

We thank the reviewer for this important observation. We agree that serotype categories do not have a natural ordinal structure, and that the earlier description implying an ordinal-preserving label encoding was inappropriate.

In the revised analysis, we corrected both the encoding approach and its description in the manuscript. Specifically, categorical serotype-related variables, including dominant serotype and its lagged versions, are now handled using one-hot encoding rather than ordinal label encoding. This avoids imposing any artificial ranking among serotype categories and is more appropriate for nominal epidemiological classes.

The revised preprocessing pipeline now performs categorical encoding after the chronological split is defined, using the training-set feature space as the reference and then reindexing the remaining splits to the same encoded columns. This ensures both causal validity and consistent feature alignment across training, validation, purge, and test sets. The updated preprocessing

script reflects this explicitly: categorical variables such as dominant, dominant_lag_1, dominant_lag_2, and dominant_lag_3 are included in the categorical column list and encoded using pd.get_dummies(...), with the non-training splits reindexed to the training columns.
We also revised the manuscript text to remove the earlier misleading wording and to state clearly that categorical serotype-related inputs were treated as nominal variables and encoded accordingly. This correction eliminates the concern about false ordinality.

5. The current version still contains unresolved placeholders such as “Fig ??”, “Fig (??)”, and “Table ??” (e.g., pages 15–16). These missing references are not merely cosmetic; they prevent proper evaluation of important methodological components such as feature screening and hyperparameter tuning details. The manuscript should be fully cleaned and completed before resubmission.

We thank the reviewer for pointing this out. We agree that the presence of unresolved placeholders affects clarity and completeness of the manuscript.

In the revised version, we have fully resolved all placeholder references, including “Fig ??”, “Fig (??)”, and “Table ??”, and ensured that all figures and tables are properly numbered and consistently cited throughout the text. We also carefully reviewed the manuscript to confirm that all references correspond correctly to the updated figures and tables following the revision of the analysis and pipeline.

6. The data availability statement indicates that code and processed datasets will be made available upon acceptance, while some meteorological data are restricted. For a benchmarking-focused forecasting paper, reproducibility details should be stronger. The manuscript should specify exactly what processed data, code, hyperparameter configurations, random seeds, and train/validation/test split definitions will be shared.

We thank the reviewer for this important suggestion. We agree that reproducibility is critical, particularly for a benchmarking-focused forecasting study.

In the revised manuscript, we have strengthened the reproducibility and transparency of the study. Specifically, we now provide a publicly accessible Zenodo repository (with DOI) that includes:

- *the full codebase, covering preprocessing, feature engineering, model training, evaluation, and visualization pipelines;*
- *model-specific training scripts for all model families (e.g., SARIMAX, Prophet, machine-learning models, LSTM-based models, and TFT);*
- *hyperparameter configurations and tuning setups used in the experiments;*
- *explicit random seed settings to ensure reproducibility of model training and evaluation;*
- *detailed train/validation/purge/test split definitions, including the chronological splitting logic and the role of purge rows for multi-horizon forecasting;*
- *a synthetic/sample dataset that follows the exact structure of the original data, enabling execution of the full pipeline despite restrictions on meteorological data.*

We also revised the manuscript to explicitly describe these components, including the preprocessing workflow, split strategy, and modeling configurations, so that the experimental setup can be reproduced without ambiguity.

7. Practical early-warning claims need stronger support The discussion argues that even when the models underestimate outbreak magnitude, they may still be useful as early-warning tools. This is plausible, but the manuscript does not directly evaluate alert performance. If the authors wish to make operational or policy-facing claims, they should supplement regression metrics with threshold-based outbreak detection metrics such as sensitivity, specificity, precision, recall, or event-based warning performance.

We thank the reviewer for this important suggestion. We agree that evaluating early-warning capability requires metrics beyond regression-based forecast accuracy.

In the revised manuscript, we therefore extended the evaluation framework to treat outbreak detection as a separate task. Specifically, we defined outbreak events based on a threshold derived from the training data and evaluated model outputs using classification-oriented metrics, including precision, recall, F1-score, specificity, ROC-AUC, and PR-AUC, presented in Practical Implications: From Magnitude Failure to Actionable Alerts of Result & Analysis Section (Table 9: One-step-ahead outbreak-classification performance on the test set.)

**Model**	**Threshold**	**Precision**	**Recall**	**F1**	**ROC-AUC**
SARIMAX	196.6	0.886	0.824	0.854	0.950
Prophet	124.2	0.923	0.130	0.229	0.871
MLR	80.0	1.000	0.100	0.182	0.905
SVR	80.0	0.000	0.000	0.000	0.232
RF	80.0	0.000	0.000	0.000	0.558
XGBoost	80.0	0.000	0.000	0.000	0.695
CatBoost	80.0	0.000	0.000	0.000	0.727
Stacked LSTM	94.0	1.000	0.041	0.079	0.836
Attention-LSTM	94.0	1.000	0.124	0.220	0.842
TFT	124.2	0.000	0.000	0.000	0.598

Metrics are reported for one-step-ahead test forecasts ($h = 1$). The outbreak threshold is model-specific and was defined as the 90th percentile of the training-set dengue counts on the count scale.

In addition, we revised the discussion to more clearly distinguish between forecasting outbreak magnitude and detecting outbreak onset, and we moderated the earlier claims to ensure that practical early-warning implications are supported directly by the new evaluation results.

8. The authors may consider citing the following recent and relevant works:
   - https://doi.org/10.1016/j.cnsns.2025.109246
   - https://doi.org/10.3329/ganit.v43i1.67858
   - https://doi.org/10.1016/j.aej.2025.12.039
   - https://doi.org/10.1016/j.actatropica.2025.107587

We thank the reviewer for these helpful suggestions. We have reviewed the recommended references and have incorporated all of them into the revised manuscript, where relevant, to strengthen the background and contextual discussion.

## Minor Concerns

- The abstract and keywords contain wording issues, including the phrase “Temporal Fusion
- Transfer,” which should be corrected to “Temporal Fusion Transformer.”
- The grammar and phrasing need language polishing throughout the manuscript.
- The exact calendar ranges for training, validation, purge gap, and testing should be explicitly listed.
- The rationale for reducing the feature set to a final selected subset is not fully transparent and should be described more rigorously.
- It would help to include uncertainty summaries around per-horizon metrics and possibly a
- compact summary table showing the best-performing model by horizon.

We thank the reviewer for these helpful observations. We have carefully revised the manuscript to address all of these minor concerns. Specifically, we corrected wording issues in the abstract and keywords, including replacing “Temporal Fusion Transfer” with “Temporal Fusion Transformer” where appropriate. We also performed language polishing throughout the manuscript to improve grammar, phrasing, and readability. In addition, we now state the exact calendar ranges for the train, validation, purge, and test periods explicitly in the revised Methods section.

We also strengthened the explanation of the feature-selection procedure by clarifying the sequential screening steps, including correlation filtering, VIF-based reduction, SelectKBest screening, and final manual selection based on target relevance, multicollinearity, and domain considerations. Finally, we expanded the reporting of uncertainty-related results and revised the presentation of performance summaries to improve clarity regarding horizon-dependent model behavior.

## Specific Revision Requests

1. Remove leakage-prone imputation and rerun the experiments using a strictly causal preprocessing pipeline.
2. Clarify exactly how the district-level panel was represented for each model, especially the deep learning models.
3. Describe the serotype data source and temporal alignment in detail, including whether the data are monthly, yearly, sparse, or imputed.
4. Reconsider the encoding of categorical serotype variables and justify the final choice.
5. Replace or supplement the current significance analysis with a time-series-aware comparison method.
6. Resolve all placeholder table and figure references.
7. Strengthen reproducibility by specifying exact data splits, hyperparameter search spaces, random seeds, and sharing plans.

We thank the reviewer for summarizing these revision priorities so clearly. We have addressed all of these requests in the revised manuscript. In particular, we:

(i) removed leakage-prone preprocessing and reran the experiments under a strictly causal pipeline;

(ii) clarified the district-level panel representation for each model family, including the deep-learning models;

(iii) expanded the description of the serotype data source and temporal alignment;

(iv) revised the handling of categorical serotype variables to avoid false ordinality;

(v) replaced the earlier significance framework with a more time-series-aware comparison approach;

(vi) resolved all placeholder figure and table references; and

(vii) strengthened reproducibility by specifying the split structure, shared code/materials, configurations, and sharing plan.
