# Supplementary material for: Tactical vs. strategic: An adaptable framework for horizon-dependent dengue forecasting using data-driven approaches with serotype and climate covariates in Bangladesh
Source: PLoS One. 2026 Aug 21;21(8):e0353069. doi: 10.1371/journal.pone.0353069 (PMC13496456; doi:10.1371/journal.pone.0353069)
Supplement: Supplementary file 6 [file pone.0353069.s014.pdf]

Response Letter

Manuscript ID PONE-D-26-03317

Tactical vs. strategic: An adaptable framework for horizon-dependent dengue forecasting using data-driven approaches with serotype and climate covariates in Bangladesh

PLOS ONE

Response to Academic Editor

Thank you for the opportunity to revise our manuscript. We have carefully addressed the remaining comments and prepared a revised manuscript, a marked-up version, and a clean version for submission. We also reviewed the reference list, added the relevant recommended studies where appropriate, and checked the manuscript for clarity, consistency, and moderated claims. The specific changes are described point-by-point below.

Response to Reviewer #1

Thank you for handling and reading our manuscript. We have addressed all of your comments as follows:

1. Please clarify how the "dominant" label is determined for each year by defining what "type" refers to and stating the exact rule used to select the dominant category (e.g., the category with the highest value or frequency), with a brief example if possible.

Thank you for pointing this out. We have added an explanation in the Sensitivity and Uncertainty Analysis section. We now clarify that the zero outbreak-period coverage in Table 8 means that none of the observed outbreak counts fell within TFT's predicted 80% intervals. The model still produced prediction intervals, but these intervals were systematically below the observed outbreak magnitudes, indicating poor calibration and underestimation of the upper tail during rare epidemic surges.

2. It is recommended to cite the following studies:
    - https://doi.org/10.1371/journal.pone.0342764
    - https://doi.org/10.26855/abr.2022.12.001
    - https://doi.org/10.1038/s41598-025-28135-x
    - https://doi.org/10.4236/am.2022.1310053

Thank you for the recommendation. We have cited the suggested studies in the Introduction to strengthen the manuscript's positioning within the broader infectious-disease modelling literature. These studies are used as methodological background for predictive, stochastic, and mathematical modelling approaches relevant to epidemic assessment, resource allocation, and intervention planning.

Response to Reviewer #2

Thank you for handling and reading our manuscript. We have addressed all of your comments as follows:

1. The manuscript is now generally well structured, but the claim of a "generalizable framework" in the title and discussion should still be stated a little more cautiously. The framework is promising and well-motivated, but the present study demonstrates it on one country case study with one specific panel structure and one epidemic context. It may be better to describe it as a transferable or adaptable framework unless broader validation is provided. This point is most relevant in the Title, Methodological Framework & Case Study Setup, Discussion, and Conclusion.

Thank you for this helpful suggestion. We agree that "generalizable framework" overstated the scope of validation, as the present study is demonstrated using one country-level case study, one district-panel structure, and one epidemic context. We have therefore revised the title and relevant wording throughout the manuscript to describe the approach as an "adaptable framework" rather than a "generalizable framework." We also added cautious wording in the Methodological Framework, Discussion, and Conclusion to clarify that broader transferability would require external validation in other geographical and epidemiological settings.

2. The treatment of serotype information is interesting and potentially novel, but the manuscript would benefit from slightly clearer explanation of how the annual "dominant serotype" variable was constructed and how this annual information aligns with monthly forecasting targets. Since the final selected feature set includes lagged serotype variables and the discussion emphasizes serotype importance, a short clarification in Dataset Development for Comprehensive Analysis and Feature Engineering would improve reproducibility.

Thank you for this helpful comment. We have clarified how the annual serotype variables were aligned with monthly forecasting targets. Specifically, we now state that annual DENV-I--DENV-IV percentage values were assigned to all monthly observations within the corresponding year, and that the dominant serotype variable was defined as the serotype with the highest annual percentage value for that year. We also clarified in the Feature Engineering section that lagged serotype variables were generated after this annual-to-monthly alignment. This revision improves reproducibility and clarifies how annual serotype surveillance information was incorporated into the monthly forecasting framework.

3. The treatment of serotype information is interesting and potentially novel, but the manuscript would benefit from slightly clearer explanation of how the annual "dominant serotype" variable was constructed and how this annual information aligns with monthly forecasting targets. Since the final selected feature set includes lagged serotype variables and the discussion emphasizes serotype importance, a short clarification in Dataset Development for Comprehensive Analysis and Feature Engineering would improve reproducibility.

Thank you for this helpful suggestion. We have revised the Feature Selection section to clarify the final manual selection step. We now explain that SelectKBest was used as a screening step, and

that final predictors were selected from the screened set by prioritizing target relevance, low redundancy after correlation and VIF filtering, temporal availability at the source forecasting month, and representation of key epidemiological, climatic, temporal, and demographic domains

4. The evaluation design is one of the strong parts of the paper, especially the separation of overall, per-horizon, regime-wise, and outbreak-detection performance. However, because the outbreak thresholds are model-specific in Table 9, the comparison of alert metrics across models becomes a little harder to interpret directly. I suggest adding one sentence in the Evaluation Protocol and Practical Implications: From Magnitude Failure to Actionable Alerts sections to explain this trade-off more explicitly.

Thank you for this important observation. We have added clarifying sentences in both the Evaluation Protocol and Practical Implications sections. We now explain that the model-specific outbreak thresholds support within-model operational calibration, but make direct cross-model comparison of threshold-dependent metrics such as precision, recall, and F1 less straightforward. We also note that threshold-independent metrics such as ROC-AUC should therefore be interpreted alongside the alert metrics.

5. The statistical-significance analysis is useful and much clearer now. Still, the manuscript should make sure the reader understands that the differences between Prophet and the shortlisted competitors are generally modest, with significance appearing mainly for one-step absolute error comparisons. This is already partly stated, but a slightly more explicit caution in the Pairwise significance testing section and Discussion would help avoid over-interpretation.

Thank you for this helpful suggestion. We have added more explicit caution in the Pairwise significance testing section and Discussion. The revised text now clarifies that Prophet's advantage over the shortlisted competitors was generally modest, and that statistically significant separation appeared mainly for one-step absolute-error comparisons rather than consistently across horizons or loss functions.

6. The sensitivity-analysis results are interesting, especially the finding that removal of serotype variables improved some models and that a simpler non-seasonal SARIMAX outperformed the main seasonal specification. These findings are important and deserve a bit more interpretation in the Sensitivity and Uncertainty Analysis section and Discussion, because they suggest that some feature groups or baseline assumptions may not contribute uniformly across architectures.

Thank you for this helpful suggestion. We have added further interpretation in both the Sensitivity and Uncertainty Analysis section and the Discussion. We now clarify that the contribution of serotype variables was architecture-dependent, as their removal slightly improved MLR and more clearly improved attention-LSTM. We also discuss that the simpler non-seasonal SARIMAX specification outperformed the seasonal baseline, suggesting that fixed seasonal ARIMA terms may be restrictive when outbreak timing shifts across years. These revisions emphasize that feature groups and baseline assumptions should be evaluated by model architecture and forecast objective rather than assumed to improve all models uniformly.

7. The interpretability and causal-analysis sections add value to the manuscript, but the distinction between predictive usefulness and causal importance should be stated very carefully. The current text is already moving in the right direction, especially when discussing serotype centrality versus forecasting gain, and I encourage the authors to preserve this cautious wording. A small amount of polishing in Model Interpretability and Feature Importance and Causal Inference Analysis would further strengthen the paper.

Thank you for this helpful comment. We have polished the Model Interpretability and Feature Importance and Causal Inference Analysis sections to distinguish predictive usefulness from causal importance more carefully. We now clarify that interpretability results describe model reliance and predictive contribution rather than causal effects, and that PCMCI network centrality reflects statistical dependency within the fitted causal-discovery framework rather than definitive biological causation or guaranteed forecasting improvement.

8. The discussion is thoughtful and one of the stronger parts of the manuscript. The tactical-versus strategic framing is useful and helps explain why different models perform best for different objectives. I would only suggest some light language polishing in the Discussion and Conclusion, especially in places where the phrasing is slightly awkward or grammatically rough.

Thank you for this positive and helpful comment. We have lightly polished the Discussion and Conclusion to improve clarity, grammar, and flow while preserving the tactical-versus-strategic framing. We revised awkward phrasing, clarified model-specific interpretation, and made the limitation regarding external validation more precise.

9. The authors may consider citing a few recent related works to further strengthen the positioning of the manuscript within the current forecasting and mathematical-modeling literature, for example:

- https://doi.org/10.1016/j.cnsns.2025.109246
- https://doi.org/10.3329/ganit.v43i1.67858
- https://doi.org/10.48550/arXiv.2604.18642
- https://doi.org/10.1016/j.aej.2025.12.039

where relevant to the revised discussion and methodological context.

Thank you for this useful suggestion. We have added the suggested recent studies where relevant to strengthen the manuscript's positioning within the current forecasting and mathematical-modeling literature. The Bangladesh-focused dengue forecasting study was cited in the methodological context, the broader infectious-disease modelling studies were cited in the modelling motivation, and the dengue control/decision-support study was cited in the Discussion to distinguish forecasting from intervention-focused modelling.

10. Overall, this is a solid and improved manuscript with a relevant case study, a well-motivated framework, and a practically useful evaluation perspective. The remaining issues are mostly about wording, clarification, and slight moderation of some claims.

Response to Overall Comment:

Thank you for the positive assessment of the revised manuscript. We appreciate the reviewer's recognition of the case study, framework, and evaluation perspective. In response to the remaining comments, we have made targeted revisions to clarify the methodology, moderate generalization claims, improve interpretation of sensitivity and significance results, and polish the wording in the Discussion and Conclusion.
