# Supplementary material for: Tactical vs. strategic: An adaptable framework for horizon-dependent dengue forecasting using data-driven approaches with serotype and climate covariates in Bangladesh
Source: PLoS One. 2026 Aug 21;21(8):e0353069. doi: 10.1371/journal.pone.0353069 (PMC13496456; doi:10.1371/journal.pone.0353069)
Supplement: Supplementary file 7 [file pone.0353069.s015.pdf]

# Response Letter

## Manuscript ID PONE-D-26-03317

## Tactical vs. strategic: An adaptable framework for horizon-dependent dengue forecasting using data-driven approaches with serotype and climate covariates in Bangladesh

## PLOS ONE

### Response to Academic Editor

*Thank you for the opportunity to revise our manuscript. We have carefully addressed the comments and prepared a revised manuscript, a marked-up version, and a clean version for submission.*

### Response to Reviewer #1

*Thank you for handling and reading our manuscript. We have addressed all of your comments as follows:*

1. Could you please recheck Comment 1 carefully? It appears that this comment was not part of the current revision round, and there may have been a mismatch in the included comment. Please address the appropriate comment accordingly.

*Thank you for your meticulous observation. Actually, it was mistakenly placed. However, the question and the answer is included below.*

*''The interval coverage during the outbreak period appears to be consistently zero across all forecast horizons (Table 8). This requires an explanation.''*

*Thank you for pointing this out. We have added an explanation in the Sensitivity and Uncertainty Analysis section. We now clarify that the zero outbreak-period coverage in Table 8 means that none of the observed outbreak counts fell within TFT's predicted 80% intervals. The model still produced prediction intervals, but these intervals were below the observed outbreak magnitudes and shallow learning from dominant low dengue count period.*
