# Supplementary material for: Tactical vs. strategic: An adaptable framework for horizon-dependent dengue forecasting using data-driven approaches with serotype and climate covariates in Bangladesh
Source: PLoS One. 2026 Aug 21;21(8):e0353069. doi: 10.1371/journal.pone.0353069 (PMC13496456; doi:10.1371/journal.pone.0353069)
Supplement: Supplementary file 9 [file pone.0353069.s002.zip › S1_Fig/attention_profile_h6.pdf]

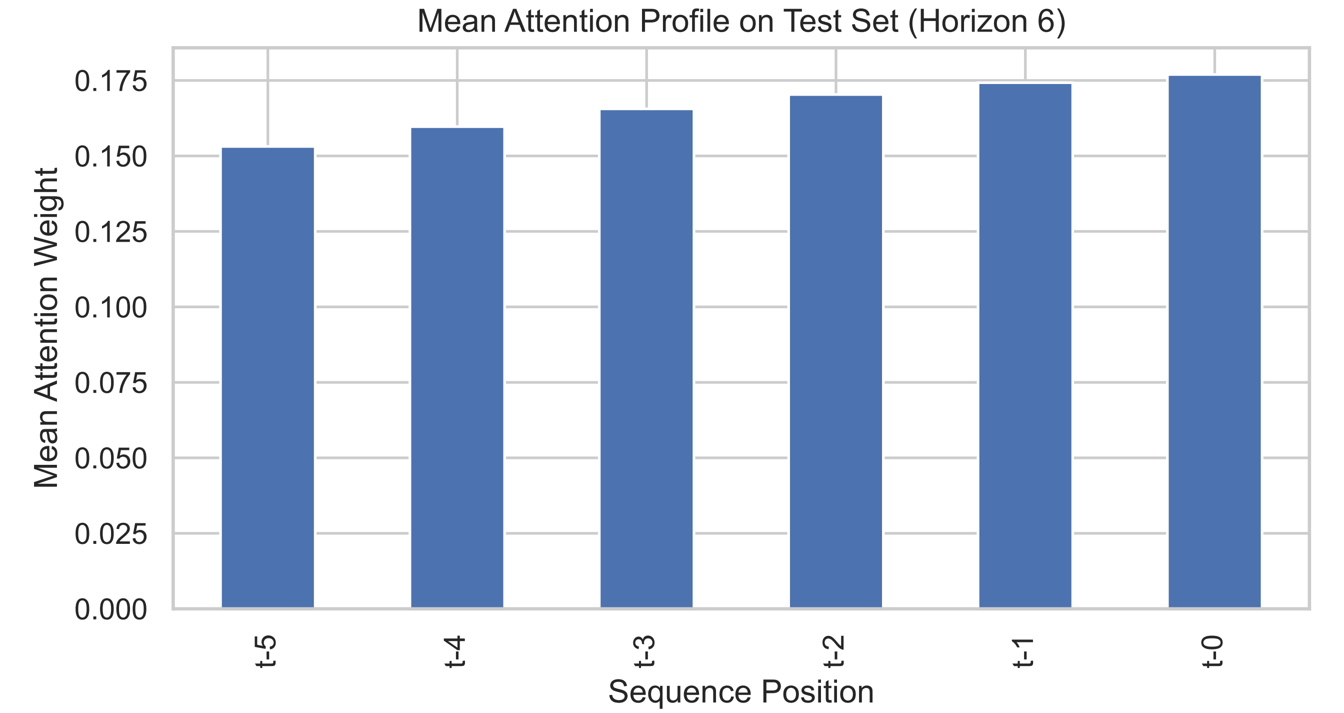
Mean Attention Profile on Test Set (Horizon 6)
Mean Attention Weight
0.000
0.025
0.050
0.075
0.100
0.125
0.150
0.175
t-5
t-4
t-3
t-2
t-1
t-0
Sequence Position
