# Supplementary material for: Tactical vs. strategic: An adaptable framework for horizon-dependent dengue forecasting using data-driven approaches with serotype and climate covariates in Bangladesh
Source: PLoS One. 2026 Aug 21;21(8):e0353069. doi: 10.1371/journal.pone.0353069 (PMC13496456; doi:10.1371/journal.pone.0353069)
Supplement: Supplementary file 9 [file pone.0353069.s002.zip › S1_Fig/permutation_importance_bar.pdf]

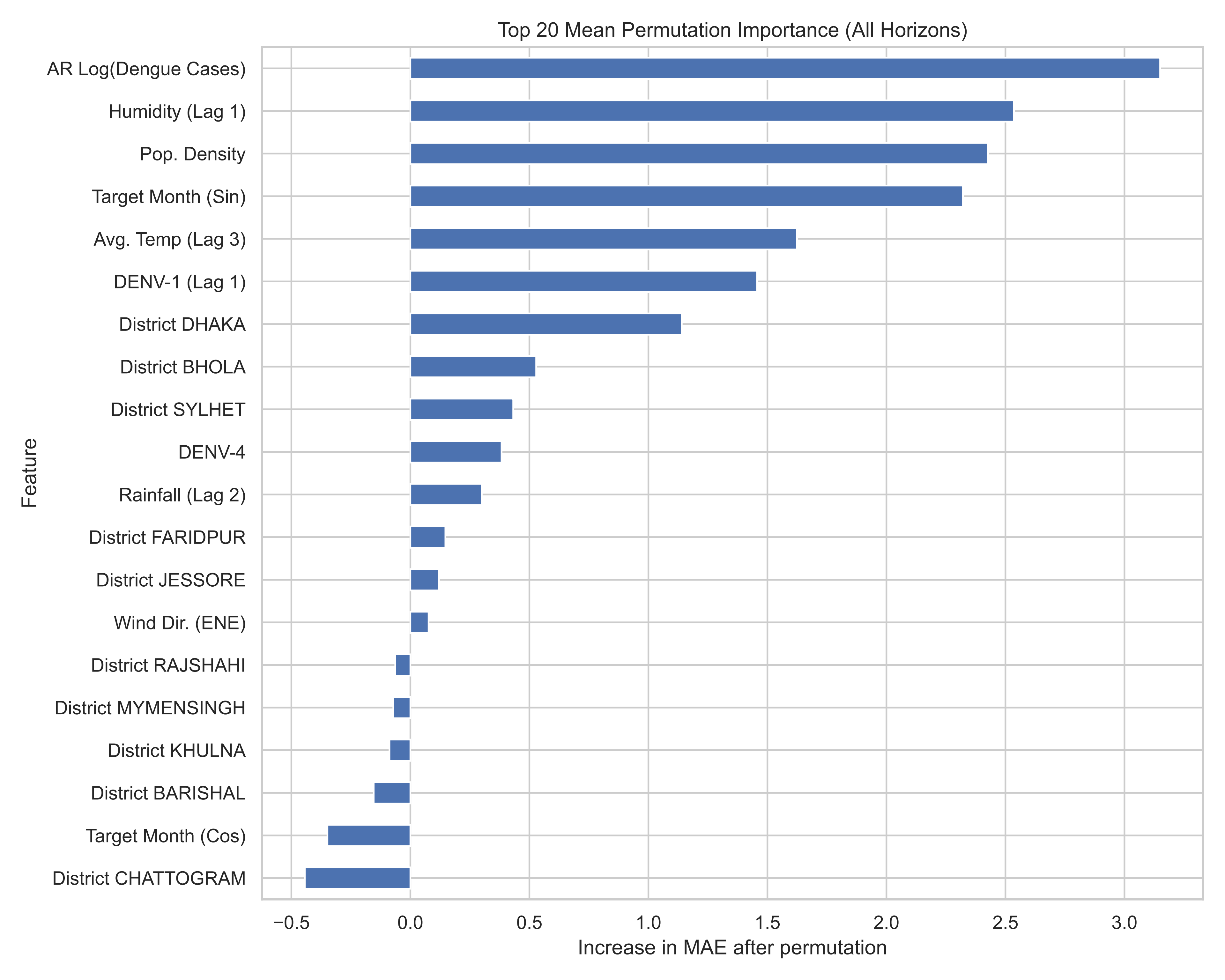

Top 20 Mean Permutation Importance (All Horizons)
AR Log(Dengue Cases)
Humidity (Lag 1)
Pop. Density
Target Month (Sin)
Avg. Temp (Lag 3)
DENV-1 (Lag 1)
District DHAKA
District BHOLA
District SYLHET
DENV-4
Rainfall (Lag 2)
District FARIDPUR
District JESSORE
Wind Dir. (ENE)
District RAJSHAHI
District MYMENSINGH
District KHULNA
District BARISHAL
Target Month (Cos)
District CHATTOGRAM
Feature
−0.5
0.0
0.5
1.0
1.5
2.0
2.5
3.0
Increase in MAE after permutation
