# Supplementary material for: Tactical vs. strategic: An adaptable framework for horizon-dependent dengue forecasting using data-driven approaches with serotype and climate covariates in Bangladesh
Source: PLoS One. 2026 Aug 21;21(8):e0353069. doi: 10.1371/journal.pone.0353069 (PMC13496456; doi:10.1371/journal.pone.0353069)
Supplement: Peer review history

# Peer review: Decision Letter 0

**DOI:** 10.1371/journal.pone.0353069.r001

**Authors:** Md. Kamrujjaman

17 Mar 2026

Dear Dr. Aziz,

Thank you for submitting your manuscript to PLOS ONE. After careful consideration, we feel that it has merit but does not fully meet PLOS ONE’s publication criteria as it currently stands. Therefore, we invite you to submit a revised version of the manuscript that addresses the points raised during the review process.

Please submit your revised manuscript by May 01 2026 11:59PM. If you will need more time than this to complete your revisions, please reply to this message or contact the journal office at plosone@plos.org . When you're ready to submit your revision, log on to https://www.editorialmanager.com/pone/ and select the 'Submissions Needing Revision' folder to locate your manuscript file.

- A letter that responds to each point raised by the academic editor and reviewer(s). You should upload this letter as a separate file labeled 'Response to Reviewers'.
- A marked-up copy of your manuscript that highlights changes made to the original version. You should upload this as a separate file labeled 'Revised Manuscript with Track Changes'.
- An unmarked version of your revised paper without tracked changes. You should upload this as a separate file labeled 'Manuscript'.

If you would like to make changes to your financial disclosure, please include your updated statement in your cover letter. Guidelines for resubmitting your figure files are available below the reviewer comments at the end of this letter.

If applicable, we recommend that you deposit your laboratory protocols in protocols.io to enhance the reproducibility of your results. Protocols.io assigns your protocol its own identifier (DOI) so that it can be cited independently in the future. For instructions see: https://journals.plos.org/plosone/s/submission-guidelines#loc-laboratory-protocols . Additionally, PLOS ONE offers an option for publishing peer-reviewed Lab Protocol articles, which describe protocols hosted on protocols.io. Read more information on sharing protocols at https://plos.org/protocols?utm_medium=editorial-email&utm_source=authorletters&utm_campaign=protocols .

We look forward to receiving your revised manuscript.

Kind regards,

Md. Kamrujjaman, Ph.D

Academic Editor

PLOS One

Journal Requirements:

When submitting your revision, we need you to address these additional requirements.

1. Please ensure that your manuscript meets PLOS ONE's style requirements, including those for file naming. The PLOS ONE style templates can be found at

https://journals.plos.org/plosone/s/file?id=wjVg/PLOSOne_formatting_sample_main_body.pdf and

https://journals.plos.org/plosone/s/file?id=ba62/PLOSOne_formatting_sample_title_authors_affiliations.pdf

2. Please note that PLOS One has specific guidelines on code sharing for submissions in which author-generated code underpins the findings in the manuscript. In these cases, we expect all author-generated code to be made available without restrictions upon publication of the work. Please review our guidelines at https://journals.plos.org/plosone/s/materials-and-software-sharing#loc-sharing-code and ensure that your code is shared in a way that follows best practice and facilitates reproducibility and reuse.

3. Please update your submission to use the PLOS LaTeX template. The template and more information on our requirements for LaTeX submissions can be found at http://journals.plos.org/plosone/s/latex.

4. Thank you for uploading your study's underlying data set. Unfortunately, the repository you have noted in your Data Availability statement does not qualify as an acceptable data repository according to PLOS's standards.

At this time, please upload the minimal data set necessary to replicate your study's findings to a stable, public repository (such as figshare or Dryad) and provide us with the relevant URLs, DOIs, or accession numbers that may be used to access these data. For a list of recommended repositories and additional information on PLOS standards for data deposition, please see https://journals.plos.org/plosone/s/recommended-repositories.

5. When completing the data availability statement of the submission form, you indicated that you will make your data available on acceptance. We strongly recommend all authors decide on a data sharing plan before acceptance, as the process can be lengthy and hold up publication timelines. Please note that, though access restrictions are acceptable now, your entire data will need to be made freely accessible if your manuscript is accepted for publication. This policy applies to all data except where public deposition would breach compliance with the protocol approved by your research ethics board. If you are unable to adhere to our open data policy, please kindly revise your statement to explain your reasoning and we will seek the editor's input on an exemption. Please be assured that, once you have provided your new statement, the assessment of your exemption will not hold up the peer review process.

6. Please amend either the abstract on the online submission form (via Edit Submission) or the abstract in the manuscript so that they are identical.

7. We note that Figure 1 in your submission contain map images which may be copyrighted. All PLOS content is published under the Creative Commons Attribution License (CC BY 4.0), which means that the manuscript, images, and Supporting Information files will be freely available online, and any third party is permitted to access, download, copy, distribute, and use these materials in any way, even commercially, with proper attribution. For these reasons, we cannot publish previously copyrighted maps or satellite images created using proprietary data, such as Google software (Google Maps, Street View, and Earth). For more information, see our copyright guidelines: http://journals.plos.org/plosone/s/licenses-and-copyright.

We require you to either (1) present written permission from the copyright holder to publish these figures specifically under the CC BY 4.0 license, or (2) remove the figures from your submission:

1. You may seek permission from the original copyright holder of Figure(s) [#] to publish the content specifically under the CC BY 4.0 license.

We recommend that you contact the original copyright holder with the Content Permission Form (http://journals.plos.org/plosone/s/file?id=7c09/content-permission-form.pdf) and the following text:

“I request permission for the open-access journal PLOS ONE to publish XXX under the Creative Commons Attribution License (CCAL) CC BY 4.0 (http://creativecommons.org/licenses/by/4.0/). Please be aware that this license allows unrestricted use and distribution, even commercially, by third parties. Please reply and provide explicit written permission to publish XXX under a CC BY license and complete the attached form.”

Please upload the completed Content Permission Form or other proof of granted permissions as an "Other" file with your submission.

In the figure caption of the copyrighted figure, please include the following text: “Reprinted from [ref] under a CC BY license, with permission from [name of publisher], original copyright [original copyright year].”

2. If you are unable to obtain permission from the original copyright holder to publish these figures under the CC BY 4.0 license or if the copyright holder’s requirements are incompatible with the CC BY 4.0 license, please either i) remove the figure or ii) supply a replacement figure that complies with the CC BY 4.0 license. Please check copyright information on all replacement figures and update the figure caption with source information. If applicable, please specify in the figure caption text when a figure is similar but not identical to the original image and is therefore for illustrative purposes only.

The following resources for replacing copyrighted map figures may be helpful:

USGS National Map Viewer (public domain): http://viewer.nationalmap.gov/viewer/

The Gateway to Astronaut Photography of Earth (public domain): http://eol.jsc.nasa.gov/sseop/clickmap/

Maps at the CIA (public domain): https://www.cia.gov/library/publications/the-world-factbook/index.html and https://www.cia.gov/library/publications/cia-maps-publications/index.html

NASA Earth Observatory (public domain): http://earthobservatory.nasa.gov/

Landsat: http://landsat.visibleearth.nasa.gov/

USGS EROS (Earth Resources Observatory and Science (EROS) Center) (public domain): http://eros.usgs.gov/#

Natural Earth (public domain): http://www.naturalearthdata.com/

8. Please ensure that you refer to Figures 2, 4 and 11 in your text as, if accepted, production will need this reference to link the reader to the figure.

9. We note you have included a table to which you do not refer in the text of your manuscript. Please ensure that you refer to Tables 1 - 4 in your text; if accepted, production will need this reference to link the reader to the Table.

10. If the reviewer comments include a recommendation to cite specific previously published works, please review and evaluate these publications to determine whether they are relevant and should be cited. There is no requirement to cite these works unless the editor has indicated otherwise.

[Note: HTML markup is below. Please do not edit.]

Reviewers' comments:

Reviewer's Responses to Questions

**Comments to the Author**

1. Is the manuscript technically sound, and do the data support the conclusions?

Reviewer #1: Yes

Reviewer #2: Partly

**********

2. Has the statistical analysis been performed appropriately and rigorously?

Reviewer #1: Yes

Reviewer #2: Yes

**********

3. Have the authors made all data underlying the findings in their manuscript fully available?

The PLOS Data policy

Reviewer #1: No

Reviewer #2: Yes

**********

4. Is the manuscript presented in an intelligible fashion and written in standard English?

Reviewer #1: Yes

Reviewer #2: Yes

**********

Reviewer #1: Minor Comments:

1. Please clarify how the “dominant” label is determined for each year by defining what “type” refers to and stating the exact rule used to select the dominant category (e.g., the category with the highest value or frequency), with a brief example if possible.

2. There are inconsistencies in formatting, quotation usage, and punctuation around terms and names. Please correct this type of typographical inconsistency consistently throughout the manuscript.

3. In Table 2, please add adequate spacing between the Unit and Domain-Based Class columns to improve readability and prevent the entries from appearing merged.

4. Please revise punctuation for figure citations and ensure consistent use of parentheses (e.g., write “…with excess zeros (Fig. 3b)” rather than leaving the figure reference outside the sentence structure).

5. Please include the missing figure numbers and cite them consistently in parentheses throughout the manuscript.

6. Please add proper spacing for consistency, e.g., AvgTemp_lag_3 (r = 0.372).

7. Use a semicolon when listing definitions so each item is clearly separated (e.g.,$\tau$: forecast horizon (steps ahead), with $\tau\in\{1,\ldots,6\}$.).

8.Quotation marks are used inconsistently; please replace them with proper LaTeX quotes (e.g., ``Normal'' instead of "Normal") throughout the manuscript.

Major Comments:

1. In the figures, key text elements (e.g., axis tick labels, legend entries, and titles) are too small and difficult to read. Please increase the font sizes across all plots to ensure clear visibility and consistent readability.

2. Figures 5 and 6 are currently presented in grayscale; using distinct colors would make the plotted elements easier to distinguish and improve overall readability.

3. Add uncertainty and calibration results for the quantile-based TFT using coverage and pinball loss, ideally reported separately for Normal and Outbreak periods.

4. Using a time-series aware significance test, such as a block bootstrap or rolling-window paired tests, would better account for autocorrelation in forecast errors.

5. Presenting feature-group ablation experiments (climate-only, serotype-only, climate plus serotype, then adding demography and case-lags) would clarify which inputs drive performance at each horizon.

6. Treating outbreak prediction as a separate early-warning task and summarizing it with precision, recall, F1, or ROC-AUC would strengthen the ``actionable alerts" discussion.

Reviewer #2: The manuscript addresses an important public health problem and presents a useful horizon-dependent comparison of dengue forecasting models, which is a clear strength. However, the current version needs substantial revision before it can be considered further. In particular, the preprocessing pipeline raises a serious concern about possible data leakage, the handling of district-level panel data versus single-series modeling is not sufficiently clear, and the construction and encoding of serotype-related covariates need better justification and explanation. In addition, the statistical comparison framework should better account for temporal dependence, several figure/table placeholders remain unresolved, and the reproducibility details are still incomplete. The practical early-warning discussion is promising, but it would be stronger if supported by explicit outbreak-detection metrics. Overall, the study has potential, but the methodology and reporting need to be clarified and strengthened.

**********

what does this mean? ). If published, this will include your full peer review and any attached files.

If you choose “no”, your identity will remain anonymous but your review may still be made public.

**Do you want your identity to be public for this peer review?**   For information about this choice, including consent withdrawal, please see our Privacy Policy

Reviewer #1: No

Reviewer #2: **Yes:**  Nuzhat Nuari Khan Rivu

**********

[NOTE: If reviewer comments were submitted as an attachment file, they will be attached to this email and accessible via the submission site. Please log into your account, locate the manuscript record, and check for the action link "View Attachments". If this link does not appear, there are no attachment files.]

To ensure your figures meet our technical requirements, please review our figure guidelines: https://journals.plos.org/plosone/s/figures

You may also use PLOS’s free figure tool, NAAS, to help you prepare publication quality figures: https://journals.plos.org/plosone/s/figures#loc-tools-for-figure-preparation.

NAAS will assess whether your figures meet our technical requirements by comparing each figure against our figure specifications.

Attachment Submitted filename: recommendation.pdf

Attachment Submitted filename: document.pdf

# Author comment: Author response to Decision Letter 1

**DOI:** 10.1371/journal.pone.0353069.r002

22 Apr 2026

Response to Academic Editor

Please ensure that your manuscript meets PLOS ONE’s style requirements, including those for file naming.

Thank you for this reminder. We have revised the manuscript and associated submission files to comply with PLOS ONE style requirements, including formatting, structure, figure/table presentation, and file naming conventions. We have carefully checked the revised submission to ensure consistency with the journal guidelines.

Please ensure that your code is shared in accordance with PLOS ONE guidelines and is available without restrictions upon publication.

Thank you for this important requirement. We have ensured that our code sharing fully complies with PLOS ONE’s guidelines for transparency and reproducibility.

All author-generated code has been made publicly available without restriction via an archived repository with a DOI:

Code repository: https://doi.org/10.5281/zenodo.19589534

The repository includes:

Full training scripts for all models (e.g., SARIMAX, TFT, and deep learning models)

Complete preprocessing pipeline

Evaluation and analysis scripts

Hyperparameter configurations

Environment specifications, including package versions and Python setup

To address data sharing constraints, meteorological data obtained from the Bangladesh Meteorological Department cannot be publicly distributed due to access restrictions. To ensure reproducibility despite this limitation, we have provided a synthetic dataset that preserves the structure and statistical characteristics of the original data, allowing all code components to be executed and validated. The shared materials therefore enable full reproducibility of the workflow, including preprocessing, model training, and evaluation, in accordance with best practices.

Please update your submission to use the PLOS LaTeX template.

Thank you for this instruction. We have updated the manuscript using the PLOS LaTeX template and revised the submission files accordingly to align with the journal’s LaTeX formatting requirements.

The repository noted in the Data Availability statement does not qualify as an acceptable repository. Please upload the minimal dataset necessary to replicate the study to a stable public repository.

Thank you for highlighting this requirement. We have revised our data sharing approach to comply with PLOS ONE’s data availability standards. We have deposited the materials in a public, stable repository with a DOI:

Repository: https://doi.org/10.5281/zenodo.19589534

Due to access restrictions, the meteorological data obtained from the Bangladesh Meteorological Department cannot be publicly shared. However, to ensure reproducibility, we have provided a synthetic dataset that preserves the structure and statistical properties of the original data, along with all necessary preprocessing and modeling scripts.

This dataset, together with the shared code and documentation, enables replication of the full analytical workflow, including preprocessing, model training, and evaluation.

Please revise the data availability statement and clarify your data sharing plan, noting that all data must be freely accessible upon publication unless an exemption applies.

Thank you for this clarification. We have revised our Data Availability Statement to provide a clear and complete data sharing plan in line with PLOS ONE requirements.

All code, documentation, and a reproducible dataset have already been made publicly available in a stable repository with a DOI:

Repository: https://doi.org/10.5281/zenodo.19589534

Due to third-party restrictions, the meteorological data obtained from the Bangladesh Meteorological Department cannot be made publicly available, as redistribution is not permitted under the data provider’s terms.

To ensure reproducibility, we have provided a synthetic dataset that preserves the structure and statistical properties of the original data, along with full preprocessing, modeling, and evaluation code. This allows the complete workflow to be replicated without access to the restricted data.

We respectfully request that this be considered as a valid case for restricted data exemption, and we have updated the Data Availability Statement accordingly to explain these constraints.

Please amend either the abstract on the online submission form or the abstract in the manuscript so that they are identical.

Thank you for noting this discrepancy. We have revised the abstract so that the version in the manuscript and the version entered in the online submission system are now identical.

Figure 1 may contain copyrighted map images. Please provide permission or replace the figure with CC BY–compatible content.

Thank you for raising this important point. We confirm that Figure 1 does not use proprietary sources such as Google Maps or other restricted platforms.

The map is based on administrative boundary data obtained from the Humanitarian Data Exchange (Bangladesh Subnational Administrative Boundaries dataset), which is derived from open and publicly available sources. We have verified that the dataset is compatible with open-use requirements.

To ensure full compliance with PLOS ONE’s CC BY 4.0 licensing policy, we have:

Clearly cited the data source in the figure caption

Updated the caption to reflect that the map is created from open administrative boundary data

Confirmed that no copyrighted or proprietary basemaps are used

The revised figure and caption now comply with the journal’s licensing requirements.

Please ensure that you refer to Figures 2, 4 and 11 in your text as, if accepted, production will need this reference to link the reader to the figure..

Thank you for this observation. Following revisions to the analysis and figures, we have updated the manuscript to ensure that all figures are correctly numbered and consistently referenced in the text.

Specifically:

Figures previously labeled as 2 and 4 have been renumbered and updated, and are now properly cited in the revised manuscript

Figure 11 has been removed as part of the revised analysis

All figure references have been carefully checked to ensure consistency and correct linkage throughout the manuscript.

We note you have included tables that were not properly referred to in the text. Please ensure that Tables 1-4 are referred to in the manuscript.

Thank you for this observation. We have carefully revised the manuscript to ensure that all tables retained in the main text are explicitly referred to and discussed in the appropriate places.

In addition, to improve clarity and reduce length, we reorganized the tabular material during revision. Non-essential descriptive tables were moved to the Supporting Information or summarized in the text, while only the most relevant tables were retained in the main manuscript. All remaining tables are now consistently numbered and properly cited in the revised manuscript.

If the reviewer comments include a recommendation to cite specific previously published works, please evaluate whether they are relevant and should be cited.

Thank you for this clarification. We carefully reviewed the publications recommended by the reviewers and evaluated their relevance to the scope, methodology, and objectives of our study. Where appropriate, we have incorporated relevant citations into the revised manuscript. For works that were not directly aligned with the present study, we did not add citations.

Response to Reviewer #1

Thank you for handling and reading our manuscript. We have addressed all of your comments as follows:

##Minor Comments

Please clarify how the “dominant” label is determined for each year by defining what “type” refers to and stating the exact rule used to select the dominant category (e.g., the category with the highest value or frequency), with a brief example if possible.

Thank you for this suggestion. We have clarified the definition of the dominant serotype variable in the revised manuscript. Here, “type” refers to the dengue serotype category (DENV-1 to DENV-4). For each year, the dominant label was defined as the serotype with the highest annual value/frequency in the compiled serotype data, and that label was assigned to the monthly observations for that year. For example, if DENV-2 was the most frequent serotype in a given year, the dominant label for that year was recorded as DENV-2. We have added this explanation to the manuscript to make the construction of the variable explicit and reproducible.

There are inconsistencies in formatting, quotation usage, and punctuation around terms and names. Please correct this type of typographical inconsistency consistently throughout the manuscript.

Thank you for this careful observation. We have performed a thorough proofreading of the revised manuscript and corrected typographical inconsistencies throughout. In particular, we standardized formatting, punctuation, capitalization, quotation usage, and the presentation of terms, names, and figure/table references to improve consistency and readability across the manuscript.

In Table 2, please add adequate spacing between the Unit and Domain-Based Class columns to improve readability and prevent the entries from appearing merged.

Thank you for this suggestion. To improve readability and overall manuscript clarity, Table 2 has been moved to the Supporting Information as part of the revised manuscript structure. In the supplementary version, we have also improved the formatting and spacing, including clearer separation between the Unit and Domain-Based Class columns.

Please revise punctuation for figure citations and ensure consistent use of parentheses (e.g., write “…with excess zeros (Fig. 3b)” rather than leaving the figure reference outside the sentence structure).

Thank you for pointing this out. We have revised the manuscript to ensure that figure citations are punctuated consistently and integrated properly within sentence structure, including the consistent use of parentheses in the PLOS style (e.g., “(Fig. X)”). All figure references were reviewed and corrected throughout the manuscript for consistency.

Please include the missing figure numbers and cite them consistently in parentheses throughout the manuscript.

Thank you for this observation. We have carefully revised the manuscript to resolve the missing figure numbers and to ensure that all figures are now cited consistently in parentheses throughout the text. We also checked figure numbering globally after revision so that all in-text references match the final figure order.

Please add proper spacing for consistency, e.g., AvgTemp_lag_3 (r = 0.372).

Thank you for this suggestion. We have revised the manuscript to improve formatting and readability by standardizing the presentation of variable names and statistical notation. For example, terms such as AvgTemp_lag_3 (r = 0.372) have been reformatted to a clearer style (e.g., Avg. Temp (lag 3)), and spacing has been made consistent throughout the manuscript.

Use a semicolon when listing definitions so each item is clearly separated (e.g.,$\tau$: forecast horizon (steps ahead), with $\tau\in\{1,\ldots,6\}$.).

Thank you for this suggestion. We revised the presentation of notation and symbol definitions to improve clarity and consistency. In the revised manuscript, notation is now presented more clearly in the nomenclature table and related mathematical descriptions, so that individual definitions are separated and easier to read. We also standardized punctuation and formatting in notation-related text throughout the manuscript.

Quotation marks are used inconsistently; please replace them with proper LaTeX quotes (e.g., ``Normal'' instead of "Normal") throughout the manuscript.

Thank you for this observation. We have revised the manuscript to standardize quotation mark usage throughout and replaced inconsistent quotation formatting with proper LaTeX quotation style where applicable. This was checked across the full manuscript to improve consistency and presentation.

##Major Comments:

In the figures, key text elements (e.g., axis tick labels, legend entries, and titles) are too small and difficult to read. Please increase the font sizes across all plots to ensure clear visibility and consistent readability.

Thank you for this important observation. We have revised all figures in the manuscript to improve readability by increasing font sizes for axis labels, tick labels, legends, and titles. We also ensured consistent formatting across all plots so that text elements are clearly visible both on screen and in print.

Figures 5 and 6 are currently presented in grayscale; using distinct colors would make the plotted elements easier to distinguish and improve overall readability.

Thank you for this suggestion. We revised the figure set to improve visual clarity and distinguishability. In the revised manuscript, the relevant figures were updated to use distinct colors instead of grayscale where appropriate. Because the figure set was reorganized during revision, the original numbering also changed: the figure previously cited as Figure 5 was renumbered, and the original Figure 6 was removed as part of the revised analysis. More broadly, we applied improved color formatting across the figures to enhance readability throughout the manuscript.

Add uncertainty and calibration results for the quantile-based TFT using coverage and pinball loss, ideally reported separately for Normal and Outbreak periods.

Thank you for this valuable suggestion. We have expanded the revised manuscript to include an explicit uncertainty and calibration analysis for the quantile-based TFT model. Specifically, we now report interval coverage, mean interval width, and quantile pinball loss, and we present these results separately for Normal and Outbreak periods across forecast horizons. This addition is included in the revised Results section(Uncertainty and calibration analysis) and provides a clearer assessment of TFT’s probabilistic performance and calibration under both routine and epidemic conditions. (Table 8: TFT regime-wise 80% interval coverage, mean interval width, and pinball loss by forecast horizon.)

Using a time-series aware significance test, such as a block bootstrap or rolling-window paired tests, would better account for autocorrelation in forecast errors.

Thank you for this thoughtful suggestion. We agree that statistical comparison of forecasting models should account for temporal dependence in forecast errors. In the revised manuscript, we addressed this concern by adopting a time-series-aware Diebold-Mariano testing framework with the Harvey-Leybourne-Newbold small-sample correction and Newey-West long-run variance estimation, with lag selected as a function of forecast horizon, presented in the result section(Pairwise significance testing); Table 10. Pairwise Diebold–Mariano significance testing comparing Prophet with shortlisted competing models across horizons.

We therefore revised the significance-analysis section to use a temporally appropriate comparison procedure, even though we did not implement the specific alternatives suggested by the reviewer (e.g., block bootstrap or rolling-window paired tests). This updated procedure is now described in the manuscript and the corresponding results are reported in the revised Results section.

Presenting feature-group ablation experiments (climate-only, serotype-only, climate plus serotype, then adding demography and case-lags) would clarify which inputs drive performance at each horizon.

Thank you for this constructive suggestion. We agree that feature-group comparisons can help clarify which inputs drive forecasting performance across horizons. In the revised manuscript, we addressed this issue through a feature-group sensitivity analysis based on one-factor-at-a-time ablation, using the same preprocessed panel, split structure, and horizon definitions as in the main experiments. Specifically, we compared the retained full specification against variants with climate variables, serotype variables, temporal variables, or population density removed, depending on model class. This allowed us to assess how the

Attachment Submitted filename: Response to Reviewers.pdf

# Peer review: Decision Letter 1

**DOI:** 10.1371/journal.pone.0353069.r003

**Authors:** Md. Kamrujjaman

30 Apr 2026

Dear Dr. Aziz,

Thank you for submitting your manuscript to PLOS ONE. After careful consideration, we feel that it has merit but does not fully meet PLOS ONE’s publication criteria as it currently stands. Therefore, we invite you to submit a revised version of the manuscript that addresses the points raised during the review process.

Please submit your revised manuscript by Jun 14 2026 11:59PM. If you will need more time than this to complete your revisions, please reply to this message or contact the journal office at plosone@plos.org . When you're ready to submit your revision, log on to https://www.editorialmanager.com/pone/ and select the 'Submissions Needing Revision' folder to locate your manuscript file.

- A letter that responds to each point raised by the academic editor and reviewer(s). You should upload this letter as a separate file labeled 'Response to Reviewers'.
- A marked-up copy of your manuscript that highlights changes made to the original version. You should upload this as a separate file labeled 'Revised Manuscript with Track Changes'.
- An unmarked version of your revised paper without tracked changes. You should upload this as a separate file labeled 'Manuscript'.

If you would like to make changes to your financial disclosure, please include your updated statement in your cover letter. Guidelines for resubmitting your figure files are available below the reviewer comments at the end of this letter.

If applicable, we recommend that you deposit your laboratory protocols in protocols.io to enhance the reproducibility of your results. Protocols.io assigns your protocol its own identifier (DOI) so that it can be cited independently in the future. For instructions see: https://journals.plos.org/plosone/s/submission-guidelines#loc-laboratory-protocols . Additionally, PLOS ONE offers an option for publishing peer-reviewed Lab Protocol articles, which describe protocols hosted on protocols.io. Read more information on sharing protocols at https://plos.org/protocols?utm_medium=editorial-email&utm_source=authorletters&utm_campaign=protocols .

As the corresponding author, your ORCID iD is verified in the submission system and will appear in the published article. PLOS supports the use of ORCID, and we encourage all coauthors to register for an ORCID iD and use it as well. Please encourage your coauthors to verify their ORCID iD within the submission system before final acceptance, as unverified ORCID iDs will not appear in the published article. *Only*   the individual author can complete the verification step; PLOS staff *cannot*   verify ORCID iDs on behalf of authors.

We look forward to receiving your revised manuscript.

Kind regards,

Md. Kamrujjaman, Ph.D

Academic Editor

PLOS One

Journal Requirements:

1. If the reviewer comments include a recommendation to cite specific previously published works, please review and evaluate these publications to determine whether they are relevant and should be cited. There is no requirement to cite these works unless the editor has indicated otherwise.

2. Please review your reference list to ensure that it is complete and correct. If you have cited papers that have been retracted, please include the rationale for doing so in the manuscript text, or remove these references and replace them with relevant current references. Any changes to the reference list should be mentioned in the rebuttal letter that accompanies your revised manuscript. If you need to cite a retracted article, indicate the article’s retracted status in the References list and also include a citation and full reference for the retraction notice.

[Note: HTML markup is below. Please do not edit.]

Reviewer's Responses to Questions

**Comments to the Author**

Reviewer #1: (No Response)

Reviewer #2: All comments have been addressed

**********

2. Is the manuscript technically sound, and do the data support the conclusions?

Reviewer #1: (No Response)

Reviewer #2: Yes

**********

3. Has the statistical analysis been performed appropriately and rigorously?

Reviewer #1: (No Response)

Reviewer #2: Yes

**********

4. Have the authors made all data underlying the findings in their manuscript fully available?

The PLOS Data policy

Reviewer #1: (No Response)

Reviewer #2: Yes

**********

5. Is the manuscript presented in an intelligible fashion and written in standard English?

Reviewer #1: (No Response)

Reviewer #2: Yes

**********

Reviewer #1: (No Response)

Reviewer #2: (No Response)

**********

what does this mean? ). If published, this will include your full peer review and any attached files.

If you choose “no”, your identity will remain anonymous but your review may still be made public.

**Do you want your identity to be public for this peer review?**   For information about this choice, including consent withdrawal, please see our Privacy Policy

Reviewer #1: No

Reviewer #2: **Yes:**  Nuzhat Nuari Khan Rivu

**********

[NOTE: If reviewer comments were submitted as an attachment file, they will be attached to this email and accessible via the submission site. Please log into your account, locate the manuscript record, and check for the action link "View Attachments". If this link does not appear, there are no attachment files.]

To ensure your figures meet our technical requirements, please review our figure guidelines: https://journals.plos.org/plosone/s/figures

You may also use PLOS’s free figure tool, NAAS, to help you prepare publication quality figures: https://journals.plos.org/plosone/s/figures#loc-tools-for-figure-preparation.

NAAS will assess whether your figures meet our technical requirements by comparing each figure against our figure specifications.

Attachment Submitted filename: document.pdf

Attachment Submitted filename: recommendations.pdf

# Author comment: Author response to Decision Letter 2

**DOI:** 10.1371/journal.pone.0353069.r004

8 May 2026

Response Letter

Manuscript ID PONE-D-26-03317

Tactical vs. strategic: An adaptable framework for horizon-dependent dengue forecasting using data-driven approaches with serotype and climate covariates in Bangladesh

PLOS ONE

Response to Academic Editor

Thank you for the opportunity to revise our manuscript. We have carefully addressed the remaining comments and prepared a revised manuscript, a marked-up version, and a clean version for submission. We also reviewed the reference list, added the relevant recommended studies where appropriate, and checked the manuscript for clarity, consistency, and moderated claims. The specific changes are described point-by-point below.

Response to Reviewer #1

Thank you for handling and reading our manuscript. We have addressed all of your comments as follows:

1. Please clarify how the “dominant” label is determined for each year by defining what “type” refers to and stating the exact rule used to select the dominant category (e.g., the category with the highest value or frequency), with a brief example if possible.

Thank you for pointing this out. We have added an explanation in the Sensitivity and Uncertainty Analysis section. We now clarify that the zero outbreak-period coverage in Table 8 means that none of the observed outbreak counts fell within TFT’s predicted 80% intervals. The model still produced prediction intervals, but these intervals were systematically below the observed outbreak magnitudes, indicating poor calibration and underestimation of the upper tail during rare epidemic surges.

2. It is recommended to cite the following studies:

• https://doi.org/10.1371/journal.pone.0342764

• https://doi.org/10.26855/abr.2022.12.001

• https://doi.org/10.1038/s41598-025-28135-x

• https://doi.org/10.4236/am.2022.1310053

Thank you for the recommendation. We have cited the suggested studies in the Introduction to strengthen the manuscript’s positioning within the broader infectious-disease modelling literature. These studies are used as methodological background for predictive, stochastic, and mathematical modelling approaches relevant to epidemic assessment, resource allocation, and intervention planning.

Response to Reviewer #2

Thank you for handling and reading our manuscript. We have addressed all of your comments as follows:

1. The manuscript is now generally well structured, but the claim of a “generalizable framework” in the title and discussion should still be stated a little more cautiously. The framework is promising and well-motivated, but the present study demonstrates it on one country case study with one specific panel structure and one epidemic context. It may be better to describe it as a transferable or adaptable framework unless broader validation is provided. This point is most relevant in the Title, Methodological Framework & Case Study Setup, Discussion, and Conclusion.

Thank you for this helpful suggestion. We agree that “generalizable framework” overstated the scope of validation, as the present study is demonstrated using one country-level case study, one district-panel structure, and one epidemic context. We have therefore revised the title and relevant wording throughout the manuscript to describe the approach as an “adaptable framework” rather than a “generalizable framework.” We also added cautious wording in the Methodological Framework, Discussion, and Conclusion to clarify that broader transferability would require external validation in other geographical and epidemiological settings.

2. The treatment of serotype information is interesting and potentially novel, but the manuscript would benefit from slightly clearer explanation of how the annual “dominant serotype” variable was constructed and how this annual information aligns with monthly forecasting targets. Since the final selected feature set includes lagged serotype variables and the discussion emphasizes serotype importance, a short clarification in Dataset Development for Comprehensive Analysis and Feature Engineering would improve reproducibility.

Thank you for this helpful comment. We have clarified how the annual serotype variables were aligned with monthly forecasting targets. Specifically, we now state that annual DENV-I--DENV-IV percentage values were assigned to all monthly observations within the corresponding year, and that the dominant serotype variable was defined as the serotype with the highest annual percentage value for that year. We also clarified in the Feature Engineering section that lagged serotype variables were generated after this annual-to-monthly alignment. This revision improves reproducibility and clarifies how annual serotype surveillance information was incorporated into the monthly forecasting framework.

3. The treatment of serotype information is interesting and potentially novel, but the manuscript would benefit from slightly clearer explanation of how the annual “dominant serotype” variable was constructed and how this annual information aligns with monthly forecasting targets. Since the final selected feature set includes lagged serotype variables and the discussion emphasizes serotype importance, a short clarification in Dataset Development for Comprehensive Analysis and Feature Engineering would improve reproducibility.

Thank you for this helpful suggestion. We have revised the Feature Selection section to clarify the final manual selection step. We now explain that SelectKBest was used as a screening step, and that final predictors were selected from the screened set by prioritizing target relevance, low redundancy after correlation and VIF filtering, temporal availability at the source forecasting month, and representation of key epidemiological, climatic, temporal, and demographic domains

4. The evaluation design is one of the strong parts of the paper, especially the separation of overall, per-horizon, regime-wise, and outbreak-detection performance. However, because the outbreak thresholds are model-specific in Table 9, the comparison of alert metrics across models becomes a little harder to interpret directly. I suggest adding one sentence in the Evaluation Protocol and Practical Implications: From Magnitude Failure to Actionable Alerts sections to explain this trade-off more explicitly.

Thank you for this important observation. We have added clarifying sentences in both the Evaluation Protocol and Practical Implications sections. We now explain that the model-specific outbreak thresholds support within-model operational calibration, but make direct cross-model comparison of threshold-dependent metrics such as precision, recall, and F1 less straightforward. We also note that threshold-independent metrics such as ROC-AUC should therefore be interpreted alongside the alert metrics.

5. The statistical-significance analysis is useful and much clearer now. Still, the manuscript should make sure the reader understands that the differences between Prophet and the shortlisted competitors are generally modest, with significance appearing mainly for one-step absolute error comparisons. This is already partly stated, but a slightly more explicit caution in the Pairwise significance testing section and Discussion would help avoid over-interpretation.

Thank you for this helpful suggestion. We have added more explicit caution in the Pairwise significance testing section and Discussion. The revised text now clarifies that Prophet’s advantage over the shortlisted competitors was generally modest, and that statistically significant separation appeared mainly for one-step absolute-error comparisons rather than consistently across horizons or loss functions.

6. The sensitivity-analysis results are interesting, especially the finding that removal of serotype variables improved some models and that a simpler non-seasonal SARIMAX outperformed the main seasonal specification. These findings are important and deserve a bit more interpretation in the Sensitivity and Uncertainty Analysis section and Discussion, because they suggest that some feature groups or baseline assumptions may not contribute uniformly across architectures.

Thank you for this helpful suggestion. We have added further interpretation in both the Sensitivity and Uncertainty Analysis section and the Discussion. We now clarify that the contribution of serotype variables was architecture-dependent, as their removal slightly improved MLR and more clearly improved attention-LSTM. We also discuss that the simpler non-seasonal SARIMAX specification outperformed the seasonal baseline, suggesting that fixed seasonal ARIMA terms may be restrictive when outbreak timing shifts across years. These revisions emphasize that feature groups and baseline assumptions should be evaluated by model architecture and forecast objective rather than assumed to improve all models uniformly.

7. The interpretability and causal-analysis sections add value to the manuscript, but the distinction between predictive usefulness and causal importance should be stated very carefully. The current text is already moving in the right direction, especially when discussing serotype centrality versus forecasting gain, and I encourage the authors to preserve this cautious wording. A small amount of polishing in Model Interpretability and Feature Importance and Causal Inference Analysis would further strengthen the paper.

Thank you for this helpful comment. We have polished the Model Interpretability and Feature Importance and Causal Inference Analysis sections to distinguish predictive usefulness from causal importance more carefully. We now clarify that interpretability results describe model reliance and predictive contribution rather than causal effects, and that PCMCI network centrality reflects statistical dependency within the fitted causal-discovery framework rather than definitive biological causation or guaranteed forecasting improvement.

8. The discussion is thoughtful and one of the stronger parts of the manuscript. The tactical-versus strategic framing is useful and helps explain why different models perform best for different objectives. I would only suggest some light language polishing in the Discussion and Conclusion, especially in places where the phrasing is slightly awkward or grammatically rough.

Thank you for this positive and helpful comment. We have lightly polished the Discussion and Conclusion to improve clarity, grammar, and flow while preserving the tactical-versus-strategic framing. We revised awkward phrasing, clarified model-specific interpretation, and made the limitation regarding external validation more precise.

9. The authors may consider citing a few recent related works to further strengthen the positioning of the manuscript within the current forecasting and mathematical-modeling literature, for example:

• https://doi.org/10.1016/j.cnsns.2025.109246

• https://doi.org/10.3329/ganit.v43i1.67858

• https://doi.org/10.48550/arXiv.2604.18642

• https://doi.org/10.1016/j.aej.2025.12.039

where relevant to the revised discussion and methodological context.

Thank you for this useful suggestion. We have added the suggested recent studies where relevant to strengthen the manuscript’s positioning within the current forecasting and mathematical-modeling literature. The Bangladesh-focused dengue forecasting study was cited in the methodological context, the broader infectious-disease modelling studies were cited in the modelling motivation, and the dengue control/decision-support study was cited in the Discussion to distinguish forecasting from intervention-focused modelling.

10. Overall, this is a solid and improved manuscript with a relevant case study, a well-motivated framework, and a practically useful evaluation perspective. The remaining issues are mostly about wording, clarification, and slight moderation of some claims.

Response to Overall Comment:

Thank you for the positive assessment of the revised manuscript. We appreciate the reviewer’s recognition of the case study, framework, and evaluation perspective. In response to the remaining comments, we have made targeted revisions to clarify the methodology, moderate generalization claims, improve interpretation of sensitivity and significance results, and polish the wording in the Discussion and Conclusion.

Attachment Submitted filename: Response_to_Reviewers_auresp_2.pdf

# Peer review: Decision Letter 2

**DOI:** 10.1371/journal.pone.0353069.r005

**Authors:** Md. Kamrujjaman

10 Jun 2026

Dear Dr. Aziz,

Thank you for submitting your manuscript to PLOS ONE. After careful consideration, we feel that it has merit but does not fully meet PLOS ONE’s publication criteria as it currently stands. Therefore, we invite you to submit a revised version of the manuscript that addresses the points raised during the review process.

Please submit your revised manuscript by Jul 25 2026 11:59PM. If you will need more time than this to complete your revisions, please reply to this message or contact the journal office at plosone@plos.org . When you're ready to submit your revision, log on to https://www.editorialmanager.com/pone/ and select the 'Submissions Needing Revision' folder to locate your manuscript file.

- A letter that responds to each point raised by the academic editor and reviewer(s). You should upload this letter as a separate file labeled 'Response to Reviewers'.
- A marked-up copy of your manuscript that highlights changes made to the original version. You should upload this as a separate file labeled 'Revised Manuscript with Track Changes'.
- An unmarked version of your revised paper without tracked changes. You should upload this as a separate file labeled 'Manuscript'.

If you would like to make changes to your financial disclosure, please include your updated statement in your cover letter. Guidelines for resubmitting your figure files are available below the reviewer comments at the end of this letter.

If applicable, we recommend that you deposit your laboratory protocols in protocols.io to enhance the reproducibility of your results. Protocols.io assigns your protocol its own identifier (DOI) so that it can be cited independently in the future. For instructions see: https://journals.plos.org/plosone/s/submission-guidelines#loc-laboratory-protocols . Additionally, PLOS ONE offers an option for publishing peer-reviewed Lab Protocol articles, which describe protocols hosted on protocols.io. Read more information on sharing protocols at https://plos.org/protocols?utm_medium=editorial-email&utm_source=authorletters&utm_campaign=protocols .

As the corresponding author, your ORCID iD is verified in the submission system and will appear in the published article. PLOS supports the use of ORCID, and we encourage all coauthors to register for an ORCID iD and use it as well. Please encourage your coauthors to verify their ORCID iD within the submission system before final acceptance, as unverified ORCID iDs will not appear in the published article. *Only*   the individual author can complete the verification step; PLOS staff *cannot*   verify ORCID iDs on behalf of authors.

We look forward to receiving your revised manuscript.

Kind regards,

Md. Kamrujjaman, Ph.D

Academic Editor

PLOS One

Journal Requirements:

1. If the reviewer comments include a recommendation to cite specific previously published works, please review and evaluate these publications to determine whether they are relevant and should be cited. There is no requirement to cite these works unless the editor has indicated otherwise.

2. Please review your reference list to ensure that it is complete and correct. If you have cited papers that have been retracted, please include the rationale for doing so in the manuscript text, or remove these references and replace them with relevant current references. Any changes to the reference list should be mentioned in the rebuttal letter that accompanies your revised manuscript. If you need to cite a retracted article, indicate the article’s retracted status in the References list and also include a citation and full reference for the retraction notice.

[Note: HTML markup is below. Please do not edit.]

Reviewers' comments:

Reviewer's Responses to Questions

**Comments to the Author**

Reviewer #1: (No Response)

**********

2. Is the manuscript technically sound, and do the data support the conclusions?

Reviewer #1: (No Response)

**********

3. Has the statistical analysis been performed appropriately and rigorously?

Reviewer #1: (No Response)

**********

4. Have the authors made all data underlying the findings in their manuscript fully available?

The PLOS Data policy

Reviewer #1: (No Response)

**********

5. Is the manuscript presented in an intelligible fashion and written in standard English?

Reviewer #1: (No Response)

**********

Reviewer #1: Could you please recheck Comment 1 carefully? It appears that this comment was not part of the current revision round, and there may have been a mismatch in the included comment. Please address the appropriate comment accordingly.

**********

what does this mean? ). If published, this will include your full peer review and any attached files.

If you choose “no”, your identity will remain anonymous but your review may still be made public.

**Do you want your identity to be public for this peer review?**   For information about this choice, including consent withdrawal, please see our Privacy Policy

Reviewer #1: No

**********

[NOTE: If reviewer comments were submitted as an attachment file, they will be attached to this email and accessible via the submission site. Please log into your account, locate the manuscript record, and check for the action link "View Attachments". If this link does not appear, there are no attachment files.]

To ensure your figures meet our technical requirements, please review our figure guidelines: https://journals.plos.org/plosone/s/figures

You may also use PLOS’s free figure tool, NAAS, to help you prepare publication quality figures: https://journals.plos.org/plosone/s/figures#loc-tools-for-figure-preparation.

NAAS will assess whether your figures meet our technical requirements by comparing each figure against our figure specifications.

# Author comment: Author response to Decision Letter 3

**DOI:** 10.1371/journal.pone.0353069.r006

11 Jun 2026

Response to Academic Editor

Thank you for the opportunity to revise our manuscript. We have carefully addressed the comments and prepared a revised manuscript, a marked-up version, and a clean version for submission.

Response to Reviewer #1

Thank you for handling and reading our manuscript. We have addressed all of your comments as follows:

1. Could you please recheck Comment 1 carefully? It appears that this comment was not part of the current revision round, and there may have been a mismatch in the included comment. Please address the appropriate comment accordingly.

Thank you for your meticulous observation. Actually, it was mistakenly placed. However, the question and the answer is included below.

‘‘The interval coverage during the outbreak period appears to be consistently zero across all forecast horizons (Table 8). This requires an explanation.’’

Thank you for pointing this out. We have added an explanation in the Sensitivity and Uncertainty Analysis section. We now clarify that the zero outbreak-period coverage in Table 8 means that none of the observed outbreak counts fell within TFT’s predicted 80% intervals. The model still produced prediction intervals, but these intervals were below the observed outbreak magnitudes and shallow learning from dominant low dengue count period.

Attachment Submitted filename: Response_to_Reviewers_auresp_3.pdf

# Peer review: Decision Letter 3

**DOI:** 10.1371/journal.pone.0353069.r007

**Authors:** Md. Kamrujjaman

4 Aug 2026

Tactical vs. strategic: An adaptable framework for horizon-dependent dengue forecasting using data-driven approaches with serotype and climate covariates in Bangladesh

PONE-D-26-03317R3

Dear Dr. Aziz,

We’re pleased to inform you that your manuscript has been judged scientifically suitable for publication and will be formally accepted for publication once it meets all outstanding technical requirements.

Within one week, you’ll receive an e-mail detailing the required amendments. When these have been addressed, you’ll receive a formal acceptance letter and your manuscript will be scheduled for publication.

An invoice will be generated when your article is formally accepted. Please note, if your institution has a publishing partnership with PLOS and your article meets the relevant criteria, all or part of your publication costs will be covered. Please make sure your user information is up-to-date by logging into Editorial Manager at Editorial Manager®  and clicking the ‘Update My Information' link at the top of the page. For questions related to billing, please contact billing support .

If your institution or institutions have a press office, please notify them about your upcoming paper to help maximize its impact. If they’ll be preparing press materials, please inform our press team as soon as possible -- no later than 48 hours after receiving the formal acceptance. Your manuscript will remain under strict press embargo until 2 pm Eastern Time on the date of publication. For more information, please contact onepress@plos.org.

Kind regards,

Md. Kamrujjaman, Ph.D

Academic Editor

PLOS One

Additional Editor Comments (optional):

Reviewers' comments:

Reviewer's Responses to Questions

**Comments to the Author**

Reviewer #1: (No Response)

Reviewer #3: All comments have been addressed

**********

2. Is the manuscript technically sound, and do the data support the conclusions?

Reviewer #1: (No Response)

Reviewer #3: Yes

**********

3. Has the statistical analysis been performed appropriately and rigorously?

Reviewer #1: (No Response)

Reviewer #3: Yes

**********

4. Have the authors made all data underlying the findings in their manuscript fully available?

The PLOS Data policy

Reviewer #1: (No Response)

Reviewer #3: Yes

**********

5. Is the manuscript presented in an intelligible fashion and written in standard English?

Reviewer #1: (No Response)

Reviewer #3: Yes

**********

Reviewer #1: (No Response)

Reviewer #3: This paper has a significant strength: it does not merely compare models, but rather integrates predictive performance, interpretability, and causal analysis within a single framework. It stands out for reporting multiple metrics, evaluating results by time horizon, regime, and district, and acknowledging the actual limitations of the validation context. For these reasons, I recommend that the manuscript be accepted for publication.

**********

what does this mean? ). If published, this will include your full peer review and any attached files.

If you choose “no”, your identity will remain anonymous but your review may still be made public.

**Do you want your identity to be public for this peer review?**   For information about this choice, including consent withdrawal, please see our Privacy Policy

Reviewer #1: No

Reviewer #3: **Yes:**  IVAN ANTONIO GARCIA-MONTALVO

**********

# Editor report: Acceptance letter

**DOI:** 10.1371/journal.pone.0353069.r008

**Authors:** Md. Kamrujjaman

PONE-D-26-03317R3

PLOS One

Dear Dr. Aziz,

I'm pleased to inform you that your manuscript has been deemed suitable for publication in PLOS One. Congratulations! Your manuscript is now being handed over to our production team.

At this stage, our production department will prepare your paper for publication. This includes ensuring the following:

* All references, tables, and figures are properly cited

* All relevant supporting information is included in the manuscript submission,

* There are no issues that prevent the paper from being properly typeset

You will receive further instructions from the production team, including instructions on how to review your proof when it is ready. Please keep in mind that we are working through a large volume of accepted articles, so please give us a few days to review your paper and let you know the next and final steps.

Lastly, if your institution or institutions have a press office, please let them know about your upcoming paper now to help maximize its impact. If they'll be preparing press materials, please inform our press team within the next 48 hours. Your manuscript will remain under strict press embargo until 2 pm Eastern Time on the date of publication. For more information, please contact onepress@plos.org.

You will receive an invoice from PLOS for your publication fee after your manuscript has reached the completed accept phase. If you receive an email requesting payment before acceptance or for any other service, this may be a phishing scheme. Learn how to identify phishing emails and protect your accounts at https://explore.plos.org/phishing.

If we can help with anything else, please email us at customercare@plos.org.

Thank you for submitting your work to PLOS One and supporting open access.

Kind regards,

PLOS ONE Editorial Office Staff

on behalf of

Dr. Md. Kamrujjaman

Academic Editor

PLOS One
